# Supplementary material for: Plastic Creep Constraint in Nylon Instrument Strings
Source: Materials (Basel). 2025 Jan 7;18(2):223. doi: 10.3390/ma18020223 (PMC11766866; doi:10.3390/ma18020223)
Supplement: Supplementary file 1 [file materials-18-00223-s001.zip › Nylon string creep data file analysis and plots.html]

Nylon string creep data file analysis and plots


# Nylon string creep data file analysis and plots¶

PROCESSING SEQUENCE

> Initial definitions  
> Load data for strings
>
> Single-step curve fitting for strings (if not done already)  
> -> make sure save\_results is set True  
> -> then need to reload data, to apply step start level corrections for N7 and N21
>
> Elastic strain study  
> Recoverable creep study  
> -> including multi-step fitting exercise  
> Plastic creep study - for strings N7 and N28  
> -> including multi-step fitting exercises
>
> Additional plots (at any point once data loaded)  
> -> Stress-Strain comparison  
> -> Thermal compensation for stress steps

## Initial definitions¶

In [1]:

```
from pathlib import Path
import xlrd
import numpy as np
import numpy.polynomial.polynomial as poly
from scipy.optimize import curve_fit
from scipy.interpolate import PchipInterpolator
import matplotlib.pyplot as plt
import copy
#
# enable plots to be shown in cells
%matplotlib inline
#
from datetime import datetime
FMT_time = '%H:%M:%S'
FMT_date = '%Y/%m/%d'


def read_data_files(string, step_range, step_starts, step_indeces, time_array, length_adj_array, 
                    temperature_array, L_v, mu_threshold):
    # reads all tuned data records plus values at very start of each creep test
    filepath = "./data_files/string_" + string + "_nylon/"
    prev_mu = 0.0
    bad_mu_count = 0
    for step in step_range:
        new_step = True
        step_starts.append(len(time_array)) # record array index for start of each test step
        filename = "string_test_data_" + string + "_step_" + str(step) + ".csv"
        f = open(filepath + filename, "r")
        data = f.readlines()
        for line in data[1:]:                   # skip headers row
            words = line.split(',')
            if(((words[2] == 'ok') or (new_step == True)) and (words[3] == 'ok')): 
            # skip rows where not tuned (unless start of step) or fundamental not found
            # words[2] == 'ok' if tuned to target frequency, words[3] == 'ok' if fundamental frequency identified
                frequency = float(words[4])
                tension = float(words[5])
                mu = tension / (4.0 * frequency * frequency * L_v * L_v)
                if(mu < (mu_threshold * prev_mu)):
                    bad_mu_count +=1
                else:
                    if(new_step and (step > 1)):
                        step_indeces.append(step - 2)  # to associate stress from previous step with first point of new step
                    else:
                        step_indeces.append(step - 1)
                    new_step = False
                    line_date = datetime.strptime(words[0],FMT_date)
                    line_time = datetime.strptime(words[1],FMT_time)
                    if(len(time_array)==0):
                        start_date = line_date
                        start_time = line_time
                        days = 0.0
                    else:
                        date_diff = line_date - start_date
                        time_diff = line_time - start_time # could be negative
                        days = date_diff.days + time_diff.days + time_diff.seconds / (24.0 * 3600.0)
                    time_array.append(days)
                    length_adj_array.append(float(words[6])) 
                    temperature_array.append(float(words[7]))
                    if(words[2] == 'ok'):
                        prev_mu = mu
        # close file
        f.close()
    print("  bad_mu_count =", bad_mu_count)
    return 

def get_length_adj_offset(string):
    # read tension and length adjustment data
    time_array = []
    tension_array = []
    length_adj_array = []
    filepath = "./data_files/string_" + string + "_nylon/"
    filename = "string_test_data_" + string + "_step_1.csv"
    f = open(filepath + filename, "r")
    data = f.readlines()
    for line in data[1:]:                   # skip headers row
        words = line.split(',')
        line_date = datetime.strptime(words[0],FMT_date)
        line_time = datetime.strptime(words[1],FMT_time)
        if(len(time_array)==0):
            start_date = line_date
            start_time = line_time
            days = 0.0
        else:
            date_diff = line_date - start_date
            time_diff = line_time - start_time # could be negative
            days = date_diff.days + time_diff.days + time_diff.seconds / (24.0 * 3600.0)
        time_array.append(days)
        tension_array.append(float(words[5]))
        length_adj_array.append(float(words[6]))
    # close file
    f.close()
    # fit line to first two points with non-zero length adjustment between them
    index = 0
    while((index < len(length_adj_array)) and (length_adj_array[index] == 0)):
        index += 1  # find first non-zero length adjustment value
    first_fit = poly.polyfit(tension_array[index-1:index+1], length_adj_array[index-1:index+1], 1)
    L_offset = -first_fit[0]
    return L_offset

def read_fitted_parameters(fileroot, string):
    # data_array format: row=step(0..n); columns: stages A0 B A1 X1 A2 X2 A3 X3
    # for A0 + B*t + A1*(1-np.exp(-X1*t)) + A2*(1-np.exp(-X2*t)) + A3*(1-np.exp(-X3*t))
    sdict = globals()[string]
    datafile = fileroot + str(string) + ".csv"
    if(Path(datafile).exists()):
        test_steps = sdict['test_steps']
        data_array = np.zeros((test_steps,data_cols))
        f = open(datafile, "r")
        data = f.readlines()
        for line in data[1:]:         # skip headers row
            words = line.split(',')
            step_index = int(words[0]) - 1
            stages = int(words[1])
            data_array[step_index][0] = stages
            data_array[step_index][1] = float(words[2])  # A0
            data_array[step_index][2] = float(words[3])  # B
            for i in range(max_stages):
                if(i < stages):
                    data_array[step_index][3+(2*i)] = float(words[4+(2*i)])  # An
                    data_array[step_index][4+(2*i)] = float(words[5+(2*i)])  # Xn
                else:
                    data_array[step_index][3+(2*i)] = np.nan
                    data_array[step_index][4+(2*i)] = np.nan
        f.close()
    else:
        print('\n*** File of fitted coefficients not found for string %s ***\n' % (string))
        data_array = np.array([])
    return data_array

def get_fitted_components(fileroot, string_list):
    # reads fitted coefficients into sdict['fitted_values']
    # data_array format: row=step(0..n); columns: stages A0 B A1 X1 A2 X2 A3 X3
    # for A0 + B*t + A1*(1-np.exp(-X1*t)) + A2*(1-np.exp(-X2*t)) + A3*(1-np.exp(-X3*t))
    # creates sdict['fitted_steps'] arrays containing the elastic strain step at the start of each step response
    for string in string_list:
        sdict = globals()[string]
        test_steps = sdict['test_steps']
        step_starts = sdict['step_starts']
        strain_array = sdict['strain_array']
        stress_n = sdict['stress_n']
        stress_n_step = sdict['stress_n_step']
        #
        # read in fitted parameters from single-step curve fitting
        sdict['fitted_values'] = read_fitted_parameters(fileroot, string)
        #
        if(len(sdict['fitted_values']) > 0):
            # extract initial strain steps
            sdict['fitted_steps'] = np.copy(sdict['fitted_values'][:,1])
            sdict['fitted_steps'][1:] -= np.array([strain_array[step_starts[i]] for i in range(1,test_steps,1)])
            #
            # calculate equivalent model values
            sdict['model_values'] = np.copy(sdict['fitted_values'])
            for step_index in range(test_steps):
                # E_el = stress_step / A0 (GPa)
                sdict['model_values'][step_index,1] = \
                    stress_n_step[step_index] / (1000 * sdict['fitted_steps'][step_index])
                # eta_f = total stress / B (GPa.day)
                sdict['model_values'][step_index,2] = \
                    stress_n[step_index] / (1000 * sdict['fitted_values'][step_index,2])
                # E_i and eta_i
                for stage in range(int(sdict['fitted_values'][step_index,0])):
                    # E_i = stress_step / Ai (GPa)
                    sdict['model_values'][step_index,3+2*stage] = \
                        stress_n_step[step_index] / (1000 * sdict['fitted_values'][step_index,3+2*stage])
                    # eta_i = E_i / Xi (GPa.day)
                    sdict['model_values'][step_index,4+2*stage] = \
                        sdict['model_values'][step_index,3+2*stage] / sdict['fitted_values'][step_index,4+2*stage]

def fit_func(t, coeffs):
    # data_array format: row=step(0..n); columns: stages A0 B A1 X1 A2 X2 A3 X3
    # for fitted function: A0 + B*t + A1*(1-np.exp(-X1*t)) + A2*(1-np.exp(-X2*t)) + A3*(1-np.exp(-X3*t))
    value = coeffs[1] + coeffs[2]*t  # A0 + B*t
    for i in range(int(coeffs[0])):       # for each K-V stage
        value += coeffs[2*i+3] * (1 - np.exp(-coeffs[2*i+4]*t))
    return value

def elastic_strain_from_stress(sigma, K, G):
    # K = E estimate at zero stress
    # G = stress scaler
    return np.log(1.0 + sigma * G / K) / G

def r_squared(y_act, y_est):
    y_mean = np.mean(y_act)
    sumsq_tot = np.sum((y_act - y_mean)**2)
    sumsq_res = np.sum((y_act - y_est)**2)
    return (1.0 - sumsq_res/sumsq_tot)


T_0 = 20.0                       # (°C)
def_thermal_adj_rate = -0.022    # (mm/°C) default thermal adjustment rate
max_stages = 3                   # maximum number of K-V stages used in single-step fitting
data_cols = 3 + 2 * max_stages

# coefficients for estimating E_T in GPa (from nylon paper)
Et_coeff_a = 31.9859792151428    # constant (GPa)
Et_coeff_b = 0.0352816913659568  # times notional stress in MPa
Et_coeff_c = -0.0269321235467744 # times unstretched density in kg/m^3

fig_size_1 = (6,4.5)
fig_size_2 = (9.5,4.5)
fig_size_3 = (9.5,7)
fig_size_4 = (9.5,10)
save_format = 'eps'
save_path = './'
filename_start = "single_step_fitting_results_"
```

## Load string data¶

In [2]:

```
def N21_10_tail_function(t, A0, A1, X1):
    return A0 + A1*(1-np.exp(-X1*t))

string_list = ['N7','N20','N21','N27','N26','N28','N34','N33']  # DO NOT CHANGE order or alignment offset calculations won't work  

N7   = {'rig':1, 'start_row':6,   'stop_row':17, 'style':'-', 'leg':'N7',  'pen':'red'}
N20  = {'rig':1, 'start_row':21,  'stop_row':30, 'style':'-', 'leg':'N20', 'pen':'black'}
N21  = {'rig':1, 'start_row':34,  'stop_row':51, 'style':'-', 'leg':'N21', 'pen':'blue'}
N27  = {'rig':1, 'start_row':55,  'stop_row':72, 'style':'-', 'leg':'N27', 'pen':'magenta'}
N26  = {'rig':1, 'start_row':76,  'stop_row':82, 'style':'-', 'leg':'N26', 'pen':'lime'}
N28  = {'rig':2, 'start_row':86,  'stop_row':105,'style':'-', 'leg':'N28', 'pen':'green'}
N34  = {'rig':1, 'start_row':109, 'stop_row':113,'style':'-', 'leg':'N34', 'pen':'cyan'}
N33  = {'rig':2, 'start_row':119, 'stop_row':128,'style':'-', 'leg':'N33', 'pen':'orange'}

# strings from previous nylon study
N8   = {'rig':1, 'start_row':132, 'stop_row':142,'style':'-', 'leg':'N8',  'pen':'black'}

# connect to data source
file_location = "./creep_study_summary.xls"
workbook1 = xlrd.open_workbook(file_location)
data_sheet = workbook1.sheet_by_index(0)
# columns
param_col =      2  # individual parameters
freq_col =       4  # Hz         target frequency
adj_rate_col =   7  # mm/°C      thermal adjustment rate for applying temperature corrections to length adjustment
length_adj_col = 8  # m          measured length adjustment for N8 (at start of Young's modulus Et test)

load_list = string_list
for string in (load_list + ['N8']):
    print('\n\nSTRING', string)
    sdict = globals()[string]
    # read data
    print('Reading summary data for string', string)
    start_row = sdict['start_row']
    start_row1 = start_row + 1
    stop_row = sdict['stop_row']
    sdict['test_steps'] = test_steps = stop_row - start_row - 1
    sdict['step_range'] = np.arange(1,test_steps+1,1)
    #
    L_v = data_sheet.cell_value(start_row, param_col)
    L_total = data_sheet.cell_value(start_row+1, param_col)
    rho_0 = data_sheet.cell_value(start_row+2, param_col)
    #
    sdict['freq'] = np.array([data_sheet.cell_value(i, freq_col) for i in range(start_row1, stop_row)])
    # calculate notional stress values
    sdict['stress_n'] = 4.0 * (L_v * sdict['freq'])**2 * rho_0 / 1e6 # values in MPa
    # read thermal adjustment gradients
    if(string in ['N7','N8']):
        sdict['adj_rate'] = np.array([data_sheet.cell_value(i, adj_rate_col) 
                                      if(data_sheet.cell(i, adj_rate_col).ctype not in [0,1,6]) 
                                      else np.nan for i in range(start_row1, stop_row)])
    #
    if(string == 'N8'):
        # read N8 length adjustments values for stres-strain comparisons
        sdict['length_adj'] = np.array([data_sheet.cell_value(i, length_adj_col) for i in range(start_row1, stop_row)])
        sdict['strain_array'] = np.expm1(sdict['length_adj'] / L_total)
    else:
        # Young's modulus estimates - based on notional stress
        sdict['E0_est'] = Et_coeff_a + Et_coeff_c * rho_0 # GPa
        sdict['Esigma_est'] = sdict['E0_est'] + Et_coeff_b * sdict['stress_n'] # GPa
        print("  Young's modulus estimates for string %s:" % (string))
        print(sdict['E0_est'],sdict['Esigma_est'])
        # elastic strain estimates - based on notional stress
        sdict['el_strain_est'] = elastic_strain_from_stress(sdict['stress_n'] / 1000.0, sdict['E0_est'], Et_coeff_b * 1000.0) # scale stress and Et_coeff_b for GPa
        # extract stress and strain steps
        sdict['stress_n_step'] = np.copy(sdict['stress_n'])
        sdict['est_strain_step'] = np.copy(sdict['el_strain_est'])
        if(test_steps > 1):
            sdict['stress_n_step'][1:] -= sdict['stress_n'][:-1]
            sdict['est_strain_step'][1:] -= sdict['el_strain_est'][:-1]
        print('notional stress', sdict['stress_n'])
        print('notional stress steps', sdict['stress_n_step'])
        print('elastic strain', sdict['el_strain_est'])
        #
        # identify steps matching and exceeding previous maximum stress
        max_stress = 0
        sdict['exceeding_steps'] = []
        sdict['matching_steps'] = []
        for i in range(sdict['test_steps']):
            if(sdict['stress_n'][i] > max_stress):
                max_stress = sdict['stress_n'][i]
                sdict['exceeding_steps'].append(1)
                sdict['matching_steps'].append(0)
            elif(np.abs(sdict['stress_n'][i] - max_stress) < 5):
                sdict['exceeding_steps'].append(0)
                sdict['matching_steps'].append(1)
            else:
                sdict['exceeding_steps'].append(0)
                sdict['matching_steps'].append(0)
        print('  steps exceeding previous max stress', sdict['exceeding_steps'])
        print('  steps matching previous max stress ', sdict['matching_steps'])
        #
        # read raw data files
        print('Reading data files for string', string)
        step_starts = []
        step_indeces = []
        time_array = []
        length_adj_array_raw = []
        temperature_array = []
        mu_threshold = 0.98
        read_data_files(string, sdict['step_range'], step_starts, step_indeces, time_array, length_adj_array_raw, 
                        temperature_array, L_v, mu_threshold)
        step_starts.append(len(time_array))
        sdict['step_starts'] = np.array(step_starts)    # array index for start of each step
        sdict['step_indeces'] = np.array(step_indeces)  # full-length array giving step-1 values
        sdict['time_array'] = np.array(time_array)
        sdict['length_adj_array_raw'] = np.array(length_adj_array_raw)
        sdict['temperature_array'] = np.array(temperature_array)
        # time step array
        sdict['time_steps'] = np.copy(sdict['time_array'])
        sdict['time_steps'][1:] -= sdict['time_array'][:-1]
        sdict['time_steps'][0] = 0.0
        print('  average time step = %.2f minutes' % (1440*np.average(sdict['time_steps'])))
        #
        # add length adjustment offset correction
        adj_offset = get_length_adj_offset(string)
        print('Length adjustment correction offset from initial adjustment gradient = %.6f m' % (adj_offset))
        sdict['length_adj_array0'] = adj_offset + sdict['length_adj_array_raw']
        #
        # add thermal corrections
        sdict['length_adj_array'] = (sdict['length_adj_array0'] 
                                     - def_thermal_adj_rate * (sdict['temperature_array'] - T_0) / 1000.0) # metres
        #
        # convert to strain, using equation (7) from nylon paper: "Mechanical Properties of Nylon Harp Strings"
        sdict['strain_array'] = np.expm1(sdict['length_adj_array'] / L_total)
        #
        # Read in fitted parameters from single-step curve fitting
        fitted_values = read_fitted_parameters(save_path+filename_start, string)
        if((len(fitted_values) > 0) and (string in ['N7','N21'])):
            # For strings N7 and N21, readjust the start of each step using the fitted curves, 
            #   but only if initial curve fitting has been run
            for i in range (1,test_steps,1):
                sdict['strain_array'][step_starts[i]] = \
                    fit_func(time_array[step_starts[i]]-time_array[step_starts[i-1]], fitted_values[i-1])
            #
            if(string == 'N21'):
                # readjust start of N21 step 11 using separate fit to tail section of step 10 strain response
                start_index = step_starts[9] + 710
                stop_index = step_starts[10]
                start_time = N21['time_array'][start_index]
                time_fit_array = N21['time_array'][start_index:stop_index] - start_time
                strain_fit_array = N21['strain_array'][start_index:stop_index]
                popt_2110, pcov_2110 = curve_fit(N21_10_tail_function, time_fit_array, strain_fit_array, 
                                                 p0=[0.078,0.00085,0.847], bounds=(0,np.inf))
                N21['strain_array'][step_starts[10]] = \
                                N21_10_tail_function(N21['time_array'][step_starts[10]] - start_time, *popt_2110)
                #
                # create version without additional strain due to extra heating in step 10
                # extrapolate step 9 fitted function across step 10 time span, but don't adjust timeline
                N21m = copy.deepcopy(N21)
                time_sub_array = N21m['time_array'][step_starts[9]:step_starts[10]+1] - N21m['time_array'][step_starts[8]]
                    # include first point of step 11
                tail_array = fit_func(time_sub_array, fitted_values[8])
                N21m['strain_array'][step_starts[9]:step_starts[10]] = tail_array[:-1]
                N21m['strain_array'][step_starts[10]:] += tail_array[-1] - N21m['strain_array'][step_starts[10]]
                

plt.figure(figsize=fig_size_3)
for string in load_list:
    sdict = globals()[string]
    plt.plot(sdict['time_array'], 1440 * sdict['time_steps'], color=sdict['pen'], ls='none', marker='.', label='string '+string)
plt.axis([0,160,0,70])
plt.xlabel('Time (days)')
plt.ylabel('Time step (minutes)')
plt.legend()
plt.tight_layout()  
plt.show()

plt.figure(figsize=fig_size_3)
for string in load_list:
    sdict = globals()[string]
    plt.plot(sdict['time_array'], sdict['temperature_array'], color=sdict['pen'], ls='-', label='string '+string)
plt.xlabel('Time (days)')
plt.ylabel('Temperature')
plt.legend()
plt.tight_layout()  
plt.show()
```

```
STRING N7
Reading summary data for string N7
  Young's modulus estimates for string N7:
2.515452217787285 [ 3.68230507  4.64752696  5.61269944  6.57777885  7.54313953  8.50835315
  9.47343696 10.43866742 11.40384818 12.36901718]
notional stress [ 33.07247479  60.43006043  87.78624554 115.13979263 142.50131211
 169.85866325 197.21233508 224.57016365 251.92658337 279.28266999]
notional stress steps [33.07247479 27.35758564 27.35618511 27.35354709 27.36151948 27.35735114
 27.35367183 27.35782857 27.35641972 27.35608663]
elastic strain [0.01080125 0.01739947 0.02274775 0.02724485 0.03112623 0.03453904
 0.03758434 0.04033436 0.04284087 0.04514359]
  steps exceeding previous max stress [1, 1, 1, 1, 1, 1, 1, 1, 1, 1]
  steps matching previous max stress  [0, 0, 0, 0, 0, 0, 0, 0, 0, 0]
Reading data files for string N7
  bad_mu_count = 1
  average time step = 62.29 minutes
Length adjustment correction offset from initial adjustment gradient = 0.002888 m


STRING N20
Reading summary data for string N20
  Young's modulus estimates for string N20:
3.008774324536077 [ 9.99511685 11.14639182  9.99511685  8.93160724  9.99511685 11.14639182
  9.99511685  8.93160724]
notional stress [198.01608871 230.64703474 198.01608871 167.87270358 198.01608871
 230.64703474 198.01608871 167.87270358]
notional stress steps [198.01608871  32.63094603 -32.63094603 -30.14338512  30.14338512
  32.63094603 -32.63094603 -30.14338512]
elastic strain [0.03402796 0.03711792 0.03402796 0.03083933 0.03402796 0.03711792
 0.03402796 0.03083933]
  steps exceeding previous max stress [1, 1, 0, 0, 0, 0, 0, 0]
  steps matching previous max stress  [0, 0, 0, 0, 0, 1, 0, 0]
Reading data files for string N20
  bad_mu_count = 0
  average time step = 17.55 minutes
Length adjustment correction offset from initial adjustment gradient = 0.018329 m


STRING N21
Reading summary data for string N21
  Young's modulus estimates for string N21:
3.4841530238411167 [10.35588276  9.3098203   8.40414887  9.3098203  10.35588276 11.48827075
 10.35588276  9.3098203  10.35588276 10.35588276 11.48827075 10.35588276
  9.3098203  10.35588276 11.48827075 12.3032481 ]
notional stress [194.76758247 165.11870755 139.44897917 165.11870755 194.76758247
 226.86320922 194.76758247 165.11870755 194.76758247 194.76758247
 226.86320922 194.76758247 165.11870755 194.76758247 226.86320922
 249.96236659]
notional stress steps [194.76758247 -29.64887493 -25.66972838  25.66972838  29.64887493
  32.09562674 -32.09562674 -29.64887493  29.64887493   0.
  32.09562674 -32.09562674 -29.64887493  29.64887493  32.09562674
  23.09915738]
elastic strain [0.03087521 0.02785708 0.0249563  0.02785708 0.03087521 0.03381645
 0.03087521 0.02785708 0.03087521 0.03087521 0.03381645 0.03087521
 0.02785708 0.03087521 0.03381645 0.03575901]
  steps exceeding previous max stress [1, 0, 0, 0, 0, 1, 0, 0, 0, 0, 0, 0, 0, 0, 0, 1]
  steps matching previous max stress  [0, 0, 0, 0, 1, 0, 0, 0, 0, 0, 1, 0, 0, 0, 1, 0]
Reading data files for string N21
  bad_mu_count = 0
  average time step = 5.39 minutes
Length adjustment correction offset from initial adjustment gradient = 0.011854 m


STRING N27
Reading summary data for string N27
  Young's modulus estimates for string N27:
2.9565551173850437 [ 9.95548758 11.10883724  9.95548758  8.89006145  9.95548758 11.10883724
  9.95548758  8.89006145  7.96762511  7.19894084  6.54076943  7.96762511
  8.89006145  9.95548758 11.10883724  9.95548758]
notional stress [198.3729293  231.06267887 198.3729293  168.17522343 198.3729293
 231.06267887 198.3729293  168.17522343 142.03032217 120.24326374
 101.5885059  142.03032217 168.17522343 198.3729293  231.06267887
 198.3729293 ]
notional stress steps [198.3729293   32.68974957 -32.68974957 -30.19770588  30.19770588
  32.68974957 -32.68974957 -30.19770588 -26.14490126 -21.78705843
 -18.65475784  40.44181627  26.14490126  30.19770588  32.68974957
 -32.68974957]
elastic strain [0.03441159 0.0375185  0.03441159 0.03120341 0.03441159 0.0375185
 0.03441159 0.03120341 0.02809847 0.02522297 0.02250544 0.02809847
 0.03120341 0.03441159 0.0375185  0.03441159]
  steps exceeding previous max stress [1, 1, 0, 0, 0, 0, 0, 0, 0, 0, 0, 0, 0, 0, 0, 0]
  steps matching previous max stress  [0, 0, 0, 0, 0, 1, 0, 0, 0, 0, 0, 0, 0, 0, 1, 0]
Reading data files for string N27
  bad_mu_count = 2
  average time step = 5.14 minutes
Length adjustment correction offset from initial adjustment gradient = 0.018031 m


STRING N26
Reading summary data for string N26
  Young's modulus estimates for string N26:
2.898259657872611 [ 9.91124704  8.84368136  9.91124704 11.06691279 12.04740724]
notional stress [198.77129205 168.51294463 198.77129205 231.52668756 259.31714792]
notional stress steps [198.77129205 -30.25834742  30.25834742  32.75539551  27.79046036]
elastic strain [0.0348498  0.0316196  0.0348498  0.03797577 0.04038182]
  steps exceeding previous max stress [1, 0, 0, 1, 1]
  steps matching previous max stress  [0, 0, 1, 0, 0]
Reading data files for string N26
  bad_mu_count = 0
  average time step = 5.12 minutes
Length adjustment correction offset from initial adjustment gradient = 0.009994 m


STRING N28
Reading summary data for string N28
  Young's modulus estimates for string N28:
2.8866480087081037 [ 4.03387834  4.97925585  5.9214125   4.97925585  5.9214125   6.86444068
  7.82482358  5.9214125   7.82482358  8.76891275  9.73096291  8.76891275
  7.82482358  8.76891275  9.73096291 10.66250969 11.61705716 12.55015753]
notional stress [ 32.51630756  59.31143761  86.01527799  59.31143761  86.01527799
 112.74382028 139.96425281  86.01527799 139.96425281 166.72286699
 193.99055535 166.72286699 139.96425281 166.72286699 193.99055535
 220.3936768  247.44871392 273.89586922]
notional stress steps [ 32.51630756  26.79513005  26.70384038 -26.70384038  26.70384038
  26.72854229  27.22043253 -53.94897482  53.94897482  26.75861418
  27.26768836 -27.26768836 -26.75861418  26.75861418  27.26768836
  26.40312145  27.05503712  26.4471553 ]
elastic strain [0.00948459 0.01545233 0.02036408 0.01545233 0.02036408 0.02455264
 0.0282641  0.02036408 0.0282641  0.03149273 0.03444327 0.03149273
 0.0282641  0.03149273 0.03444327 0.03703444 0.03946462 0.04165439]
  steps exceeding previous max stress [1, 1, 1, 0, 0, 1, 1, 0, 0, 1, 1, 0, 0, 0, 0, 1, 1, 1]
  steps matching previous max stress  [0, 0, 0, 0, 1, 0, 0, 0, 1, 0, 0, 0, 0, 0, 1, 0, 0, 0]
Reading data files for string N28
  bad_mu_count = 1
  average time step = 5.04 minutes
Length adjustment correction offset from initial adjustment gradient = 0.006223 m


STRING N34
Reading summary data for string N34
  Young's modulus estimates for string N34:
2.8516628777691437 [ 9.87588464 11.0334017  12.01546684]
notional stress [199.08971172 231.89757941 259.73255842]
notional stress steps [199.08971172  32.80786769  27.83497901]
elastic strain [0.03520788 0.03834921 0.04076597]
  steps exceeding previous max stress [1, 1, 1]
  steps matching previous max stress  [0, 0, 0]
Reading data files for string N34
  bad_mu_count = 0
  average time step = 5.05 minutes
Length adjustment correction offset from initial adjustment gradient = 0.008196 m


STRING N33
Reading summary data for string N33
  Young's modulus estimates for string N33:
2.946298289754896 [ 9.90575824 11.05260324  9.90575824  8.84634088  9.90575824 11.05260324
 12.02561395 12.97570893]
notional stress [197.25414733 229.75953353 197.25414733 167.22675022 197.25414733
 229.75953353 257.33788001 284.26671897]
notional stress steps [197.25414733  32.50538619 -32.50538619 -30.02739711  30.02739711
  32.50538619  27.57834648  26.92883896]
elastic strain [0.03436816 0.03747316 0.03436816 0.03116218 0.03436816 0.03747316
 0.03986457 0.0420198 ]
  steps exceeding previous max stress [1, 1, 0, 0, 0, 0, 1, 1]
  steps matching previous max stress  [0, 0, 0, 0, 0, 1, 0, 0]
Reading data files for string N33
  bad_mu_count = 0
  average time step = 5.02 minutes
Length adjustment correction offset from initial adjustment gradient = 0.009296 m


STRING N8
Reading summary data for string N8
```

### Plot measured data¶

In [3]:

```
# plot measured data

def smoothed_plot(ax, x_val, y_val, plt_color, style, width, str_label):
    rise_count = 5
    spline_pnts = 100
    x_rise = x_val[:rise_count]
    x_main = x_val[rise_count:]
    y_rise = y_val[:rise_count]
    y_main = y_val[rise_count:]
    x_plt_main = np.linspace(x_main.min(), x_main.max(), spline_pnts)
    x_y_spline = PchipInterpolator(x_main, y_main)
    x_plt = np.concatenate((x_rise, x_plt_main))
    y_plt = np.concatenate((y_rise, x_y_spline(x_plt_main)))
    ax.plot(x_plt, y_plt, plt_color, ls=style, lw=width, label=str_label)


N7['plt_include']  = True
N20['plt_include'] = True
N21['plt_include'] = True
N27['plt_include'] = True
N26['plt_include'] = True
N28['plt_include'] = True
N34['plt_include'] = True
N33['plt_include'] = True

# NOTE: following options only take effect after strain response adjustments made
show_N21_adj = False#True    # only for plot_choice 3 (full strain), shows N21 with effect of step 10 heating removed

make_alignment = True
align_string = 'N7'
apply_time_offsets = True

show_stress = True
show_target_freq = False     # ignored if show_stress == True
show_N28_levels = False#True
show_N21_10_insert = True

N7['plt_start_step'] = 1
N20['plt_start_step'] = 1
N21['plt_start_step'] = 1
N27['plt_start_step'] = 1
N26['plt_start_step'] = 1
N28['plt_start_step'] = 1
N34['plt_start_step'] = 1
N33['plt_start_step'] = 1

N7['plt_stop_step'] = N7['test_steps']
N20['plt_stop_step'] = N20['test_steps']
N21['plt_stop_step'] = N21['test_steps']
N27['plt_stop_step'] = N27['test_steps']
N26['plt_stop_step'] = N26['test_steps']
N28['plt_stop_step'] = N28['test_steps']
N34['plt_stop_step'] = N34['test_steps']
N33['plt_stop_step'] = N33['test_steps']

# these following indexed by step-1
# N28 step:         1,2,3,4,5,6,7,8,9,10,11,12,13,14,15,16,17,18
N28_7_include =    [0,1,0,0,1,1,0,0,1, 1, 0, 0, 0, 0, 1, 1, 1, 1]  # whether or not to show N7 step levels against N28 plot
N28_7_start_step = [0,1,2,2,2,5,6,6,6, 9,10,10,10,10,10,15,16,17]  # N28 step for start of comparison (step-1)
N28_7_N7_step =    [1,2,3,3,3,4,5,5,5, 6, 7, 7, 7, 7, 7, 8, 9,10]  # N7 step to use in making comparison (step)

plot_choice = 3
# 0 : raw length adjustment
# 1 : length adjustment with offset correction
# 2 : length adjustment with offset and thermal corrections
# 3 : strain

plt_labels = [' raw length adj. data',' with offset correction',' with thermal correction','']
y_params = ['length_adj_array_raw','length_adj_array0','length_adj_array','strain_array']
y_labels = ['Length Adjustment (m)','Length Adjustment (m)','Length Adjustment (m)','Strain']

compact_plot = (not N7['plt_include'] and not N28['plt_include'])
plot_set = '_'
subset = False
x_min = y1_min = 1000
x_max = y1_max = 0
#
if(make_alignment):
    sdict = globals()[align_string]
    alignment_step = 7 if(align_string == 'N7') else 11 if(align_string == 'N28') else 1 
    alignment_level = sdict[y_params[plot_choice]][sdict['step_starts'][alignment_step]-1]  # last point of step
#
if(show_stress or show_target_freq):
    fig, (ax2, ax1) = plt.subplots(2, 1, gridspec_kw={'height_ratios':[3,7]}, 
                                   figsize=fig_size_1 if(compact_plot) else fig_size_4)
    y2_param = 'stress_n' if(show_stress) else 'freq'
    y2_label = ('Stress (MPa)' if(show_stress) else 'Frequency (Hz)') if(compact_plot) else \
               ('Applied stress (MPa)' if(show_stress) else 'Target frequency (Hz)')
    y2_min = 1000
    y2_max = 0
else:
    fig=plt.figure(figsize=fig_size_3)
    ax1=fig.add_subplot(111)
#
for string in string_list:
    sdict = globals()[string]
    if(sdict['plt_include']):
        plot_set += string[1:]
        # indeces and plot data array setup
        start_index = 1 if (sdict['plt_start_step'] == 1) else sdict['step_starts'][sdict['plt_start_step']-1]
        stop_index = sdict['step_starts'][sdict['plt_stop_step']]
        plot_x = sdict['time_array']
        plot_y1 = sdict[y_params[plot_choice]]
        if(show_stress or show_target_freq):
            y_offset = -4 if(string == 'N20') else 0
            plot_y2 = [sdict[y2_param][sdict['step_indeces'][i]]+y_offset for i in range(len(sdict['time_array']))]
        #
        align_offset = 0.0
        if(make_alignment):
            alignment_step = 7 if(string == 'N7') else 11 if(string == 'N28') else 1
            align_offset = alignment_level - plot_y1[sdict['step_starts'][alignment_step]-1]
            print('alignment offset added to string %s: %f' % (string, align_offset))
        sdict['align_offset'] = align_offset
        time_offset = -0.63 if(apply_time_offsets and (string in ['N26','N28'])) else 0
        # main data plot
        for step in range(sdict['plt_start_step'], sdict['plt_stop_step']+1, 1):
            step_start = 1 if (step == 1) else sdict['step_starts'][step-1]
            step_stop = sdict['step_starts'][step] + (1 if(step < sdict['test_steps']) else 0)
            x_val = plot_x[step_start:step_stop] + time_offset
            y_val = plot_y1[step_start:step_stop] + align_offset
            style = '-' if(sdict['exceeding_steps'][step-1]==1) else '--' if(sdict['matching_steps'][step-1]==1) else ':'
            width = 1.5
            str_label = (string + ' (Rig ' + str(sdict['rig']) + ')' + plt_labels[plot_choice] 
                         if(step == sdict['plt_start_step']) else '')
            # smooth data for main part of step strain response to more clearly show dashed and dotted segments
            smoothed_plot(ax1, x_val, y_val, sdict['pen'], style, width, str_label)
#            ax1.plot(plot_x[step_start:step_stop] + time_offset - 2, plot_y1[step_start:step_stop] + align_offset + 0.002, 
#                     color=sdict['pen'], ls=style, lw=width, label=str_label)
            x_min = min(x_min, (plot_x[step_start] + time_offset))
            x_max = max(x_max, (plot_x[step_stop-1] + time_offset))
            y1_min = min(y1_min, (plot_y1[step_start] + align_offset), (plot_y1[step_stop-1] + align_offset))
            y1_max = max(y1_max, (plot_y1[step_start] + align_offset), (plot_y1[step_stop-1] + align_offset))
            if(show_stress or show_target_freq):
                ax2.plot(plot_x[step_start:step_stop] + time_offset, plot_y2[step_start:step_stop], 
                         color=sdict['pen'], ls=style, lw=width, label='string '+string)
                y2_min = min(y2_min, plot_y2[step_start])
                y2_max = max(y2_max, plot_y2[step_stop-1])
        # plot strain versions for N21 with step 10 removed
        if(show_N21_adj and (plot_choice == 3) 
           and (string == 'N21') and ('N21m' in globals()) and (sdict['plt_stop_step'] >= 10)):
            start_step = max(10, sdict['plt_start_step'])
            for step in range(start_step, sdict['plt_stop_step']+1, 1):
                step_start = sdict['step_starts'][step-1]
                step_stop = sdict['step_starts'][step] + (1 if(step < sdict['test_steps']) else 0)
                x_val = plot_x[step_start:step_stop] + time_offset
                y_val = N21m['strain_array'][step_start:step_stop] + align_offset
                style = '-' if(sdict['exceeding_steps'][step-1]==1) else '--' if(sdict['matching_steps'][step-1]==1) else ':'
                str_label = ('adjusted' if(step == start_step) else '')
                # smooth data for main part of step strain response to more clearly show dashed and dotted segments
                smoothed_plot(ax1, x_val, y_val, 'purple', style, 1, str_label)
                
#                start_ndx = N21m['step_starts'][9] if(N21['plt_start_step'] <= 10) else start_index
                
#                ax1.plot(plot_x[start_ndx:stop_index] + time_offset, 
#                         N21m['strain_array'][start_ndx:stop_index] + align_offset, color='purple', ls=':', lw=1, label='adjusted')
        # add comparison arrows for N28
        if(show_N28_levels and (string == 'N28')):
            end_step = N28['plt_stop_step'] + (1 if(N28['plt_stop_step'] < N28['test_steps']) else 0)
            for step in range(N28['plt_start_step'],end_step):
                if(N28_7_include[step-1] == 1):
                    start_step = N28_7_start_step[step-1]
                    x_start = N28['time_array'][N28['step_starts'][start_step]] + time_offset
                    x_stop_index = min(N28['step_starts'][step],len(N28['time_array'])-1)
                    x_stop = N28['time_array'][x_stop_index] + time_offset
                    y_start_N7 = N7[y_params[plot_choice]][N7['step_starts'][N28_7_N7_step[step-1]-1]-2]
                    y_stop_N7 = N7[y_params[plot_choice]][N7['step_starts'][N28_7_N7_step[step-1]]-2]
                    y_start = plot_y1[N28['step_starts'][start_step]-2] + align_offset
                    y_delta = y_stop_N7 - y_start_N7
                    y_stop = y_start + (y_stop_N7 - y_start_N7)
                    ax1.plot([x_start,x_stop], [y_start,y_start], 'r:', lw=1)
                    ax1.arrow(x_stop, y_start, 0, y_delta, length_includes_head=True, color='r', ls='-', width=0.08, 
                              shape='full', head_width=1.5, head_length=0.003, overhang=0.3)
    else:
        subset = True
#
if(N7['plt_include'] and make_alignment):
    N7_lo_value = N7[y_params[plot_choice]][N7['step_starts'][7]-1] + N7['align_offset']
    N7_hi_value = N7[y_params[plot_choice]][N7['step_starts'][8]-1] + N7['align_offset']
    ax1.plot([x_min,x_max], [N7_lo_value,N7_lo_value], color=N7['pen'], ls=':', lw=0.5)
    ax1.plot([x_min,x_max], [N7_hi_value,N7_hi_value], color=N7['pen'], ls=':', lw=0.5)

#if(N21['plt_include'] and make_alignment):
#    N21_lo_value = N21[y_params[plot_choice]][N21['step_starts'][3]-1] + N21['align_offset']
#    ax1.plot([x_min,x_max], [N21_lo_value,N21_lo_value], color=N21['pen'], ls=':', lw=0.5)

if(show_N21_10_insert):
    inset = (ax1.inset_axes([0.45,0.05,0.3,0.23]) if(show_N28_levels) else ax1.inset_axes([0.4,0.05,0.3,0.23]))
    inset_start = N21['step_starts'][9]
    inset_stop = N21['step_starts'][10]
    inset_x = N21['time_array'][inset_start:inset_stop]
    inset_y = N21[y_params[plot_choice]][inset_start:inset_stop]
    inset_temp = N21['temperature_array'][inset_start:inset_stop]
    temp_offset = min(inset_y)
    temp_scaler = (max(inset_y)-min(inset_y)) / (max(inset_temp)-min(inset_temp))
    tplot, = inset.plot(inset_x, temp_offset+temp_scaler*(inset_temp-min(inset_temp)), 'k-', lw=0.5)
    splot, = inset.plot(inset_x, inset_y, color=N21['pen'], ls='-', lw=1)
    inset.legend([splot, tplot], ['strain','scaled\ntemperature'], loc='center right', bbox_to_anchor=(1,0.55), 
                 handlelength=1, prop={'size':8}, framealpha=1)
    inset.set_title('String N21 step 10')
    if(show_stress or show_target_freq):
        inset.text(0.035, 0.85,'(c)', transform=inset.transAxes)

print(subset, plot_set)
        
ax1.legend(loc='lower right')
ax1.set_xlabel('Time (days)')
y_label = y_labels[plot_choice]
if(make_alignment):
    y_label += ' (aligned on ' + align_string + ')'
ax1.set_ylabel(y_label)
y1_offset = 0.005 if(plot_choice == 3) else 0.002
ax1.axis([x_min-3,x_max+3,y1_min-y1_offset,y1_max+y1_offset])
if(show_stress or show_target_freq):
    ax2.set_xlabel('Time (days)')
    ax2.set_ylabel(y2_label)
    ax2.axis([x_min-3,x_max+3,y2_min-10,y2_max+10])
    ax1.text(0.035, (0.9 if(compact_plot) else 0.94),'(b)', transform=ax1.transAxes)
    ax2.text(0.035, (0.8 if(compact_plot) else 0.88),'(a)', transform=ax2.transAxes)
plt.tight_layout()  
file_part = '_aligned' if(make_alignment) else '_unaligned'
if(subset):
    save_file = save_path + 'cp_subset' + plot_set + file_part + '.' + save_format
else:
    save_file = save_path + 'cp_full_set' + file_part + '.' + save_format
print('\n',save_file)
#plt.savefig(save_file, format=save_format, dpi=1000)
plt.show()
```

```
alignment offset added to string N7: 0.000000
alignment offset added to string N20: 0.038366
alignment offset added to string N21: 0.036658
alignment offset added to string N27: 0.005773
alignment offset added to string N26: -0.043082
alignment offset added to string N28: -0.034963
alignment offset added to string N34: -0.012578
alignment offset added to string N33: -0.010736
False _720212726283433

 ./cp_full_set_aligned.eps
```

### Single-step curve fitting¶

In [4]:

```
save_results = False#True

# fitting results file format
# first row is headers for column headers:  # step, stages, A0, B, A1, X1, A2, X2, A3, X3
# for fitted function: A0 + B*t + A1*(1-np.exp(-X1*t)) + A2*(1-np.exp(-X2*t)) + A3*(1-np.exp(-X3*t))

# model functions
#  test_func0 : spring & dashpot (no K-V stages)
#  test_func1 : spring & dashpot & 1 K_V stage
#  test_func2 : spring & dashpot & 2 K_V stages
#  test_func3 : spring & dashpot & 3 K_V stages
def test_func0(t, A0, B):
    return A0 + B*t
def test_func1(t, A0, B, A1, X1):
    return A0 + B*t + A1*(1-np.exp(-X1*t))
def test_func2(t, A0, B, A1, X1, A2, X2):
    return A0 + B*t + A1*(1-np.exp(-X1*t)) + A2*(1-np.exp(-X2*t))
def test_func3(t, A0, B, A1, X1, A2, X2, A3, X3):
    return A0 + B*t + A1*(1-np.exp(-X1*t)) + A2*(1-np.exp(-X2*t)) + A3*(1-np.exp(-X3*t))
max_function = 3

N7['stage_count']  = [1,2,1,1,2,1,1,1,1,1]
N20['stage_count'] = [2,2,2,1,1,1,1,1]
N21['stage_count'] = [2,2,2,1,2,2,1,1,2,1,2,1,1,1,2,2]
N27['stage_count'] = [2,1,1,1,1,1,1,1,1,2,2,2,1,2,2,2]
N26['stage_count'] = [1,1,1,1,1]
N28['stage_count'] = [2,2,2,2,2,2,2,1,2,2,2,2,2,2,2,2,1,1]
N34['stage_count'] = [2,2,2]
N33['stage_count'] = [2,2,2,2,2,2,1,2]


for string in string_list:
#for string in ['N33']:
#for string in ['N34']:
#for string in ['N28']:
#for string in ['N26']:
#for string in ['N27']:
#for string in ['N21']:
#for string in ['N20']:
#for string in ['N7']:
    print('\nSTRING ', string, '\n')
    sdict = globals()[string]
    max_stages_req = max(sdict['stage_count'])
    if((max_stages_req <= max_stages) and (max_stages_req <= max_function)):
        # open file to save results
        if(save_results):
            filename = save_path + filename_start + str(string) + ".csv"
            op_file = open(filename,"w")
            op_string = "step, stages, A0, B"
            for i in range(max_stages):
                op_string += ", A" + str(i+1) + ", X" + str(i+1)
            op_string += "\n"
            op_file.write(op_string)

        for step in sdict['step_range']:  # step_range starts from 1
            print('Step ', step)
            start_index = sdict['step_starts'][step-1] + 1  # don't include end of previous step in the curve fitting
            stop_index = sdict['step_starts'][step]
            print('index range: ', start_index, stop_index)
            # select number of K-V stages to use
            stages = sdict['stage_count'][step-1]
            print('fitted Kelvin-Voigt stages: ', stages)
            # extract sub arrays (to make life easier)
            time_sub_array = sdict['time_array'][start_index:stop_index]
            step_strain = sdict['strain_array'][start_index:stop_index]
            #
            # curve fitting
            time_for_fitting = time_sub_array - sdict['time_array'][start_index-1]  # correct for start_index referencing one point further on
            if(string=='N20' and step==5):
                Blo = -0.001
                Bhi = 0
                Alo = 0
                Ahi = 1
            elif(string=='N33' and step==3):
                Blo = 0
                Bhi = 0.001
                Alo = -1
                Ahi = 0
            elif(step_strain[-1] > step_strain[0]): # late part of step response increasing
                Blo = 0
                Bhi = 0.001
                Alo = 0
                Ahi = 1
            else:
                Blo = -0.001
                Bhi = 0
                Alo = -1
                Ahi = 0
            Chi = 30
            # run curve fitting
            if(stages == 0):
                popt, pcov = curve_fit(test_func0, time_for_fitting, step_strain, bounds=([0,Blo],[0.2,Bhi]))
                fitted_curve = test_func0(time_for_fitting, *popt) 
            elif(stages == 1):
                popt, pcov = curve_fit(test_func1, time_for_fitting, step_strain, 
                                       bounds=([-0.01,Blo,Alo,0],[0.2,Bhi,Ahi,Chi]))
                fitted_curve = test_func1(time_for_fitting, *popt) 
            elif(stages == 2):
                popt, pcov = curve_fit(test_func2, time_for_fitting, step_strain, 
                                       bounds=([0,Blo,Alo,0,Alo,0],[0.2,Bhi,Ahi,10,Ahi,Chi]))
                fitted_curve = test_func2(time_for_fitting, *popt) 
            elif(stages == 3):
                popt, pcov = curve_fit(test_func3, time_for_fitting, step_strain, 
                                       bounds=([0,Blo,Alo,0,Alo,0,Alo,0],[0.2,Bhi,Ahi,10,Ahi,Chi,Ahi,Chi]))
                fitted_curve = test_func3(time_for_fitting, *popt) 
            print(popt)
            # re-order K-V stage coefficients, slowest first
            Ai_values = np.zeros(stages)
            Xi_values = np.zeros(stages)
            for i in range(stages):
                Ai_values[i] = popt[2+2*i]
                Xi_values[i] = popt[3+2*i]
            sort_index = np.argsort(Xi_values)
            print(Ai_values, Xi_values, sort_index)
            Ai_sorted = np.zeros(stages)
            Xi_sorted = np.zeros(stages)
            for i in range(stages):
                Ai_sorted[i] = Ai_values[np.where(sort_index==i)[0][0]]
                Xi_sorted[i] = Xi_values[np.where(sort_index==i)[0][0]]
            print(Ai_sorted, Xi_sorted)
            # write results to file
            if(save_results):
                op_string = str(step) + ", " + str(stages) + ", " + str(popt[0]) + ", " + str(popt[1])
                for i in range(stages):
                    op_string += ", " + str(Ai_sorted[i]) +  ", " + str(Xi_sorted[i])  # fitted coefficients
                op_string += "\n"
                print(op_string)
                op_file.write(op_string)
            #
            # plot data    
            plt.figure(figsize=(6,4))
            # initial data
            plt.plot(time_sub_array, step_strain, 'k-', label='strain data')
            plt.plot(time_sub_array, fitted_curve, 'b-', label='fitted curve')
            # plot component parts of fitted curve
            plt.plot(time_sub_array, [popt[0] for i in range(len(time_sub_array))], 'b:', label='offset')
            plt.plot(time_sub_array, popt[0]+popt[1]*time_for_fitting, 'g:', label='dashpot')
            if(stages>0):
                plt.plot(time_sub_array, popt[0]+popt[2]*(1-np.exp(-popt[3]*time_for_fitting)), 'r:', label='K-V stages')
            if(stages>1):
                plt.plot(time_sub_array, popt[0]+popt[4]*(1-np.exp(-popt[5]*time_for_fitting)), 'r:')
            if(stages>2):
                plt.plot(time_sub_array, popt[0]+popt[6]*(1-np.exp(-popt[7]*time_for_fitting)), 'r:')
            #
            plt.title('String ' + str(string) + ', Step ' + str(step))
            plt.xlabel('Time (days)')
            plt.ylabel('Strain')
            #plt.legend(loc='best', prop={'size':14}, framealpha=1)
            plt.tight_layout()
            savefile = save_path + 'cp_string' + str(string) + '_step' + str(step) +'_fit.' + save_format
            print(savefile)
#            plt.savefig(savefile, format=save_format, dpi=1000)
            plt.show()

        if(save_results):
            op_file.close()
    else:
        print("\n***  required number of K-V stages not supported ***\n")
```

```
STRING  N7 

Step  1
index range:  1 301
fitted Kelvin-Voigt stages:  1
[4.54435167e-03 1.22227222e-05 8.05685992e-03 4.23605412e-01]
[0.00805686] [0.42360541] [0]
[0.00805686] [0.42360541]
./cp_stringN7_step1_fit.eps
```

```
Step  2
index range:  302 538
fitted Kelvin-Voigt stages:  2
[2.12859400e-02 1.14544553e-26 6.43475875e-03 3.42534473e-01
 5.79596246e-03 3.15699858e+00]
[0.00643476 0.00579596] [0.34253447 3.15699858] [0 1]
[0.00643476 0.00579596] [0.34253447 3.15699858]
./cp_stringN7_step2_fit.eps
```

```
Step  3
index range:  539 714
fitted Kelvin-Voigt stages:  1
[3.91401255e-02 1.55710215e-04 7.45788264e-03 1.02071213e+00]
[0.00745788] [1.02071213] [0]
[0.00745788] [1.02071213]
./cp_stringN7_step3_fit.eps
```

```
Step  4
index range:  715 881
fitted Kelvin-Voigt stages:  1
[5.30366182e-02 3.77742870e-04 3.58241617e-03 1.64097861e+00]
[0.00358242] [1.64097861] [0]
[0.00358242] [1.64097861]
./cp_stringN7_step4_fit.eps
```

```
Step  5
index range:  882 1066
fitted Kelvin-Voigt stages:  2
[6.36345592e-02 3.61299257e-19 7.74616664e-03 2.77736757e-01
 3.15527463e-03 3.82754867e+00]
[0.00774617 0.00315527] [0.27773676 3.82754867] [0 1]
[0.00774617 0.00315527] [0.27773676 3.82754867]
./cp_stringN7_step5_fit.eps
```

```
Step  6
index range:  1067 1225
fitted Kelvin-Voigt stages:  1
[7.76844991e-02 4.23731710e-15 1.23713349e-02 2.91583934e-01]
[0.01237133] [0.29158393] [0]
[0.01237133] [0.29158393]
./cp_stringN7_step6_fit.eps
```

```
Step  7
index range:  1226 1387
fitted Kelvin-Voigt stages:  1
[9.21966198e-02 1.08145161e-04 1.31769055e-02 5.61924090e-01]
[0.01317691] [0.56192409] [0]
[0.01317691] [0.56192409]
./cp_stringN7_step7_fit.eps
```

```
Step  8
index range:  1388 1569
fitted Kelvin-Voigt stages:  1
[1.08914213e-01 4.65233511e-04 1.33460527e-02 6.64460282e-01]
[0.01334605] [0.66446028] [0]
[0.01334605] [0.66446028]
./cp_stringN7_step8_fit.eps
```

```
Step  9
index range:  1570 1706
fitted Kelvin-Voigt stages:  1
[0.12915066 0.001      0.01329554 0.64105703]
[0.01329554] [0.64105703] [0]
[0.01329554] [0.64105703]
./cp_stringN7_step9_fit.eps
```

```
Step  10
index range:  1707 1843
fitted Kelvin-Voigt stages:  1
[0.15317584 0.00097476 0.02151137 0.43290033]
[0.02151137] [0.43290033] [0]
[0.02151137] [0.43290033]
./cp_stringN7_step10_fit.eps
```

```
STRING  N20 

Step  1
index range:  1 826
fitted Kelvin-Voigt stages:  2
[4.84702520e-02 2.32303476e-04 8.75172089e-03 9.98834517e-01
 7.69832831e-03 2.19997069e+01]
[0.00875172 0.00769833] [ 0.99883452 21.99970695] [0 1]
[0.00875172 0.00769833] [ 0.99883452 21.99970695]
./cp_stringN20_step1_fit.eps
```

```
Step  2
index range:  827 1518
fitted Kelvin-Voigt stages:  2
[7.07603398e-02 4.10498647e-26 1.40636764e-02 2.23129839e-01
 3.26312920e-03 2.94452998e+00]
[0.01406368 0.00326313] [0.22312984 2.94452998] [0 1]
[0.01406368 0.00326313] [0.22312984 2.94452998]
./cp_stringN20_step2_fit.eps
```

```
Step  3
index range:  1519 2067
fitted Kelvin-Voigt stages:  2
[ 8.23219253e-02 -1.29739723e-04 -1.07367647e-03  9.40099765e-01
 -3.80687033e-04  2.53452867e+01]
[-0.00107368 -0.00038069] [ 0.94009977 25.34528668] [0 1]
[-0.00107368 -0.00038069] [ 0.94009977 25.34528668]
./cp_stringN20_step3_fit.eps
```

```
Step  4
index range:  2068 2686
fitted Kelvin-Voigt stages:  1
[ 7.67204012e-02 -3.22353773e-04 -6.69673735e-04  4.02156535e+00]
[-0.00066967] [4.02156535] [0]
[-0.00066967] [4.02156535]
./cp_stringN20_step4_fit.eps
```

```
Step  5
index range:  2687 3272
fitted Kelvin-Voigt stages:  1
[ 7.69649090e-02 -1.09356991e-04  5.72059887e-04  3.95968069e+00]
[0.00057206] [3.95968069] [0]
[0.00057206] [3.95968069]
./cp_stringN20_step5_fit.eps
```

```
Step  6
index range:  3273 3833
fitted Kelvin-Voigt stages:  1
[8.03539923e-02 1.02550336e-04 5.20166356e-04 1.66639381e+00]
[0.00052017] [1.66639381] [0]
[0.00052017] [1.66639381]
./cp_stringN20_step6_fit.eps
```

```
Step  7
index range:  3834 4417
fitted Kelvin-Voigt stages:  1
[ 7.85186758e-02 -2.13464599e-04 -5.16981636e-04  1.13214906e+01]
[-0.00051698] [11.32149059] [0]
[-0.00051698] [11.32149059]
./cp_stringN20_step7_fit.eps
```

```
Step  8
index range:  4418 5001
fitted Kelvin-Voigt stages:  1
[ 7.31969562e-02 -1.97342331e-04 -6.17344009e-04  1.12559847e+00]
[-0.00061734] [1.12559847] [0]
[-0.00061734] [1.12559847]
./cp_stringN20_step8_fit.eps
```

```
STRING  N21 

Step  1
index range:  1 3488
fitted Kelvin-Voigt stages:  2
[4.94377172e-02 2.73476508e-04 8.61327732e-03 5.37060098e-01
 7.68234727e-03 1.67123428e+01]
[0.00861328 0.00768235] [ 0.5370601  16.71234285] [0 1]
[0.00861328 0.00768235] [ 0.5370601  16.71234285]
./cp_stringN21_step1_fit.eps
```

```
Step  2
index range:  3489 5180
fitted Kelvin-Voigt stages:  2
[ 6.58273591e-02 -4.40577961e-17 -5.72950282e-04  6.17419572e-01
 -5.88050686e-04  2.23929897e+01]
[-0.00057295 -0.00058805] [ 0.61741957 22.39298971] [0 1]
[-0.00057295 -0.00058805] [ 0.61741957 22.39298971]
./cp_stringN21_step2_fit.eps
```

```
Step  3
index range:  5181 7323
fitted Kelvin-Voigt stages:  2
[ 6.18402456e-02 -2.70081835e-16 -1.10375596e-03  6.35009354e-01
 -4.32206048e-04  1.04465161e+01]
[-0.00110376 -0.00043221] [ 0.63500935 10.44651607] [0 1]
[-0.00110376 -0.00043221] [ 0.63500935 10.44651607]
./cp_stringN21_step3_fit.eps
```

```
Step  4
index range:  7324 8483
fitted Kelvin-Voigt stages:  1
[6.30958955e-02 5.34861127e-16 7.13644830e-04 1.33944500e+00]
[0.00071364] [1.339445] [0]
[0.00071364] [1.339445]
./cp_stringN21_step4_fit.eps
```

```
Step  5
index range:  8484 11147
fitted Kelvin-Voigt stages:  2
[6.68880707e-02 7.22509008e-05 5.35519199e-04 8.79129857e-01
 3.66333839e-04 2.05316766e+01]
[0.00053552 0.00036633] [ 0.87912986 20.53167656] [0 1]
[0.00053552 0.00036633] [ 0.87912986 20.53167656]
./cp_stringN21_step5_fit.eps
```

```
Step  6
index range:  11148 15107
fitted Kelvin-Voigt stages:  2
[7.15308334e-02 1.48959554e-04 3.18320615e-03 3.46675918e-01
 4.73053835e-04 1.18146296e+01]
[0.00318321 0.00047305] [ 0.34667592 11.81462958] [0 1]
[0.00318321 0.00047305] [ 0.34667592 11.81462958]
./cp_stringN21_step6_fit.eps
```

```
Step  7
index range:  15108 16723
fitted Kelvin-Voigt stages:  1
[ 7.43790779e-02 -1.49161566e-05 -5.84118675e-04  2.71747663e+00]
[-0.00058412] [2.71747663] [0]
[-0.00058412] [2.71747663]
./cp_stringN21_step7_fit.eps
```

```
Step  8
index range:  16724 18581
fitted Kelvin-Voigt stages:  1
[ 7.04221948e-02 -7.81641552e-05 -6.70587816e-04  1.50297897e+00]
[-0.00067059] [1.50297897] [0]
[-0.00067059] [1.50297897]
./cp_stringN21_step8_fit.eps
```

```
Step  9
index range:  18582 20386
fitted Kelvin-Voigt stages:  2
[7.22289177e-02 3.86616899e-34 9.25126932e-04 3.41772378e-01
 3.49414439e-04 1.33176080e+01]
[0.00092513 0.00034941] [ 0.34177238 13.31760804] [0 1]
[0.00092513 0.00034941] [ 0.34177238 13.31760804]
./cp_stringN21_step9_fit.eps
```

```
Step  10
index range:  20387 22127
fitted Kelvin-Voigt stages:  1
[7.27820129e-02 3.90049892e-21 6.81354286e-03 4.13887911e-01]
[0.00681354] [0.41388791] [0]
[0.00681354] [0.41388791]
./cp_stringN21_step10_fit.eps
```

```
Step  11
index range:  22128 24020
fitted Kelvin-Voigt stages:  2
[8.20652837e-02 6.61789889e-05 1.02350058e-03 6.48236295e-01
 2.89560291e-04 2.90943750e+01]
[0.0010235  0.00028956] [ 0.6482363  29.09437503] [0 1]
[0.0010235  0.00028956] [ 0.6482363  29.09437503]
./cp_stringN21_step11_fit.eps
```

```
Step  12
index range:  24021 25616
fitted Kelvin-Voigt stages:  1
[ 8.07149289e-02 -2.83803761e-06 -4.30240003e-04  4.35830648e+00]
[-0.00043024] [4.35830648] [0]
[-0.00043024] [4.35830648]
./cp_stringN21_step12_fit.eps
```

```
Step  13
index range:  25617 27206
fitted Kelvin-Voigt stages:  1
[ 7.69159263e-02 -1.22292546e-04 -4.60567533e-04  2.61157350e+00]
[-0.00046057] [2.6115735] [0]
[-0.00046057] [2.6115735]
./cp_stringN21_step13_fit.eps
```

```
Step  14
index range:  27207 28902
fitted Kelvin-Voigt stages:  1
[7.88371460e-02 2.00836045e-05 7.45205277e-04 1.20773469e+00]
[0.00074521] [1.20773469] [0]
[0.00074521] [1.20773469]
./cp_stringN21_step14_fit.eps
```

```
Step  15
index range:  28903 30613
fitted Kelvin-Voigt stages:  2
[8.25853574e-02 1.12973887e-04 4.82558179e-04 1.11757354e+00
 3.54481492e-04 9.14492766e+00]
[0.00048256 0.00035448] [1.11757354 9.14492766] [0 1]
[0.00048256 0.00035448] [1.11757354 9.14492766]
./cp_stringN21_step15_fit.eps
```

```
Step  16
index range:  30614 32666
fitted Kelvin-Voigt stages:  2
[8.63079672e-02 7.00512077e-04 6.88610353e-04 1.43709582e+00
 8.05353118e-05 2.99998092e+01]
[6.88610353e-04 8.05353118e-05] [ 1.43709582 29.99980919] [0 1]
[6.88610353e-04 8.05353118e-05] [ 1.43709582 29.99980919]
./cp_stringN21_step16_fit.eps
```

```
STRING  N27 

Step  1
index range:  1 3107
fitted Kelvin-Voigt stages:  2
[8.08614195e-02 2.64559964e-16 1.16252863e-02 2.40896025e-01
 8.42366627e-03 2.06702341e+00]
[0.01162529 0.00842367] [0.24089603 2.06702341] [0 1]
[0.01162529 0.00842367] [0.24089603 2.06702341]
./cp_stringN27_step1_fit.eps
```

```
Step  2
index range:  3108 6528
fitted Kelvin-Voigt stages:  1
[0.10405723 0.00039118 0.01109832 0.2054424 ]
[0.01109832] [0.2054424] [0]
[0.01109832] [0.2054424]
./cp_stringN27_step2_fit.eps
```

```
Step  3
index range:  6529 8161
fitted Kelvin-Voigt stages:  1
[ 1.15298429e-01 -3.62899951e-05 -4.55620814e-04  2.03421356e+00]
[-0.00045562] [2.03421356] [0]
[-0.00045562] [2.03421356]
./cp_stringN27_step3_fit.eps
```

```
Step  4
index range:  8162 9946
fitted Kelvin-Voigt stages:  1
[ 1.10966454e-01 -2.00776228e-05 -1.10410895e-03  9.87195706e-01]
[-0.00110411] [0.98719571] [0]
[-0.00110411] [0.98719571]
./cp_stringN27_step4_fit.eps
```

```
Step  5
index range:  9947 11274
fitted Kelvin-Voigt stages:  1
[1.13425521e-01 1.60401527e-05 5.54399596e-04 2.04455073e+00]
[0.0005544] [2.04455073] [0]
[0.0005544] [2.04455073]
./cp_stringN27_step5_fit.eps
```

```
Step  6
index range:  11275 13351
fitted Kelvin-Voigt stages:  1
[1.17391239e-01 3.07901528e-04 5.01184088e-04 5.52624986e+00]
[0.00050118] [5.52624986] [0]
[0.00050118] [5.52624986]
./cp_stringN27_step6_fit.eps
```

```
Step  7
index range:  13352 14774
fitted Kelvin-Voigt stages:  1
[ 1.16637271e-01 -2.23664925e-05 -3.97971819e-04  1.47384902e+01]
[-0.00039797] [14.73849018] [0]
[-0.00039797] [14.73849018]
./cp_stringN27_step7_fit.eps
```

```
Step  8
index range:  14775 16766
fitted Kelvin-Voigt stages:  1
[ 1.12414914e-01 -6.08601808e-05 -6.12121474e-04  9.56108051e-01]
[-0.00061212] [0.95610805] [0]
[-0.00061212] [0.95610805]
./cp_stringN27_step8_fit.eps
```

```
Step  9
index range:  16767 19005
fitted Kelvin-Voigt stages:  1
[ 1.07763134e-01 -5.19372636e-05 -1.14123445e-03  6.18046229e-01]
[-0.00114123] [0.61804623] [0]
[-0.00114123] [0.61804623]
./cp_stringN27_step9_fit.eps
```

```
Step  10
index range:  19006 20948
fitted Kelvin-Voigt stages:  2
[ 1.03091518e-01 -1.70057348e-04 -1.04721662e-03  7.14759049e-01
 -2.29997877e-04  2.32400176e+01]
[-0.00104722 -0.00023   ] [ 0.71475905 23.24001762] [0 1]
[-0.00104722 -0.00023   ] [ 0.71475905 23.24001762]
./cp_stringN27_step10_fit.eps
```

```
Step  11
index range:  20949 23220
fitted Kelvin-Voigt stages:  2
[ 9.77586722e-02 -1.67305209e-04 -1.08563325e-03  6.26447261e-01
 -3.39705116e-04  1.22399685e+01]
[-0.00108563 -0.00033971] [ 0.62644726 12.2399685 ] [0 1]
[-0.00108563 -0.00033971] [ 0.62644726 12.2399685 ]
./cp_stringN27_step11_fit.eps
```

```
Step  12
index range:  23221 24943
fitted Kelvin-Voigt stages:  2
[1.00616341e-01 3.36286839e-05 1.23152581e-03 1.05201429e+00
 7.86327754e-04 2.63495366e+01]
[0.00123153 0.00078633] [ 1.05201429 26.3495366 ] [0 1]
[0.00123153 0.00078633] [ 1.05201429 26.3495366 ]
./cp_stringN27_step12_fit.eps
```

```
Step  13
index range:  24944 26362
fitted Kelvin-Voigt stages:  1
[1.06307642e-01 1.74295875e-04 7.57892276e-04 1.66958709e+00]
[0.00075789] [1.66958709] [0]
[0.00075789] [1.66958709]
./cp_stringN27_step13_fit.eps
```

```
Step  14
index range:  26363 27813
fitted Kelvin-Voigt stages:  2
[1.11379355e-01 1.51011356e-04 1.31095104e-03 1.31460851e+00
 3.28702422e-04 1.94482829e+01]
[0.00131095 0.0003287 ] [ 1.31460851 19.44828294] [0 1]
[0.00131095 0.0003287 ] [ 1.31460851 19.44828294]
./cp_stringN27_step14_fit.eps
```

```
Step  15
index range:  27814 30699
fitted Kelvin-Voigt stages:  2
[1.17291680e-01 1.27133929e-05 5.07844994e-03 2.85225586e-01
 4.26258987e-04 1.48565364e+01]
[0.00507845 0.00042626] [ 0.28522559 14.85653637] [0 1]
[0.00507845 0.00042626] [ 0.28522559 14.85653637]
./cp_stringN27_step15_fit.eps
```

```
Step  16
index range:  30700 35535
fitted Kelvin-Voigt stages:  2
[ 1.19512711e-01 -2.09606948e-05 -4.25543926e-04  5.84269948e-01
 -5.25803420e-04  2.33252766e+01]
[-0.00042554 -0.0005258 ] [ 0.58426995 23.32527664] [0 1]
[-0.00042554 -0.0005258 ] [ 0.58426995 23.32527664]
./cp_stringN27_step16_fit.eps
```

```
STRING  N26 

Step  1
index range:  1 3752
fitted Kelvin-Voigt stages:  1
[1.25858098e-01 3.19690421e-04 1.88157254e-02 5.54078903e-01]
[0.01881573] [0.5540789] [0]
[0.01881573] [0.5540789]
./cp_stringN26_step1_fit.eps
```

```
Step  2
index range:  3753 5513
fitted Kelvin-Voigt stages:  1
[ 1.44716439e-01 -1.50998745e-19 -1.24533942e-03  3.41640311e-01]
[-0.00124534] [0.34164031] [0]
[-0.00124534] [0.34164031]
./cp_stringN26_step2_fit.eps
```

```
Step  3
index range:  5514 8346
fitted Kelvin-Voigt stages:  1
[1.47423427e-01 2.46049917e-04 5.31040981e-04 5.68626480e+00]
[0.00053104] [5.6862648] [0]
[0.00053104] [5.6862648]
./cp_stringN26_step3_fit.eps
```

```
Step  4
index range:  8347 11450
fitted Kelvin-Voigt stages:  1
[1.54932841e-01 4.87015075e-22 2.06611422e-02 1.89752663e-01]
[0.02066114] [0.18975266] [0]
[0.02066114] [0.18975266]
./cp_stringN26_step4_fit.eps
```

```
Step  5
index range:  11451 11498
fitted Kelvin-Voigt stages:  1
[1.75888218e-01 3.88182046e-04 2.02933340e-02 4.33228800e-01]
[0.02029333] [0.4332288] [0]
[0.02029333] [0.4332288]
./cp_stringN26_step5_fit.eps
```

```
STRING  N28 

Step  1
index range:  1 3782
fitted Kelvin-Voigt stages:  2
[1.14151144e-02 1.40489375e-15 1.04833656e-02 1.39084903e-01
 5.21939784e-03 1.18884052e+00]
[0.01048337 0.0052194 ] [0.1390849  1.18884052] [0 1]
[0.01048337 0.0052194 ] [0.1390849  1.18884052]
./cp_stringN28_step1_fit.eps
```

```
Step  2
index range:  3783 6618
fitted Kelvin-Voigt stages:  2
[3.46290973e-02 2.93015521e-04 7.12932937e-03 8.46198152e-01
 1.10858253e-02 6.91239144e+00]
[0.00712933 0.01108583] [0.84619815 6.91239144] [0 1]
[0.00712933 0.01108583] [0.84619815 6.91239144]
./cp_stringN28_step2_fit.eps
```

```
Step  3
index range:  6619 8891
fitted Kelvin-Voigt stages:  2
[6.26809203e-02 2.45399604e-04 1.05378262e-02 7.04332546e-01
 1.45335843e-03 1.44270205e+01]
[0.01053783 0.00145336] [ 0.70433255 14.42702051] [0 1]
[0.01053783 0.00145336] [ 0.70433255 14.42702051]
./cp_stringN28_step3_fit.eps
```

```
Step  4
index range:  8892 10892
fitted Kelvin-Voigt stages:  2
[ 7.06535367e-02 -6.04827188e-05 -1.00538407e-03  7.45126255e-01
 -1.60432654e-03  6.44023290e+00]
[-0.00100538 -0.00160433] [0.74512625 6.4402329 ] [0 1]
[-0.00100538 -0.00160433] [0.74512625 6.4402329 ]
./cp_stringN28_step4_fit.eps
```

```
Step  5
index range:  10893 12666
fitted Kelvin-Voigt stages:  2
[7.34907003e-02 5.78179460e-17 3.28364761e-03 3.42071601e-01
 1.16190459e-03 9.67885516e+00]
[0.00328365 0.0011619 ] [0.3420716  9.67885516] [0 1]
[0.00328365 0.0011619 ] [0.3420716  9.67885516]
./cp_stringN28_step5_fit.eps
```

```
Step  6
index range:  12667 14624
fitted Kelvin-Voigt stages:  2
[8.36053447e-02 2.94741773e-04 5.77817459e-03 5.32024971e-01
 1.52796882e-03 9.97460606e+00]
[0.00577817 0.00152797] [0.53202497 9.97460606] [0 1]
[0.00577817 0.00152797] [0.53202497 9.97460606]
./cp_stringN28_step6_fit.eps
```

```
Step  7
index range:  14625 16653
fitted Kelvin-Voigt stages:  2
[9.82749108e-02 3.06548842e-15 4.54585786e-03 2.74431639e-01
 5.70837256e-03 2.76387477e+00]
[0.00454586 0.00570837] [0.27443164 2.76387477] [0 1]
[0.00454586 0.00570837] [0.27443164 2.76387477]
./cp_stringN28_step7_fit.eps
```

```
Step  8
index range:  16654 18652
fitted Kelvin-Voigt stages:  1
[ 9.72542335e-02 -1.39426194e-04 -2.71511678e-03  3.59022122e+00]
[-0.00271512] [3.59022122] [0]
[-0.00271512] [3.59022122]
./cp_stringN28_step8_fit.eps
```

```
Step  9
index range:  18653 22934
fitted Kelvin-Voigt stages:  2
[1.03664225e-01 9.32627719e-05 3.16562726e-03 2.08712620e-01
 2.49520987e-03 7.15216566e+00]
[0.00316563 0.00249521] [0.20871262 7.15216566] [0 1]
[0.00316563 0.00249521] [0.20871262 7.15216566]
./cp_stringN28_step9_fit.eps
```

```
Step  10
index range:  22935 24957
fitted Kelvin-Voigt stages:  2
[1.14938282e-01 4.35614219e-24 7.86148540e-03 2.93665023e-01
 6.99794207e-04 1.95089685e+01]
[0.00786149 0.00069979] [ 0.29366502 19.50896848] [0 1]
[0.00786149 0.00069979] [ 0.29366502 19.50896848]
./cp_stringN28_step10_fit.eps
```

```
Step  11
index range:  24958 28385
fitted Kelvin-Voigt stages:  2
[1.26808324e-01 4.15427061e-21 1.18757978e-02 1.86945698e-01
 3.38497983e-03 1.47946032e+00]
[0.0118758  0.00338498] [0.1869457  1.47946032] [0 1]
[0.0118758  0.00338498] [0.1869457  1.47946032]
./cp_stringN28_step11_fit.eps
```

```
Step  12
index range:  28386 30115
fitted Kelvin-Voigt stages:  2
[ 1.37494037e-01 -2.58235810e-06 -5.39034319e-04  6.26566564e-01
 -3.74448660e-04  7.78531158e+00]
[-0.00053903 -0.00037445] [0.62656656 7.78531158] [0 1]
[-0.00053903 -0.00037445] [0.62656656 7.78531158]
./cp_stringN28_step12_fit.eps
```

```
Step  13
index range:  30116 32426
fitted Kelvin-Voigt stages:  2
[ 1.33095966e-01 -4.41750086e-05 -9.18279703e-04  5.43307840e-01
 -5.39336678e-04  7.61774476e+00]
[-0.00091828 -0.00053934] [0.54330784 7.61774476] [0 1]
[-0.00091828 -0.00053934] [0.54330784 7.61774476]
./cp_stringN28_step13_fit.eps
```

```
Step  14
index range:  32427 33582
fitted Kelvin-Voigt stages:  2
[1.34801707e-01 6.70645593e-05 4.54179927e-04 8.90755858e-01
 3.40189804e-04 1.50252201e+01]
[0.00045418 0.00034019] [ 0.89075586 15.02522006] [0 1]
[0.00045418 0.00034019] [ 0.89075586 15.02522006]
./cp_stringN28_step14_fit.eps
```

```
Step  15
index range:  33583 36170
fitted Kelvin-Voigt stages:  2
[1.39064960e-01 8.37962258e-05 1.94665037e-03 2.77625209e-01
 3.24119129e-04 1.29743089e+01]
[0.00194665 0.00032412] [ 0.27762521 12.97430891] [0 1]
[0.00194665 0.00032412] [ 0.27762521 12.97430891]
./cp_stringN28_step15_fit.eps
```

```
Step  16
index range:  36171 41317
fitted Kelvin-Voigt stages:  2
[1.45157585e-01 8.15030967e-23 1.20264702e-02 7.27898522e-02
 2.32893984e-03 6.74126393e-01]
[0.01202647 0.00232894] [0.07278985 0.67412639] [0 1]
[0.01202647 0.00232894] [0.07278985 0.67412639]
./cp_stringN28_step16_fit.eps
```

```
Step  17
index range:  41318 43033
fitted Kelvin-Voigt stages:  1
[0.15919762 0.00050659 0.00988683 0.43325356]
[0.00988683] [0.43325356] [0]
[0.00988683] [0.43325356]
./cp_stringN28_step17_fit.eps
```

```
Step  18
index range:  43034 43186
fitted Kelvin-Voigt stages:  1
[0.17422668 0.00099982 0.01652613 0.92613113]
[0.01652613] [0.92613113] [0]
[0.01652613] [0.92613113]
./cp_stringN28_step18_fit.eps
```

```
STRING  N34 

Step  1
index range:  1 12022
fitted Kelvin-Voigt stages:  2
[9.88190980e-02 8.64375341e-05 9.97066534e-03 2.56312710e-01
 6.06087967e-03 2.22320567e+00]
[0.00997067 0.00606088] [0.25631271 2.22320567] [0 1]
[0.00997067 0.00606088] [0.25631271 2.22320567]
./cp_stringN34_step1_fit.eps
```

```
Step  2
index range:  12023 17093
fitted Kelvin-Voigt stages:  2
[1.21540623e-01 5.76195051e-04 1.37731381e-03 1.29295591e+00
 5.06469465e-04 2.83492860e+01]
[0.00137731 0.00050647] [ 1.29295591 28.34928602] [0 1]
[0.00137731 0.00050647] [ 1.29295591 28.34928602]
./cp_stringN34_step2_fit.eps
```

```
Step  3
index range:  17094 18534
fitted Kelvin-Voigt stages:  2
[1.36722330e-01 1.00000000e-03 2.33681067e-02 2.25881249e-01
 7.15929504e-04 2.99999941e+01]
[0.02336811 0.00071593] [ 0.22588125 29.9999941 ] [0 1]
[0.02336811 0.00071593] [ 0.22588125 29.9999941 ]
./cp_stringN34_step3_fit.eps
```

```
STRING  N33 

Step  1
index range:  1 3110
fitted Kelvin-Voigt stages:  2
[9.71146655e-02 2.87234910e-04 8.99321802e-03 1.00783105e+00
 7.41005019e-03 2.06767353e+01]
[0.00899322 0.00741005] [ 1.00783105 20.6767353 ] [0 1]
[0.00899322 0.00741005] [ 1.00783105 20.6767353 ]
./cp_stringN33_step1_fit.eps
```

```
Step  2
index range:  3111 5414
fitted Kelvin-Voigt stages:  2
[1.21374710e-01 1.00000000e-03 5.49248364e-03 3.62689463e-01
 1.13402111e-03 8.31068273e+00]
[0.00549248 0.00113402] [0.36268946 8.31068273] [0 1]
[0.00549248 0.00113402] [0.36268946 8.31068273]
./cp_stringN33_step2_fit.eps
```

```
Step  3
index range:  5415 7420
fitted Kelvin-Voigt stages:  2
[ 1.32812465e-01  5.78770782e-05 -5.10070670e-04  1.19622282e+00
 -4.68798166e-04  1.84118304e+01]
[-0.00051007 -0.0004688 ] [ 1.19622282 18.41183042] [0 1]
[-0.00051007 -0.0004688 ] [ 1.19622282 18.41183042]
./cp_stringN33_step3_fit.eps
```

```
Step  4
index range:  7421 9433
fitted Kelvin-Voigt stages:  2
[ 1.28806388e-01 -4.09616262e-05 -6.21087851e-04  7.45578883e-01
 -6.25316587e-04  5.55872092e+00]
[-0.00062109 -0.00062532] [0.74557888 5.55872092] [0 1]
[-0.00062109 -0.00062532] [0.74557888 5.55872092]
./cp_stringN33_step4_fit.eps
```

```
Step  5
index range:  9434 11430
fitted Kelvin-Voigt stages:  2
[1.30605276e-01 1.47523457e-20 1.27717532e-03 4.60008085e-01
 3.84980546e-04 8.94166310e+00]
[0.00127718 0.00038498] [0.46000809 8.9416631 ] [0 1]
[0.00127718 0.00038498] [0.46000809 8.9416631 ]
./cp_stringN33_step5_fit.eps
```

```
Step  6
index range:  11431 15186
fitted Kelvin-Voigt stages:  2
[1.35553280e-01 3.34378962e-04 1.01349250e-03 6.01164426e-01
 4.46688715e-04 6.61999463e+00]
[0.00101349 0.00044669] [0.60116443 6.61999463] [0 1]
[0.00101349 0.00044669] [0.60116443 6.61999463]
./cp_stringN33_step6_fit.eps
```

```
Step  7
index range:  15187 16909
fitted Kelvin-Voigt stages:  1
[1.44138517e-01 2.50702542e-19 1.55507375e-02 3.33243569e-01]
[0.01555074] [0.33324357] [0]
[0.01555074] [0.33324357]
./cp_stringN33_step7_fit.eps
```

```
Step  8
index range:  16910 17236
fitted Kelvin-Voigt stages:  2
[1.60099694e-01 4.81164623e-13 1.57876518e-02 7.81615104e-01
 1.23404157e-03 8.89022894e+00]
[0.01578765 0.00123404] [0.7816151  8.89022894] [0 1]
[0.01578765 0.00123404] [0.7816151  8.89022894]
./cp_stringN33_step8_fit.eps
```

## Elastic strain study¶

In [5]:

```
#string_list = ['N7','N20','N21','N27','N26','N28','N34','N33']

def test_function_KG(stress, K, G):
    return elastic_strain_from_stress(stress/1000.0, K, G*1000.0) # scale G for GPa

def test_function_AK(stress, A, K):
    return A + elastic_strain_from_stress(stress/1000.0, K, Et_coeff_b*1000.0) # scale Et_coeff_b for GPa

def test_function_AKG(stress, A, K, G):
    return A + elastic_strain_from_stress(stress/1000.0, K, G*1000.0) # scale Et_coeff_b for GPa

get_fitted_components(save_path+filename_start, string_list)
    # reads fitted coefficients into sdict['fitted_values']
    # data_array format: row=step(0..n); columns: stages A0 B A1 X1 A2 X2 A3 X3
    # for A0 + B*t + A1*(1-np.exp(-X1*t)) + A2*(1-np.exp(-X2*t)) + A3*(1-np.exp(-X3*t))
    # creates sdict['fitted_steps'] arrays containing the elastic strain step at the start of each step response
for string in string_list:
    sdict = globals()[string]
    # total 'elastic' strain
    sdict['fitted_el_strain'] = np.array([np.sum(sdict['fitted_steps'][:i+1]) for i in range(sdict['test_steps'])])

ref_string = 'N7'
print('\n--> ref_string =', ref_string, '\n')
sdict = globals()[ref_string]
# calculate reference level to align at 425, 429 Hz, ~198 MPa, using selected string as reference
print(sdict['freq'])
ref_points = np.array([((424 < sdict['freq'][i]) and (sdict['freq'][i] < 430)) for i in range(len(sdict['freq']))])
if(ref_string == 'N21'):
    ref_points[9] = 0
ref_level = sum(ref_points * sdict['fitted_el_strain']) / sum(ref_points)
print(ref_level,'\n')

fig=plt.figure(figsize=fig_size_1)
ax1=fig.add_subplot(111)

x_range = np.arange(0,300,5)

# total elastic strain versus stress
# calculate alignment offsets for selected reference string
for string in string_list:
    sdict = globals()[string]
    aln_index = 6 if(string == 'N7') else 10 if(string == 'N28') else 0
    sdict['el_aln_offset'] = ref_level - sdict['fitted_el_strain'][aln_index]
# fit test function to fitted values for strings N7 and N28
for string in ['N7','N28']:
    sdict = globals()[string]
    # fitting to strain = ln(1 + stress.G/K) / G
    popt_KG, pcov_KG = curve_fit(test_function_KG, sdict['stress_n'][:-1], sdict['fitted_el_strain'][:-1], 
                                 p0=[2.5,0.035], bounds=(0,np.inf))
    r_sqr = r_squared(sdict['fitted_el_strain'][:-1], test_function_KG(sdict['stress_n'][:-1], *popt_KG))
    print('  fit for string', string, ':', popt_KG, 'r^2 =', r_sqr)
    print('  fitted curve equivalent to E_fit (GPa) = %0.3f + %0.5f x stress (MPa)' % (popt_KG[0], popt_KG[1]))
    print('  expected curve from expression for E_T = %0.3f + %0.5f x stress\n' % (sdict['E0_est'], Et_coeff_b))
    fitted_curve = elastic_strain_from_stress(x_range/1000.0, popt_KG[0], popt_KG[1]*1000.0) # scale for GPa
    #ax1.plot(x_range, fitted_curve + sdict['el_aln_offset'], color=sdict['pen'], ls='--')
    # fitting to strain = A + ln(1 + stress.G/K) / G with G fixed to expected value of 0.0353
    popt_AK, pcov_AK = curve_fit(test_function_AK, sdict['stress_n'][:-1], sdict['fitted_el_strain'][:-1], 
                                 p0=[0.01,2.5], bounds=(-10,10))
    r_sqr = r_squared(sdict['fitted_el_strain'][:-1], test_function_AK(sdict['stress_n'][:-1], *popt_AK))
    print('  fit for string', string, ':', popt_AK, 'r^2 =', r_sqr)
    print('  fitted curve equivalent to E_fit (GPa) = %0.3f + %0.5f x stress (MPa), with offset %0.5f' 
          % (popt_AK[1], Et_coeff_b, popt_AK[0]))
    print('  expected curve from expression for E_T = %0.3f + %0.5f x stress\n' % (sdict['E0_est'], Et_coeff_b))
    fitted_curve = popt_AK[0] + elastic_strain_from_stress(x_range/1000.0, popt_AK[1], Et_coeff_b*1000.0) # scale for GPa
    #ax1.plot(x_range, fitted_curve + sdict['el_aln_offset'], color=sdict['pen'], ls='--')
    # fitting to strain = A + ln(1 + stress.G/K) / G with free fit for all parameters (gives best fits)
    popt_AKG, pcov_AKG = curve_fit(test_function_AKG, sdict['stress_n'][:-1], sdict['fitted_el_strain'][:-1], 
                                   p0=[0.01,2.5, 0.035], bounds=(-10,10))
    r_sqr = r_squared(sdict['fitted_el_strain'][:-1], test_function_AKG(sdict['stress_n'][:-1], *popt_AKG))
    print('  fit for string', string, ':', popt_AKG, 'r^2 =', r_sqr)
    print('  fitted curve equivalent to E_fit (GPa) = %0.3f + %0.5f x stress (MPa), with offset %0.5f' 
          % (popt_AKG[1], popt_AKG[2], popt_AKG[0]))
    print('  expected curve from expression for E_T = %0.3f + %0.5f x stress\n' % (sdict['E0_est'], Et_coeff_b))
    fitted_curve = popt_AKG[0] + elastic_strain_from_stress(x_range/1000.0, popt_AKG[1], popt_AKG[2]*1000.0) # scale for GPa
    ax1.plot(x_range, fitted_curve + sdict['el_aln_offset'], color=sdict['pen'], ls='--')

# plot data
for string in string_list:
    sdict = globals()[string]
    ax1.plot(sdict['stress_n'], sdict['fitted_el_strain'] + sdict['el_aln_offset'], color=sdict['pen'], 
             ls=sdict['style'], marker='o', ms=3, mfc=sdict['pen'], label=sdict['leg'])
ax1.legend(loc='lower right', ncol = 2, framealpha = 1)
ax1.set_xlabel('Stress (MPa)')
ax1.set_ylabel('Total elastic strain $\Sigma A_{el}$ (alligned on '+ref_string+')')

fig.tight_layout()
savefile = save_path + "elastic_strain_study_" + ref_string + "." + save_format
print("\n%s" % (savefile))
#fig.savefig(savefile, format=save_format, dpi=1000)
plt.show()
```

```
--> ref_string = N7 

[173.85 235.   283.24 324.38 360.87 393.99 424.53 453.02 479.82 505.2 ]
0.03619404124482188 

  fit for string N7 : [4.07936568 0.01576473] r^2 = 0.9909127683267457
  fitted curve equivalent to E_fit (GPa) = 4.079 + 0.01576 x stress (MPa)
  expected curve from expression for E_T = 2.515 + 0.03528 x stress

  fit for string N7 : [-0.01079913  1.64350982] r^2 = 0.9991895661862127
  fitted curve equivalent to E_fit (GPa) = 1.644 + 0.03528 x stress (MPa), with offset -0.01080
  expected curve from expression for E_T = 2.515 + 0.03528 x stress

  fit for string N7 : [-0.00829729  2.04770194  0.03119474] r^2 = 0.9996460093863152
  fitted curve equivalent to E_fit (GPa) = 2.048 + 0.03119 x stress (MPa), with offset -0.00830
  expected curve from expression for E_T = 2.515 + 0.03528 x stress

  fit for string N28 : [2.26058433 0.02888098] r^2 = 0.9903888409910825
  fitted curve equivalent to E_fit (GPa) = 2.261 + 0.02888 x stress (MPa)
  expected curve from expression for E_T = 2.887 + 0.03528 x stress

  fit for string N28 : [-0.0036649   1.63847197] r^2 = 0.9888391747733877
  fitted curve equivalent to E_fit (GPa) = 1.638 + 0.03528 x stress (MPa), with offset -0.00366
  expected curve from expression for E_T = 2.887 + 0.03528 x stress

  fit for string N28 : [1.03313222e-03 2.45632840e+00 2.72282961e-02] r^2 = 0.9904731311491359
  fitted curve equivalent to E_fit (GPa) = 2.456 + 0.02723 x stress (MPa), with offset 0.00103
  expected curve from expression for E_T = 2.887 + 0.03528 x stress


./elastic_strain_study_N7.eps
```

## Recoverable creep study¶

In [6]:

```
#string_list = ['N7','N20','N21','N27','N26','N28','N34','N33']

# data_array format: row=step(0..n); columns: stages A0 B A1 X1 A2 X2 A3 X3
# for A0 + B*t + A1(1-np.exp(-X1*t)) + A2(1-np.exp(-X2*t)) + A3(1-np.exp(-X3*t))

include_exceeding_steps = True
include_matching_steps = True
include_lower_steps = True

# read fitted coefficients 
get_fitted_components(save_path+filename_start, string_list)  
    # reads fitted coefficients into sdict['fitted_values']
    # data_array format: row=step(0..n); columns: stages A0 B A1 X1 A2 X2 A3 X3
    # for A0 + B*t + A1*(1-np.exp(-X1*t)) + A2*(1-np.exp(-X2*t)) + A3*(1-np.exp(-X3*t))
    # creates sdict['fitted_steps'] arrays containing the elastic strain step at the start of each step response
    # also creates sdict['model_values'], same shape as sdict['fitted_values'], 
    # but containing E_el, eta_f, E_i, eta_i with units of GPa and GPa.day
# adjust N21 step 10 values to suppress plotting
N21['fitted_steps'][9] = np.nan
N21['fitted_values'][9,0] = 0  # set to zero K-V stages
N21['model_values'][9,0] = 0  # set to zero K-V stages


# plots of E_i, eta_i, 1/E_i, 1/eta_i against step number
#========================================================
print("\nPlots of fitted Kelvin-Voigt stage $E_i$ and $\eta_i$ values versus test step number\n")
fig=plt.figure(figsize=fig_size_3)
ax1=fig.add_subplot(221)
ax2=fig.add_subplot(222)
ax3=fig.add_subplot(223)
ax4=fig.add_subplot(224)
#
x_axis = np.arange(1,18,2)
x_values = [1,3,5,7,9,11,13,15,17]
mkr_size = 4
#
ax1.plot([0,18],[0,0],'k--',lw=0.5)
ax2.plot([0,18],[0,0],'k--',lw=0.5)
ax3.plot([0,18],[0,0],'k--',lw=0.5)
ax4.plot([0,18],[0,0],'k--',lw=0.5)
#
for string in string_list:
    sdict = globals()[string]
    # ignore last step where string broke
    end_index = sdict['test_steps'] if(string in ['N20','N21','N27']) else sdict['test_steps']-1
    for step_index in range(end_index):
        str_mkr = 'D' if(sdict['exceeding_steps'][step_index] == 1) \
             else 's' if(sdict['matching_steps'][step_index] == 1) else 'o'
        x_value = step_index + 1
        if((include_exceeding_steps and (str_mkr == 'D')) or
           (include_matching_steps and (str_mkr == 's')) or 
           (include_lower_steps and (str_mkr == 'o'))):
            for stage in range(int(sdict['model_values'][step_index,0])):
                mkr_pen = sdict['pen'] if(stage == 0) else 'none'
                y_value1 = sdict['model_values'][step_index,3+2*stage] # E_i (GPa)
                y_value2 = sdict['model_values'][step_index,4+2*stage] # eta_i (GPa.day)
                y_value3 = 1 / y_value1 # 1/E_i
                y_value4 = 1 / y_value2 # 1/eta_i
                ax1.plot(x_value, y_value1, color=sdict['pen'], ls='none', marker=str_mkr, ms=mkr_size, mfc=mkr_pen)
                ax2.plot(x_value, y_value2, color=sdict['pen'], ls='none', marker=str_mkr, ms=mkr_size, mfc=mkr_pen)
                ax3.plot(x_value, y_value3, color=sdict['pen'], ls='none', marker=str_mkr, ms=mkr_size, mfc=mkr_pen)
                ax4.plot(x_value, y_value4, color=sdict['pen'], ls='none', marker=str_mkr, ms=mkr_size, mfc=mkr_pen)
ax1.set_xticks(x_axis, x_values)
ax2.set_xticks(x_axis, x_values)
ax3.set_xticks(x_axis, x_values)
ax4.set_xticks(x_axis, x_values)
ax1.set_xlabel('Test step')
ax2.set_xlabel('Test step')
ax3.set_xlabel('Test step')
ax4.set_xlabel('Test step')
ax1.set_ylabel("$E_i$ (GPa)")
ax2.set_ylabel("$\eta_i$ (GPa.day)")
ax3.set_ylabel("$1/E_i$ (1/GPa)")
ax4.set_ylabel("$1/\eta_i$ (1/GPa.day)")
fig.tight_layout()
ax1.text(0.9, 0.9,'(a)', transform=ax1.transAxes)
ax2.text(0.9, 0.9,'(b)', transform=ax2.transAxes)
ax3.text(0.9, 0.9,'(c)', transform=ax3.transAxes)
ax4.text(0.9, 0.9,'(d)', transform=ax4.transAxes)
plt.show()


# plots of E_i, eta_i, 1/E_i, 1/eta_i against each other
#=======================================================
print("\nPlots of fitted Kelvin-Voigt stage $E_i$ and $\eta_i$ values against each other\n")
fig=plt.figure(figsize=fig_size_3)
ax1=fig.add_subplot(221)
ax2=fig.add_subplot(222)
ax3=fig.add_subplot(223)
ax4=fig.add_subplot(224)
#
mkr_size = 4
#
for string in string_list:
    sdict = globals()[string]
    # ignore last step where string broke
    end_index = sdict['test_steps'] if(string in ['N20','N21','N27']) else sdict['test_steps']-1
    for step_index in range(end_index):
        str_mkr = 'D' if(sdict['exceeding_steps'][step_index] == 1) \
             else 's' if(sdict['matching_steps'][step_index] == 1) else 'o'
        if((include_exceeding_steps and (str_mkr == 'D')) or
           (include_matching_steps and (str_mkr == 's')) or 
           (include_lower_steps and (str_mkr == 'o'))):
            for stage in range(int(sdict['model_values'][step_index,0])):
                mkr_pen = sdict['pen'] if(stage == 0) else 'none'
                x_value1 = sdict['model_values'][step_index,4+2*stage] # eta_i (GPa.day)
                x_value2 = x_value1
                x_value3 = 1 / x_value1 # 1/eta_i
                x_value4 = x_value3
                y_value1 = sdict['model_values'][step_index,3+2*stage] # E_i
                y_value2 = 1 / y_value1 # 1/E_i
                y_value3 = y_value1
                y_value4 = y_value2
                ax1.plot(x_value1, y_value1, color=sdict['pen'], ls='none', marker=str_mkr, ms=mkr_size, mfc=mkr_pen)
                ax2.plot(x_value2, y_value2, color=sdict['pen'], ls='none', marker=str_mkr, ms=mkr_size, mfc=mkr_pen)
                ax3.plot(x_value3, y_value3, color=sdict['pen'], ls='none', marker=str_mkr, ms=mkr_size, mfc=mkr_pen)
                ax4.plot(x_value4, y_value4, color=sdict['pen'], ls='none', marker=str_mkr, ms=mkr_size, mfc=mkr_pen)
ax1.set_xlabel("$\eta_i$ (GPa.day)")
ax2.set_xlabel("$\eta_i$ (GPa.day)")
ax3.set_xlabel("$1/\eta_i$ (1/GPa.day)")
ax4.set_xlabel("$1/\eta_i$ (1/GPa.day)")
ax1.set_ylabel("$E_i$ (GPa)")
ax2.set_ylabel("$1/E_i$ (1/GPa)")
ax3.set_ylabel("$E_i$ (GPa)")
ax4.set_ylabel("$1/E_i$ (1/GPa)")
fig.tight_layout()
ax1.text(0.9, 0.9,'(a)', transform=ax1.transAxes)
ax2.text(0.9, 0.9,'(b)', transform=ax2.transAxes)
ax3.text(0.9, 0.9,'(c)', transform=ax3.transAxes)
ax4.text(0.9, 0.9,'(d)', transform=ax4.transAxes)
plt.show()


# plots of Ai and Sigma(Ai) and Sigma(Ai) + elastic step against stress step
#===========================================================================
print("\nPlots of fitted Kelvin-Voigt stage $A_i$ coefficients, $\Sigma A_i$,")
print("and $\Sigma A_i$ + elastic strain step against applied stress step\n")
fig=plt.figure(figsize=fig_size_3)
ax1=fig.add_subplot(221)
ax2=fig.add_subplot(222)
ax3=fig.add_subplot(223)
ax4=fig.add_subplot(224)
#
x_range = np.array([-60,60])
mkr_size = 4
max_y1 = max_y2 = max_y3 = max_y4 = 0
grad_count = 0
grad_sum = 0
#
for string in string_list:
    sdict = globals()[string]
    # ignore last step where string broke
    end_index = sdict['test_steps'] if(string in ['N20','N21','N27']) else sdict['test_steps']-1
    for step_index in range(end_index):
        str_mkr = 'D' if(sdict['exceeding_steps'][step_index] == 1) \
             else 's' if(sdict['matching_steps'][step_index] == 1) else 'o'
        if((include_matching_steps and (str_mkr == 's')) or 
           (include_lower_steps and (str_mkr == 'o'))):
        # always exclude exceeding steps
            x_value = sdict['stress_n_step'][step_index]
            for stage in range(int(sdict['fitted_values'][step_index,0])):
                mkr_pen = sdict['pen'] if(stage == 0) else 'none'
                y_value1 = sdict['fitted_values'][step_index,3+2*stage] # A_i
                max_y1 = max(max_y1, abs(y_value1))
                ax1.plot(x_value, y_value1, color=sdict['pen'], ls='none', marker=str_mkr, ms=mkr_size, mfc=mkr_pen)
            y_value2 = np.sum([sdict['fitted_values'][step_index,3+2*stage] 
                              for stage in range(int(sdict['fitted_values'][step_index,0]))]) # Sigma(A_i)
            y_value3 = y_value2 + sdict['fitted_steps'][step_index]
            y_value4 = y_value3 - sdict['est_strain_step'][step_index]
            max_y2 = max(max_y2, abs(y_value2))
            max_y3 = max(max_y3, abs(y_value3))
            max_y4 = max(max_y4, abs(y_value4))
            ax2.plot(x_value, y_value2, color=sdict['pen'], ls='none', marker=str_mkr, ms=mkr_size, mfc='none')
            ax3.plot(x_value, y_value3, color=sdict['pen'], ls='none', marker=str_mkr, ms=mkr_size, mfc='none')
            ax4.plot(x_value, y_value4, color=sdict['pen'], ls='none', marker=str_mkr, ms=mkr_size, mfc='none')
            if((string == 'N28') and (step_index in [3,4,7,8])):
                ax4.plot(x_value, y_value4, color=sdict['pen'], ls='none', marker=str_mkr, ms=mkr_size, mfc=sdict['pen'])
            elif((sdict['exceeding_steps'][step_index] == 0) and (sdict['matching_steps'][step_index] == 0)
                 and ((string != 'N21') or (step_index != 9))):
                grad_count += 1
                grad_sum += y_value4 / x_value
max_y1 *= 1.1
max_y2 *= 1.1
max_y3 *= 1.1
max_y4 *= 1.1
ax1.plot(x_range,[0,0],'k--',lw=0.5)
ax1.plot([0,0],[-max_y1,max_y1],'k--',lw=0.5)
ax2.plot(x_range,[0,0],'k--',lw=0.5)
ax2.plot([0,0],[-max_y2,max_y2],'k--',lw=0.5)
ax3.plot(x_range,[0,0],'k--',lw=0.5)
ax3.plot([0,0],[-max_y3,max_y3],'k--',lw=0.5)
ax4.plot(x_range,[0,0],'k--',lw=0.5)
ax4.plot([0,0],[-max_y4,max_y4],'k--',lw=0.5)
#
grad_value = grad_sum / grad_count
print('average gradient =', grad_value)
print('equivalent modulus =', 1/(1000*grad_value), 'GPa')
ax4.plot(x_range,grad_value*x_range,'k--',lw=0.5)
#
ax1.set_xlabel('Stress step (MPa)')
ax2.set_xlabel('Stress step (MPa)')
ax3.set_xlabel('Stress step (MPa)')
ax4.set_xlabel('Stress step (MPa)')
ax1.set_ylabel("$A_i$")
ax2.set_ylabel("$\Sigma A_i$")
ax3.set_ylabel("Elastic strain step + $\Sigma A_i$")
ax4.set_ylabel("$\Sigma A_i$ adjusted")
fig.tight_layout()
ax1.text(0.05, 0.9,'(a)', transform=ax1.transAxes)
ax2.text(0.05, 0.9,'(b)', transform=ax2.transAxes)
ax3.text(0.05, 0.9,'(c)', transform=ax3.transAxes)
ax4.text(0.05, 0.9,'(d)', transform=ax4.transAxes)
plt.show()


# plots for paper
#=======================================================
print("\nPlots for paper\n")

fig=plt.figure(figsize=fig_size_2)
ax1=fig.add_subplot(121)
ax2=fig.add_subplot(122)
#
mkr_size = 4
max_y2 = 0
grad_count = 0
grad_sum = 0
#
for string in string_list:
    sdict = globals()[string]
    # add a label for every string
    ax2.plot(-100, 0, color=sdict['pen'], ls='none', marker='s', ms=mkr_size, mfc=sdict['pen'], label=sdict['leg'])
    # ignore last step where string broke
    end_index = sdict['test_steps'] if(string in ['N20','N21','N27']) else sdict['test_steps']-1
    for step_index in range(end_index):
        str_mkr = 'x' if(sdict['exceeding_steps'][step_index] == 1) \
             else 's' if(sdict['matching_steps'][step_index] == 1) else 'o'
        x_value2 = sdict['stress_n_step'][step_index]
        for stage in range(int(sdict['model_values'][step_index,0])):
            x_value1 = sdict['model_values'][step_index,4+2*stage] # eta_i (GPa.day)
            y_value1 = 1 / sdict['model_values'][step_index,3+2*stage] # 1/E_i
            ax1.plot(x_value1, y_value1, color=sdict['pen'], ls='none', marker=str_mkr, ms=mkr_size, mfc='none')
        if(sdict['exceeding_steps'][step_index] == 0): 
            y_value2 = sdict['fitted_steps'][step_index] \
                       + np.sum([sdict['fitted_values'][step_index,3+2*stage] 
                                 for stage in range(int(sdict['fitted_values'][step_index,0]))]) # Sigma(A_i)
            ax2.plot(x_value2, y_value2, color=sdict['pen'], ls='none', marker=str_mkr, ms=mkr_size, mfc='none')
            max_y2 = max(max_y2, abs(y_value2))
            if((string == 'N28') and (step_index in [3,4,7,8])):
                ax2.plot(x_value2, y_value2, color=sdict['pen'], ls='none', marker=str_mkr, ms=mkr_size, mfc=sdict['pen'])
            elif((sdict['exceeding_steps'][step_index] == 0) and (sdict['matching_steps'][step_index] == 0)
                 and ((string != 'N21') or (step_index != 9))):
                grad_count += 1
                grad_sum += y_value2 / x_value2
x_range = np.array([-60,60])
max_y2 *= 1.1
grad_value = grad_sum / grad_count
print('average gradient =', grad_value)
ax2.plot(x_range,[0,0],'k--',lw=0.5)
ax2.plot([0,0],[-max_y2,max_y2],'k--',lw=0.5)
ax2.plot(x_range,grad_value*x_range,'k--',lw=0.5)
#
ax2.axis([-64,64,-0.019,0.019])
ax2.legend(loc='lower right', ncol = 2, prop={'size':8}, framealpha=1)
#
ax1.set_xlabel("Viscosity $\eta_i$ (GPa.day)")
ax2.set_xlabel('Stress step (MPa)')
ax1.set_ylabel("Compliance $1/E_i$ (1/GPa)")
ax2.set_ylabel("Amplitude $A_{el} + \Sigma A_i$")
#
fig.tight_layout()
ax1.text(0.9, 0.9,'(a)', transform=ax1.transAxes)
ax2.text(0.05, 0.9,'(b)', transform=ax2.transAxes)
savefile = save_path + "recoverable_creep_study." + save_format
print("%s" % (savefile))
#fig.savefig(savefile, format=save_format, dpi=1000)
plt.show()
```

```
Plots of fitted Kelvin-Voigt stage $E_i$ and $\eta_i$ values versus test step number
```

```
Plots of fitted Kelvin-Voigt stage $E_i$ and $\eta_i$ values against each other
```

```
Plots of fitted Kelvin-Voigt stage $A_i$ coefficients, $\Sigma A_i$,
and $\Sigma A_i$ + elastic strain step against applied stress step

average gradient = 4.145880088141474e-05
equivalent modulus = 24.12033099703765 GPa
```

```
Plots for paper

average gradient = 0.00014915256069865534
./recoverable_creep_study.eps
```

### Multi-step fitting for recoverable creep¶

In [7]:

```
N21_version = 'N21m'
txn_step = 11  # first step after step 10 cut

def test_creep_function_1(time_array, A0, B, inv_E1, inv_tau1):
    sdict = globals()[N21_version] if(fit_string == 'N21') else globals()[fit_string]
    el_strain_array = np.zeros_like(time_array)
    creep_array_1 = np.zeros_like(time_array)
    tail_array = np.zeros_like(time_array)
    extra_stress = 0.0
    if(fit_steps[0] > 1):
        el_strain_array[0] = sdict['el_strain_est'][fit_steps[0]-2]  # elastic strain at start of selected step range
    for step in fit_steps:  # step numbers to fit
        step_index = step - fit_steps[0]  # need relative step index because using sub-arrays
        start_index = fit_step_starts[step_index] + 1  # don't include first point of step (at previous stress level)
        stop_index = fit_step_starts[step_index+1]
        stop_index += 1 if(step < sdict['test_steps']) else 0
            # include first point of next step (at current stress level), except for very last step
        neg_time_sub_array = time_array[start_index-1] - time_array[start_index:stop_index] # negative time since step start
        extra_stress += sdict['stress_n_step'][step-1]
        total_stress = sdict['stress_n'][step-1]
        # elastic strain component
        el_strain_array[start_index:stop_index] = sdict['el_strain_est'][step-1]
        # creep component
        base = creep_array_1[start_index-1] # last point calculated = first point of current step (0 at start of first step)
        target = extra_stress * inv_E1
        creep_array_1[start_index:stop_index] = base + (base - target) * np.expm1(inv_tau1 * neg_time_sub_array)
        # final dashpot component
        tail_array[start_index:stop_index] = tail_array[start_index-1] - total_stress * B * neg_time_sub_array
    return A0 + el_strain_array + creep_array_1 + tail_array

def test_creep_function_2(time_array, A0, B, inv_E1, inv_tau1, inv_E2, inv_tau2):
    sdict = globals()[N21_version] if(fit_string == 'N21') else globals()[fit_string]
    el_strain_array = np.zeros_like(time_array)
    creep_array_1 = np.zeros_like(time_array)
    creep_array_2 = np.zeros_like(time_array)
    tail_array = np.zeros_like(time_array)
    extra_stress = 0.0
    if(fit_steps[0] > 1):
        el_strain_array[0] = sdict['el_strain_est'][fit_steps[0]-2]  # elastic strain at start of selected step range
    for step in fit_steps:  # step numbers to fit
        step_index = step - fit_steps[0]  # need relative step index because using sub-arrays
        start_index = fit_step_starts[step_index] + 1  # don't include first point of step (at previous stress level)
        stop_index = fit_step_starts[step_index+1]
        stop_index += 1 if(step < sdict['test_steps']) else 0
            # include first point of next step (at current stress level), except for very last step
        neg_time_sub_array = time_array[start_index-1] - time_array[start_index:stop_index] # negative time since step start
        extra_stress += sdict['stress_n_step'][step-1]
        total_stress = sdict['stress_n'][step-1]
        # elastic strain component
        el_strain_array[start_index:stop_index] = sdict['el_strain_est'][step-1]
        # creep component 1
        base = creep_array_1[start_index-1] # last point calculated = first point of current step (0 at start of first step)
        target = extra_stress * inv_E1
        creep_array_1[start_index:stop_index] = base + (base - target) * np.expm1(inv_tau1 * neg_time_sub_array)
        # creep component 2
        base = creep_array_2[start_index-1] # last point calculated = first point of current step (0 at start of first step)
        target = extra_stress * inv_E2
        creep_array_2[start_index:stop_index] = base + (base - target) * np.expm1(inv_tau2 * neg_time_sub_array)
        # final dashpot component
        tail_array[start_index:stop_index] = tail_array[start_index-1] - total_stress * B * neg_time_sub_array
    return A0 + el_strain_array + creep_array_1 + creep_array_2 + tail_array

def test_creep_function_2x(time_array, A0, B, inv_E1, inv_tau1, inv_E2, inv_tau2, tweak_value):
    sdict = globals()[N21_version] if(fit_string == 'N21') else globals()[fit_string]
    el_strain_array = np.zeros_like(time_array)
    creep_array_1 = np.zeros_like(time_array)
    creep_array_2 = np.zeros_like(time_array)
    tail_array = np.zeros_like(time_array)
    tweak_array = np.zeros_like(time_array)
    extra_stress = 0.0
    if(fit_steps[0] > 1):
        el_strain_array[0] = sdict['el_strain_est'][fit_steps[0]-2]  # elastic strain at start of selected step range
    for step in fit_steps:  # step numbers to fit
        step_index = step - fit_steps[0]  # need relative step index because using sub-arrays
        start_index = fit_step_starts[step_index] + 1  # don't include first point of step (at previous stress level)
        stop_index = fit_step_starts[step_index+1]
        stop_index += 1 if(step < sdict['test_steps']) else 0
            # include first point of next step (at current stress level), except for very last step
        neg_time_sub_array = time_array[start_index-1] - time_array[start_index:stop_index] # negative time since step start
        extra_stress += sdict['stress_n_step'][step-1]
        total_stress = sdict['stress_n'][step-1]
        # elastic strain component
        el_strain_array[start_index:stop_index] = sdict['el_strain_est'][step-1]
        # creep component 1
        base = creep_array_1[start_index-1] # last point calculated = first point of current step (0 at start of first step)
        target = extra_stress * inv_E1
        creep_array_1[start_index:stop_index] = base + (base - target) * np.expm1(inv_tau1 * neg_time_sub_array)
        # creep component 2
        base = creep_array_2[start_index-1] # last point calculated = first point of current step (0 at start of first step)
        target = extra_stress * inv_E2
        creep_array_2[start_index:stop_index] = base + (base - target) * np.expm1(inv_tau2 * neg_time_sub_array)
        # final dashpot component
        tail_array[start_index:stop_index] = tail_array[start_index-1] - total_stress * B * neg_time_sub_array
        # amplitude tweak across cut point
        if(step >= txn_step):
            tweak_array[start_index:stop_index] = tweak_value
    return A0 + el_strain_array + creep_array_1 + creep_array_2 + tail_array + tweak_array

def test_creep_function_3(time_array, A0, B, inv_E1, inv_tau1, inv_E2, inv_tau2, inv_E3, inv_tau3):
    sdict = globals()[N21_version] if(fit_string == 'N21') else globals()[fit_string]
    el_strain_array = np.zeros_like(time_array)
    creep_array_1 = np.zeros_like(time_array)
    creep_array_2 = np.zeros_like(time_array)
    creep_array_3 = np.zeros_like(time_array)
    tail_array = np.zeros_like(time_array)
    extra_stress = 0.0
    if(fit_steps[0] > 1):
        el_strain_array[0] = sdict['el_strain_est'][fit_steps[0]-2]  # elastic strain at start of selected step range
    for step in fit_steps:  # step numbers to fit
        step_index = step - fit_steps[0]  # need relative step index because using sub-arrays
        start_index = fit_step_starts[step_index] + 1  # don't include first point of step (at previous stress level)
        stop_index = fit_step_starts[step_index+1]
        stop_index += 1 if(step < sdict['test_steps']) else 0
            # include first point of next step (at current stress level), except for very last step
        neg_time_sub_array = time_array[start_index-1] - time_array[start_index:stop_index] # negative time since step start
        extra_stress += sdict['stress_n_step'][step-1]
        total_stress = sdict['stress_n'][step-1]
        # elastic strain component
        el_strain_array[start_index:stop_index] = sdict['el_strain_est'][step-1]
        # creep component 1
        base = creep_array_1[start_index-1] # last point calculated = first point of current step (0 at start of first step)
        target = extra_stress * inv_E1
        creep_array_1[start_index:stop_index] = base + (base - target) * np.expm1(inv_tau1 * neg_time_sub_array)
        # creep component 2
        base = creep_array_2[start_index-1] # last point calculated = first point of current step (0 at start of first step)
        target = extra_stress * inv_E2
        creep_array_2[start_index:stop_index] = base + (base - target) * np.expm1(inv_tau2 * neg_time_sub_array)
        # creep component 3
        base = creep_array_3[start_index-1] # last point calculated = first point of current step (0 at start of first step)
        target = extra_stress * inv_E3
        creep_array_3[start_index:stop_index] = base + (base - target) * np.expm1(inv_tau3 * neg_time_sub_array)
        # final dashpot component
        tail_array[start_index:stop_index] = tail_array[start_index-1] - total_stress * B * neg_time_sub_array
    return A0 + el_strain_array + creep_array_1 + creep_array_2 + creep_array_3 + tail_array

def multi_step_fit(fit_string_value, start_step, stop_step, stages, spanning_cut):
    global fit_string
    global fit_steps
    global fit_step_starts
    global start_stress
    global time_sub_array
    global strain_sub_array
    global fitted_curve
    global el_strain_array
    global creep_array_1
    global creep_array_2
    global creep_array_3
    global tail_array
    global tweak_array
    global check_array
    #
    fit_string = fit_string_value
    fit_steps = np.arange(start_step,stop_step+1,1)
    print('\nFitting string', fit_string, 'steps', start_step, 'to', stop_step)
    sdict = globals()[N21_version] if(fit_string == 'N21') else globals()[fit_string]
    #
    start_index = sdict['step_starts'][start_step-1]
    stop_index = sdict['step_starts'][stop_step]
    stop_index += 1 if(stop_step < sdict['test_steps']) else 0
    time_sub_array = sdict['time_array'][start_index:stop_index]
    strain_sub_array = sdict['strain_array'][start_index:stop_index]
    time_for_fitting = time_sub_array - sdict['time_array'][start_index]
    #
    fit_step_starts = np.copy(sdict['step_starts'][start_step-1:stop_step+1])
    fit_step_starts -= fit_step_starts[0]
    start_stress = 0 if(start_step == 1) else sdict['stress_n'][start_step-2]  # applied stress for step before start_step
    #
    # curve fitting
    if(spanning_cut and (stages == 2)): # spanning cut step
        popt, pcov = curve_fit(test_creep_function_2x, time_for_fitting, strain_sub_array, 
                               bounds=([-0.5,-0.001,0,1,0,0,-0.01],[0.5,0.001,0.001,50,0.001,5,0.01]))
        fitted_curve = test_creep_function_2x(time_for_fitting, *popt) 
    else:
        if(stages == 1):
            popt, pcov = curve_fit(test_creep_function_1, time_for_fitting, strain_sub_array, 
                                   bounds=([-0.5,-0.001,0,0],[0.5,0.001,0.0001,5]))
            fitted_curve = test_creep_function_1(time_for_fitting, *popt) 
        elif(stages == 2):
            popt, pcov = curve_fit(test_creep_function_2, time_for_fitting, strain_sub_array, 
                                   bounds=([-0.5,-0.001,0,1,0,0],[0.5,0.001,0.001,50,0.001,5]))
            fitted_curve = test_creep_function_2(time_for_fitting, *popt) 
        else:
            popt, pcov = curve_fit(test_creep_function_3, time_for_fitting, strain_sub_array, 
                                   bounds=([-0.5,-0.001,0,1,0,0,0,0],[0.5,0.001,0.001,50,0.001,5,0.001,50]))
            fitted_curve = test_creep_function_3(time_for_fitting, *popt) 
    #
    # reconstruct fitted components
    el_strain_array = np.zeros_like(time_for_fitting)
    creep_array_1 = np.zeros_like(time_for_fitting)
    creep_array_2 = np.zeros_like(time_for_fitting)
    creep_array_3 = np.zeros_like(time_for_fitting)
    tail_array = np.zeros_like(time_for_fitting)
    tweak_array = np.zeros_like(time_for_fitting)
    extra_stress = 0.0
    if(fit_steps[0] > 1):
        el_strain_array[0] = sdict['el_strain_est'][fit_steps[0]-2]  # elastic strain at start of selected step range
    for step in fit_steps:  # step numbers to fit
        step_index = step - fit_steps[0]  # need relative step index because using sub-arrays
        start_index = fit_step_starts[step_index] + 1  # don't include first point of step (at previous stress level)
        stop_index = fit_step_starts[step_index+1]
        stop_index += 1 if(step < sdict['test_steps']) else 0
            # include first point of next step (at current stress level), except for very last step
        neg_time_sub_array = time_for_fitting[start_index-1] - time_for_fitting[start_index:stop_index] # negative time since step start
        extra_stress += sdict['stress_n_step'][step-1]
        total_stress = sdict['stress_n'][step-1]
        el_strain_array[start_index:stop_index] = sdict['el_strain_est'][step-1]
        # final dashpot component
        tail_array[start_index:stop_index] = tail_array[start_index-1] - total_stress * popt[1] * neg_time_sub_array
        # creep component 1
        base = creep_array_1[start_index-1] # last point calculated = first point of current step (0 at start of first step)
        target = extra_stress * popt[2]
        creep_array_1[start_index:stop_index] = base + (base - target) * np.expm1(popt[3] * neg_time_sub_array)
        results_str1 = ('A0 %0.5f, K %0.3f, G %0.6f, B %e, K1 %0.8f, X1 %0.3f' 
                        % (popt[0], sdict['E0_est'], Et_coeff_b, popt[1], popt[2], popt[3]))
        results_str2 = ('etaf %f GPa.day, E1 %f GPa, eta1 %f GPa.day' 
                        % (1/(1000*popt[1]), 1/(1000*popt[2]), 1/(1000*popt[2]*popt[3])))
        table_string = ('%s & %d--%d & %.1f & %.3f' 
                        % (fit_string, start_step, stop_step, 1/(1000*popt[2]), 1/(1000*popt[2]*popt[3])))
        if(stages > 1):
            # creep component 2
            base = creep_array_2[start_index-1] # last point calculated = first point of current step (0 at start of first step)
            target = extra_stress * popt[4]
            creep_array_2[start_index:stop_index] = base + (base - target) * np.expm1(popt[5] * neg_time_sub_array)
            results_str1 += (', K2 %0.8f, X2 %0.3f' % (popt[4], popt[5]))
            results_str2 += (', E2 %f GPa, eta2 %f MPa.s' % (1/(1000*popt[4]), 1/(1000*popt[4]*popt[5])))
            table_string += (' & %.1f & %.2f' % (1/(1000*popt[4]), 1/(1000*popt[4]*popt[5])))
        if(stages > 2):
            # creep component 3
            base = creep_array_3[start_index-1] # last point calculated = first point of current step (0 at start of first step)
            target = extra_stress * popt[6]
            creep_array_3[start_index:stop_index] = base + (base - target) * np.expm1(popt[7] * neg_time_sub_array)
            results_str1 += (', K3 %0.8f, X3 %0.3f' % (popt[6], popt[7]))
            results_str2 += (', E3 %f GPa, eta3 %f MPa.s' % (1/(1000*popt[6]), 1/(1000*popt[6]*popt[7])))
        table_string += (' & %.1f' % (1/(1000*popt[1])))
        # amplitude tweak across cut point
        if(spanning_cut and (stages == 2) and (step >= txn_step)):
            tweak_array[start_index:stop_index] = popt[-1]
            results_str1 += (', tweak %f' % (popt[-1]))
            table_string += (' & %.5f' % (popt[-1]))
    check_array = popt[0] + el_strain_array + creep_array_1 + creep_array_2 + creep_array_3 + tail_array + tweak_array
    print(results_str1)
    print(results_str2)
#    print(table_string)
#    print(popt)
    return popt

def multi_step_fit_plot_ax(ax, fit_string_value, start_step, stop_step, stages):
    # check if spanning N21 step 10 to 11
    spanning_cut = ((fit_string_value == 'N21') and (start_step < txn_step) and (stop_step > (txn_step-1)))
    # run curve fitting
    popt = multi_step_fit(fit_string_value, start_step, stop_step, stages, spanning_cut)
    # initial data
    ax.plot(time_sub_array, strain_sub_array, 'k-', label='strain data')
    ax.plot(time_sub_array, fitted_curve, 'r--', label='fitted curve')
#    ax.plot(time_sub_array, check_array, 'b--', label='check curve')
    # plot component parts of fitted curve
    plot_offset = min(fitted_curve)
#    ax.plot(time_sub_array, [popt[0] for i in range(len(time_sub_array))], 'k:', label='offset')
#    ax.plot(time_sub_array, plot_offset+el_strain_array-el_strain_array[0], 'k:', label='spring')
    ax.plot(time_sub_array, plot_offset+creep_array_1, 'b:', label='main K-V stage')
    if(stages==1):
        ax.plot(time_sub_array, plot_offset+tail_array, 'g:', label='dashpot')
    if(stages==2):
#        ax.plot(time_sub_array, plot_offset+creep_array_2, 'r:', label='2nd K-V stage')
#        ax.plot(time_sub_array, plot_offset+tail_array, 'g:', label='dashpot')
        ax.plot(time_sub_array, plot_offset+creep_array_2+tail_array, ':', color='orange', label='slower components')
    if(stages>2):
        ax.plot(time_sub_array, plot_offset+creep_array_2, 'r:', label='2nd K-V stage')
        ax.plot(time_sub_array, plot_offset+creep_array_3, 'r:', label='3rd K-V stage')
    fit_steps_string = str(start_step) + ' to ' + str(stop_step)
    ax.set_title('String ' + str(fit_string_value) + ', steps ' + fit_steps_string)
    ax.set_xlabel('Time (days)')
    ax.set_ylabel('Strain')

def multi_step_fit_plot(fit_string_value, start_step, stop_step, stages):
    # plot data    
    fig=plt.figure(figsize=(8,5))
    ax1=fig.add_subplot(111)
    multi_step_fit_plot_ax(ax1, fit_string_value, start_step, stop_step, stages)
    fig.tight_layout()
    plt.show()

    
# combined plot
# =============
fig=plt.figure(figsize=fig_size_3)
ax1=fig.add_subplot(221)
ax2=fig.add_subplot(222)
ax3=fig.add_subplot(223)
ax4=fig.add_subplot(224)
#
multi_step_fit_plot_ax(ax1,'N20', 3, 8, 2)    # 6 steps
multi_step_fit_plot_ax(ax2,'N21', 7, 15, 2)   # 7/8 steps
multi_step_fit_plot_ax(ax3,'N27', 3, 15, 2)   # 13 steps
multi_step_fit_plot_ax(ax4,'N33', 3, 6, 2)    # 4 steps
#
fig.tight_layout()
ax1.text(0.9, 0.9,'(a)', transform=ax1.transAxes)
ax2.text(0.05, 0.9,'(b)', transform=ax2.transAxes)
ax3.text(0.05, 0.9,'(c)', transform=ax3.transAxes)
ax4.text(0.05, 0.9,'(d)', transform=ax4.transAxes)
savefile = save_path + "recoverable_creep_multi_step." + save_format
print("\n%s" % (savefile))
#fig.savefig(savefile, format=save_format, dpi=1000)
plt.show()


# individual plots
# ================
# N20
multi_step_fit_plot('N20', 3, 8, 2)  # 6 steps

# for comparison with Murphy's fits
multi_step_fit_plot('N20', 3, 8, 1)  # 6 steps
multi_step_fit_plot('N21', 6, 9, 1)

# N21 before and after cut
multi_step_fit_plot('N21', 7, 9, 2)
multi_step_fit_plot('N21', 11, 15, 2)

# N21
multi_step_fit_plot('N21', 2, 5, 2)
multi_step_fit_plot('N21', 2, 6, 2)
for stop_step in range(10,17,1):
    multi_step_fit_plot('N21', 7, stop_step, 2)
for start_step in range(8,12,1):
    multi_step_fit_plot('N21', start_step, 15, 2)
multi_step_fit_plot('N21', 8, 16, 2)

# N27
for stop_step in range(5,17,1):
    multi_step_fit_plot('N27', 3, stop_step, 2)

# N33
multi_step_fit_plot('N33', 3, 5, 2)  # 3 steps
multi_step_fit_plot('N33', 3, 6, 2)  # 4 steps
#multi_step_fit_plot('N33', 3, 7, 2)  # 5 steps

# N28
multi_step_fit_plot('N28', 4, 5, 2)  # 2 steps
#multi_step_fit_plot('N28', 4, 6, 2)  # 2 steps
multi_step_fit_plot('N28', 8, 9, 2)  # 2 steps
#multi_step_fit_plot('N28', 8, 10, 2)  # 3 steps
multi_step_fit_plot('N28', 12, 14, 2)  # 3 steps
multi_step_fit_plot('N28', 12, 15, 2)  # 4 steps
#multi_step_fit_plot('N28', 12, 16, 2)  # 5 steps
```

```
Fitting string N20 steps 3 to 8
A0 0.04811, K 3.009, G 0.035282, B -3.835463e-07, K1 0.00001726, X1 4.925, K2 0.00008706, X2 0.084
etaf -2607.247026 GPa.day, E1 57.924007 GPa, eta1 11.760714 GPa.day, E2 11.486112 GPa, eta2 137.248974 MPa.s

Fitting string N21 steps 7 to 15
A0 0.04369, K 3.484, G 0.035282, B 9.027710e-08, K1 0.00001318, X1 3.980, K2 0.00003094, X2 0.216, tweak 0.000559
etaf 11077.006656 GPa.day, E1 75.888645 GPa, eta1 19.067169 GPa.day, E2 32.319116 GPa, eta2 149.547814 MPa.s

Fitting string N27 steps 3 to 15
A0 0.08167, K 2.957, G 0.035282, B 6.558682e-07, K1 0.00003565, X1 4.081, K2 0.00012460, X2 0.046
etaf 1524.696578 GPa.day, E1 28.053432 GPa, eta1 6.874691 GPa.day, E2 8.025462 GPa, eta2 174.921517 MPa.s

Fitting string N33 steps 3 to 6
A0 0.09865, K 2.946, G 0.035282, B 1.096447e-06, K1 0.00003369, X1 3.452, K2 0.00016192, X2 0.038
etaf 912.036602 GPa.day, E1 29.686492 GPa, eta1 8.600765 GPa.day, E2 6.175867 GPa, eta2 161.183357 MPa.s

./recoverable_creep_multi_step.eps
```

```
Fitting string N20 steps 3 to 8
A0 0.04811, K 3.009, G 0.035282, B -3.835463e-07, K1 0.00001726, X1 4.925, K2 0.00008706, X2 0.084
etaf -2607.247026 GPa.day, E1 57.924007 GPa, eta1 11.760714 GPa.day, E2 11.486112 GPa, eta2 137.248974 MPa.s
```

```
Fitting string N20 steps 3 to 8
A0 0.04779, K 3.009, G 0.035282, B -5.076333e-07, K1 0.00006700, X1 0.176
etaf -1969.925846 GPa.day, E1 14.924934 GPa, eta1 84.700860 GPa.day
```

```
Fitting string N21 steps 6 to 9
A0 0.03906, K 3.484, G 0.035282, B 6.506295e-07, K1 0.00007785, X1 0.192
etaf 1536.973001 GPa.day, E1 12.844750 GPa, eta1 66.916858 GPa.day
```

```
Fitting string N21 steps 7 to 9
A0 0.04372, K 3.484, G 0.035282, B 5.023014e-07, K1 0.00002189, X1 4.454, K2 0.00006887, X2 0.076
etaf 1990.836519 GPa.day, E1 45.688617 GPa, eta1 10.257616 GPa.day, E2 14.520919 GPa, eta2 191.838415 MPa.s
```

```
Fitting string N21 steps 11 to 15
A0 0.04304, K 3.484, G 0.035282, B 9.949628e-08, K1 0.00002240, X1 2.583, K2 0.00009680, X2 0.043
etaf 10050.627177 GPa.day, E1 44.641397 GPa, eta1 17.285531 GPa.day, E2 10.330320 GPa, eta2 239.681808 MPa.s
```

```
Fitting string N21 steps 2 to 5
A0 0.03792, K 3.484, G 0.035282, B 3.543759e-08, K1 0.00002533, X1 3.001, K2 0.00003843, X2 0.102
etaf 28218.624751 GPa.day, E1 39.479239 GPa, eta1 13.154473 GPa.day, E2 26.019000 GPa, eta2 256.227952 MPa.s
```

```
Fitting string N21 steps 2 to 6
A0 0.03795, K 3.484, G 0.035282, B 7.504868e-07, K1 0.00003292, X1 2.256, K2 0.00100000, X2 0.005
etaf 1332.468485 GPa.day, E1 30.377449 GPa, eta1 13.462880 GPa.day, E2 1.000000 GPa, eta2 212.641752 MPa.s
```

```
Fitting string N21 steps 7 to 10
A0 0.04379, K 3.484, G 0.035282, B 6.889421e-08, K1 0.00001771, X1 5.264, K2 0.00002666, X2 0.226
etaf 14515.007780 GPa.day, E1 56.467988 GPa, eta1 10.727496 GPa.day, E2 37.507296 GPa, eta2 166.018559 MPa.s
```

```
Fitting string N21 steps 7 to 11
A0 0.04376, K 3.484, G 0.035282, B 3.059573e-07, K1 0.00002705, X1 2.041, K2 0.00006058, X2 0.048, tweak 0.000157
etaf 3268.430202 GPa.day, E1 36.972960 GPa, eta1 18.116544 GPa.day, E2 16.508105 GPa, eta2 342.335455 MPa.s
```

```
Fitting string N21 steps 7 to 12
A0 0.04377, K 3.484, G 0.035282, B 2.856302e-07, K1 0.00002499, X1 2.780, K2 0.00005263, X2 0.063, tweak 0.000137
etaf 3501.030827 GPa.day, E1 40.014605 GPa, eta1 14.391604 GPa.day, E2 19.002231 GPa, eta2 300.867972 MPa.s
```

```
Fitting string N21 steps 7 to 13
A0 0.04382, K 3.484, G 0.035282, B 1.766693e-07, K1 0.00002067, X1 4.112, K2 0.00003632, X2 0.132, tweak 0.000219
etaf 5660.292486 GPa.day, E1 48.371252 GPa, eta1 11.764098 GPa.day, E2 27.535328 GPa, eta2 208.574592 MPa.s
```

```
Fitting string N21 steps 7 to 14
A0 0.04376, K 3.484, G 0.035282, B 1.255284e-07, K1 0.00001570, X1 4.409, K2 0.00003359, X2 0.186, tweak 0.000385
etaf 7966.323873 GPa.day, E1 63.693547 GPa, eta1 14.445467 GPa.day, E2 29.774741 GPa, eta2 160.052750 MPa.s
```

```
Fitting string N21 steps 7 to 15
A0 0.04369, K 3.484, G 0.035282, B 9.027710e-08, K1 0.00001318, X1 3.980, K2 0.00003094, X2 0.216, tweak 0.000559
etaf 11077.006656 GPa.day, E1 75.888645 GPa, eta1 19.067169 GPa.day, E2 32.319116 GPa, eta2 149.547814 MPa.s
```

```
Fitting string N21 steps 7 to 16
A0 0.04414, K 3.484, G 0.035282, B 9.154712e-07, K1 0.00003026, X1 3.357, K2 0.00100000, X2 0.005, tweak -0.000259
etaf 1092.333626 GPa.day, E1 33.051472 GPa, eta1 9.846245 GPa.day, E2 1.000000 GPa, eta2 187.122827 MPa.s
```

```
Fitting string N21 steps 8 to 15
A0 0.04262, K 3.484, G 0.035282, B 8.078797e-08, K1 0.00002030, X1 2.396, K2 0.00004895, X2 0.092, tweak 0.000352
etaf 12378.080547 GPa.day, E1 49.262333 GPa, eta1 20.556516 GPa.day, E2 20.427792 GPa, eta2 222.196547 MPa.s
```

```
Fitting string N21 steps 9 to 15
A0 0.04134, K 3.484, G 0.035282, B 7.023838e-08, K1 0.00001458, X1 2.893, K2 0.00003086, X2 0.186, tweak 0.000491
etaf 14237.231023 GPa.day, E1 68.610463 GPa, eta1 23.718587 GPa.day, E2 32.402015 GPa, eta2 174.331648 MPa.s
```

```
Fitting string N21 steps 10 to 15
A0 0.04253, K 3.484, G 0.035282, B 9.711635e-08, K1 0.00002264, X1 2.551, K2 0.00010321, X2 0.040, tweak 0.000377
etaf 10296.927480 GPa.day, E1 44.169793 GPa, eta1 17.314351 GPa.day, E2 9.688581 GPa, eta2 241.714265 MPa.s
```

```
Fitting string N21 steps 11 to 15
A0 0.04304, K 3.484, G 0.035282, B 9.949628e-08, K1 0.00002240, X1 2.583, K2 0.00009680, X2 0.043
etaf 10050.627177 GPa.day, E1 44.641397 GPa, eta1 17.285531 GPa.day, E2 10.330320 GPa, eta2 239.681808 MPa.s
```

```
Fitting string N21 steps 8 to 16
A0 0.04351, K 3.484, G 0.035282, B 1.312294e-07, K1 0.00003002, X1 8.942, K2 0.00100000, X2 0.008, tweak -0.000454
etaf 7620.246339 GPa.day, E1 33.308544 GPa, eta1 3.724967 GPa.day, E2 1.000000 GPa, eta2 126.667164 MPa.s
```

```
Fitting string N27 steps 3 to 5
A0 0.08162, K 2.957, G 0.035282, B 2.355209e-07, K1 0.00003197, X1 10.572, K2 0.00004367, X2 0.109
etaf 4245.908081 GPa.day, E1 31.276888 GPa, eta1 2.958412 GPa.day, E2 22.901310 GPa, eta2 210.421224 MPa.s
```

```
Fitting string N27 steps 3 to 6
A0 0.08140, K 2.957, G 0.035282, B 5.017653e-07, K1 0.00002752, X1 9.028, K2 0.00007599, X2 0.077
etaf 1992.963731 GPa.day, E1 36.334864 GPa, eta1 4.024718 GPa.day, E2 13.160128 GPa, eta2 171.916549 MPa.s
```

```
Fitting string N27 steps 3 to 7
A0 0.08135, K 2.957, G 0.035282, B 4.917310e-07, K1 0.00002472, X1 11.964, K2 0.00006952, X2 0.093
etaf 2033.632161 GPa.day, E1 40.452788 GPa, eta1 3.381201 GPa.day, E2 14.383570 GPa, eta2 155.305017 MPa.s
```

```
Fitting string N27 steps 3 to 8
A0 0.08139, K 2.957, G 0.035282, B 4.369206e-07, K1 0.00002388, X1 16.007, K2 0.00006200, X2 0.112
etaf 2288.745401 GPa.day, E1 41.874023 GPa, eta1 2.616041 GPa.day, E2 16.128288 GPa, eta2 144.442951 MPa.s
```

```
Fitting string N27 steps 3 to 9
A0 0.08155, K 2.957, G 0.035282, B 5.701206e-07, K1 0.00002974, X1 11.419, K2 0.00009706, X2 0.062
etaf 1754.014743 GPa.day, E1 33.625255 GPa, eta1 2.944775 GPa.day, E2 10.303246 GPa, eta2 164.886060 MPa.s
```

```
Fitting string N27 steps 3 to 10
A0 0.08141, K 2.957, G 0.035282, B 7.337261e-07, K1 0.00003145, X1 5.993, K2 0.00016253, X2 0.034
etaf 1362.906456 GPa.day, E1 31.800247 GPa, eta1 5.306108 GPa.day, E2 6.152812 GPa, eta2 182.629804 MPa.s
```

```
Fitting string N27 steps 3 to 11
A0 0.08137, K 2.957, G 0.035282, B 7.709369e-07, K1 0.00003157, X1 5.517, K2 0.00018477, X2 0.029
etaf 1297.123080 GPa.day, E1 31.674416 GPa, eta1 5.740832 GPa.day, E2 5.412257 GPa, eta2 185.458657 MPa.s
```

```
Fitting string N27 steps 3 to 12
A0 0.08161, K 2.957, G 0.035282, B 6.921815e-07, K1 0.00003304, X1 7.385, K2 0.00013180, X2 0.047
etaf 1444.707814 GPa.day, E1 30.266100 GPa, eta1 4.098140 GPa.day, E2 7.587298 GPa, eta2 162.017022 MPa.s
```

```
Fitting string N27 steps 3 to 13
A0 0.08164, K 2.957, G 0.035282, B 6.861717e-07, K1 0.00003296, X1 8.265, K2 0.00012806, X2 0.050
etaf 1457.361260 GPa.day, E1 30.337314 GPa, eta1 3.670798 GPa.day, E2 7.808695 GPa, eta2 157.558043 MPa.s
```

```
Fitting string N27 steps 3 to 14
A0 0.08163, K 2.957, G 0.035282, B 6.849870e-07, K1 0.00003346, X1 7.466, K2 0.00012883, X2 0.048
etaf 1459.881742 GPa.day, E1 29.884373 GPa, eta1 4.002527 GPa.day, E2 7.762302 GPa, eta2 161.801019 MPa.s
```

```
Fitting string N27 steps 3 to 15
A0 0.08167, K 2.957, G 0.035282, B 6.558682e-07, K1 0.00003565, X1 4.081, K2 0.00012460, X2 0.046
etaf 1524.696578 GPa.day, E1 28.053432 GPa, eta1 6.874691 GPa.day, E2 8.025462 GPa, eta2 174.921517 MPa.s
```

```
Fitting string N27 steps 3 to 16
A0 0.08236, K 2.957, G 0.035282, B 2.790737e-07, K1 0.00002034, X1 9.006, K2 0.00008145, X2 0.112
etaf 3583.282764 GPa.day, E1 49.173140 GPa, eta1 5.459803 GPa.day, E2 12.277096 GPa, eta2 109.209317 MPa.s
```

```
Fitting string N33 steps 3 to 5
A0 0.09874, K 2.946, G 0.035282, B 9.448516e-07, K1 0.00003548, X1 4.283, K2 0.00014418, X2 0.037
etaf 1058.367288 GPa.day, E1 28.186056 GPa, eta1 6.580613 GPa.day, E2 6.935590 GPa, eta2 187.569910 MPa.s
```

```
Fitting string N33 steps 3 to 6
A0 0.09865, K 2.946, G 0.035282, B 1.096447e-06, K1 0.00003369, X1 3.452, K2 0.00016192, X2 0.038
etaf 912.036602 GPa.day, E1 29.686492 GPa, eta1 8.600765 GPa.day, E2 6.175867 GPa, eta2 161.183357 MPa.s
```

```
Fitting string N28 steps 4 to 5
A0 0.05582, K 2.887, G 0.035282, B 1.667556e-06, K1 0.00007563, X1 14.029, K2 0.00008454, X2 0.435
etaf 599.679883 GPa.day, E1 13.222979 GPa, eta1 0.942549 GPa.day, E2 11.828797 GPa, eta2 27.172231 MPa.s
```

```
Fitting string N28 steps 8 to 9
A0 0.07923, K 2.887, G 0.035282, B 1.186260e-06, K1 0.00007750, X1 20.370, K2 0.00004673, X2 0.379
etaf 842.985644 GPa.day, E1 12.903292 GPa, eta1 0.633432 GPa.day, E2 21.398121 GPa, eta2 56.421567 MPa.s
```

```
Fitting string N28 steps 12 to 14
A0 0.10642, K 2.887, G 0.035282, B 3.785531e-07, K1 0.00003308, X1 9.328, K2 0.00006239, X2 0.126
etaf 2641.637632 GPa.day, E1 30.228740 GPa, eta1 3.240485 GPa.day, E2 16.028603 GPa, eta2 127.055168 MPa.s
```

```
Fitting string N28 steps 12 to 15
A0 0.10637, K 2.887, G 0.035282, B 3.893988e-07, K1 0.00002901, X1 9.698, K2 0.00006235, X2 0.149
etaf 2568.061389 GPa.day, E1 34.467275 GPa, eta1 3.554217 GPa.day, E2 16.039170 GPa, eta2 107.703654 MPa.s
```

## Plastic creep - strings N7 and N28¶

In [8]:

```
from matplotlib.patches import ConnectionPatch

def exp_limit_function(stress, A, D, S):
    return np.maximum(0.0,-A*np.expm1(D*(S-stress)))

def exp_rise_function(stress, A, D):
    return A*np.exp(D*stress)

def ln_exp_function(stress, A, D, S):
    return A*np.log(1-np.exp(-D*S)+np.exp(D*(stress-S)))

# data_array format: row=step(0..n); columns: stages A0 B A1 X1 A2 X2 A3 X3
# for A0 + B*t + A1(1-np.exp(-X1*t)) + A2(1-np.exp(-X2*t)) + A3(1-np.exp(-X3*t))

short_list = ['N7','N28']
show_last = False
show_BC_sum = False

# read fitted coefficients 
get_fitted_components(save_path+filename_start, short_list)  
    # reads fitted coefficients into sdict['fitted_values']
    # data_array format: row=step(0..n); columns: stages A0 B A1 X1 A2 X2 A3 X3
    # for A0 + B*t + A1*(1-np.exp(-X1*t)) + A2*(1-np.exp(-X2*t)) + A3*(1-np.exp(-X3*t))
    # creates sdict['fitted_steps'] arrays containing the elastic strain step at the start of each step response
    # also creates sdict['model_values'], same shape as sdict['fitted_values'], 
    # but containing E_el, eta_f, E_i, eta_i with units of GPa and GPa.day
for string in short_list:
    sdict = globals()[string]
    sdict['step_start_times'] = np.array([sdict['time_array'][sdict['step_starts'][i]] for i in range(sdict['test_steps'])])
    sdict['step_stop_times'] = np.array([sdict['time_array'][sdict['step_starts'][i+1]] for i in range(sdict['test_steps']-1)])
    sdict['step_stop_times'] = np.append(sdict['step_stop_times'], sdict['time_array'][-1])
    # step durations, calculated as period between successive step start times, except for last one (which has no following step)
    sdict['step_durations'] = sdict['step_stop_times'] - sdict['step_start_times']
    sdict['dashpot_strain'] = sdict['fitted_values'][:,2] * sdict['step_durations']
    
# subsets for N28 'exceeding' steps
N28_ex_steps = np.nonzero(N28['exceeding_steps'])
N28_ex_stress = N28['stress_n'][N28_ex_steps]
N28_ex_Bt = N28['dashpot_strain'][N28_ex_steps]
N28_ex_etaf = N28['model_values'][:,2][N28_ex_steps]
N28_ex_A1 = N28['fitted_values'][:,3][N28_ex_steps]
N28_ex_X1 = N28['fitted_values'][:,4][N28_ex_steps]
N28_ex_A2 = N28['fitted_values'][:,5][N28_ex_steps]
N28_ex_X2 = N28['fitted_values'][:,6][N28_ex_steps]
# add in amplitude values during reduced-tension episodes
N28_ex_Bt[2] = np.sum(N28['dashpot_strain'][2:5])
N28_ex_Bt[4] = np.sum(N28['dashpot_strain'][6:9])
N28_ex_Bt[6] = np.sum(N28['dashpot_strain'][10:15])
N28_ex_A1[2] = np.sum(N28['fitted_values'][2:5,3])
N28_ex_A1[4] = np.sum(N28['fitted_values'][6:9,3])
N28_ex_A1[6] = np.sum(N28['fitted_values'][10:15,3])
N28_ex_A2[2] = np.sum(N28['fitted_values'][2:5,5])
N28_ex_A2[4] = N28['fitted_values'][6,5] + N28['fitted_values'][8,5]
N28_ex_A2[6] = np.sum(N28['fitted_values'][10:15,5])

# Amplitude study
# ===============
print('\nAmplitude study')
print('===============')
xrange = np.arange(0,270)

# For string N7, these plots suggest 2 or 3 parts to the stretching behaviour: 
# an initial 'straightening' phase up to about step 4, covered by the K-V stage;
# a main stretching phase, covered by the dashpot for steps 2 to 4, and then by the K-V stage for steps 5 to 9;
# a possible further stretching (to breaking) phase, covered by the dashpot for steps 7 to 9
# String N28 appears to behave in a similar manner

# String N7
# =========
print('\nString N7:')
# N7 'straightening' phase
N7['sequence_A'] = np.zeros(10)
N7['sequence_A'][0] = N7['fitted_values'][0,3]
N7['sequence_A'][1] = N7['fitted_values'][1,3] + N7['fitted_values'][1,5]
N7['sequence_A'][2] = N7['fitted_values'][2,3]
N7['sequence_A'][3] = N7['fitted_values'][3,3]
N7['sequence_A'][4] = N7['fitted_values'][4,5]
# cummulative amplitude
N7['A_cumm_amp'] = np.array([sum(N7['sequence_A'][:i+1]) for i in range(len(N7['sequence_A']))])
# fit 1-exp function to string 7 amplitude data
popt_7a, pcov_7a = curve_fit(exp_limit_function, N7['stress_n'][:4], N7['A_cumm_amp'][:4], p0=[0.037,0.02,21], 
                             bounds=([0.03,0,10],[0.04,1,50]))
N7['A_cumm_fit'] = exp_limit_function(xrange, *popt_7a)
print('fitted 1-exp function (A D S):',popt_7a)
# N7 second stretching phase
N7['sequence_B'] = [N7['dashpot_strain'][i] for i in range(0,4)] + [N7['fitted_values'][:,3][i] for i in range(4,9)] + [0]
# cummulative amplitude
N7['B_cumm_amp'] = np.array([sum(N7['sequence_B'][:i+1]) for i in range(len(N7['sequence_B']))])
# fit ln-exp function to string 7 amplitude data
popt_7b, pcov_7b = curve_fit(ln_exp_function, N7['stress_n'][:9], N7['B_cumm_amp'][:9], p0=[0.0078,0.063,122], 
                             bounds=([0,0,0],[np.inf,np.inf,300]))
N7['B_cumm_fit'] = ln_exp_function(xrange, *popt_7b)
print('fitted ln_exp function',popt_7b)
# N7 third stretching phase
N7['C_cumm_amp'] = np.zeros(10)
for i in range(4,9):
    N7['C_cumm_amp'][i] = N7['C_cumm_amp'][i-1] + N7['dashpot_strain'][i] 
# fit ln-exp function to string 7 amplitude data
popt_7c, pcov_7c = curve_fit(ln_exp_function, N7['stress_n'][:9], N7['C_cumm_amp'][:9], p0=[0.0023,0.1,207], 
                             bounds=([0,0,0],[np.inf,np.inf,300]))
N7['C_cumm_fit'] = ln_exp_function(xrange, *popt_7c)
print('fitted ln_exp function',popt_7c)
# N7 sum of second and third stretching phases
N7['BC_cumm_amp'] = N7['B_cumm_amp'] + N7['C_cumm_amp']
# fit ln-exp function
popt_7bc, pcov_7bc = curve_fit(ln_exp_function, N7['stress_n'][:9], N7['BC_cumm_amp'][:9], p0=[0.016,0.04,142], 
                               bounds=([0,0,0],[np.inf,np.inf,300]))
N7['BC_cumm_fit'] = ln_exp_function(xrange, *popt_7bc)
print('fitted ln_exp function',popt_7bc)
# N7 overall cummulative amplitude
N7['ABC_cumm_amp'] = N7['A_cumm_amp'] + N7['B_cumm_amp'] + N7['C_cumm_amp']
N7['ABC_cumm_fit'] = N7['A_cumm_fit'] + N7['B_cumm_fit'] + N7['C_cumm_fit']
#                          
# String N28
# ==========
print('\nString N28:')
# N28 'straightening' phase
N28['sequence_A'] = np.zeros(10)
N28['sequence_A'][0] = N28['fitted_values'][0,3] + N28['fitted_values'][0,5]
N28['sequence_A'][1] = N28['fitted_values'][1,3] + N28['fitted_values'][1,5]
N28['sequence_A'][2] = np.sum(N28['fitted_values'][2:5,3]) + np.sum(N28['fitted_values'][2:5,5])
N28['sequence_A'][3] = N28['fitted_values'][5,3] + N28['fitted_values'][5,5]
N28['sequence_A'][4] = N28['fitted_values'][6,5] + N28['fitted_values'][8,5]
N28['sequence_A'][5] = N28['fitted_values'][9,5]
N28['sequence_A'][6] = np.sum(N28['fitted_values'][10:15,5])
N28['sequence_A'][7] = N28['fitted_values'][15,5]
# cummulative amplitude
N28['A_cumm_amp'] = np.array([sum(N28['sequence_A'][:i+1]) for i in range(len(N28['sequence_A']))])
# fit 1-exp function to string 7 amplitude data
popt_28a, pcov_28a = curve_fit(exp_limit_function, N28_ex_stress[:8], N28['A_cumm_amp'][:8], p0=[0.072,0.015,16], 
                               bounds=([0.03,0,0],[0.15,1,50]))
N28['A_cumm_fit'] = exp_limit_function(xrange, *popt_28a)
print('fitted 1-exp function (A D S):',popt_28a)
# N28 second stretching phase
N28['sequence_B'] = np.zeros(10)
N28['sequence_B'][1] = N28['dashpot_strain'][1]
N28['sequence_B'][2] = np.sum(N28['dashpot_strain'][2:5])
N28['sequence_B'][3] = N28['dashpot_strain'][5]
N28['sequence_B'][4] = np.sum(N28['fitted_values'][6:9,3])
N28['sequence_B'][5] = N28['fitted_values'][9,3]
N28['sequence_B'][6] = np.sum(N28['fitted_values'][10:15,3])
N28['sequence_B'][7] = N28['fitted_values'][15,3]
N28['sequence_B'][8] = N28['fitted_values'][16,3]
# cummulative amplitude
N28['B_cumm_amp'] = np.array([sum(N28['sequence_B'][:i+1]) for i in range(len(N28['sequence_B']))])
# fit ln-exp function to string 28 amplitude data
popt_28b, pcov_28b = curve_fit(ln_exp_function, N28_ex_stress[:9], N28['B_cumm_amp'][:9], p0=[0.017,0.029,136], 
                               bounds=([0,0,0],[np.inf,np.inf,300]))
N28['B_cumm_fit'] = ln_exp_function(xrange, *popt_28b)
print('fitted ln_exp function',popt_28b)
# N28 third stretching phase
N28['sequence_C'] = np.zeros(10)
N28['sequence_C'][4] = np.sum(N28['dashpot_strain'][6:9])
N28['sequence_C'][5] = N28['dashpot_strain'][9]
N28['sequence_C'][6] = np.sum(N28['dashpot_strain'][10:15])
N28['sequence_C'][7] = N28['dashpot_strain'][15]
N28['sequence_C'][8] = N28['dashpot_strain'][16]
# cummulative amplitude
N28['C_cumm_amp'] = np.array([sum(N28['sequence_C'][:i+1]) for i in range(len(N28['sequence_B']))])
# fit ln-exp function to string 28 amplitude data
popt_28c, pcov_28c = curve_fit(ln_exp_function, N28_ex_stress[4:9], N28['C_cumm_amp'][4:9], p0=[8,0.034,470],
                               bounds=([0,0,0],[np.inf,np.inf,500]))
#4.53424037e+00 3.45011211e-02 4.51329168e+02
N28['C_cumm_fit'] = ln_exp_function(xrange, *popt_28c)
print('fitted ln_exp function',popt_28c)
# N28 sum of second and third stretching phases
N28['BC_cumm_amp'] = N28['B_cumm_amp'] + N28['C_cumm_amp']
# fit ln-exp function
popt_28bc, pcov_28bc = curve_fit(ln_exp_function, N28_ex_stress[:9], N28['BC_cumm_amp'][:9], p0=[0.02,0.027,146], 
                                 bounds=([0,0,0],[np.inf,np.inf,300]))
N28['BC_cumm_fit'] = ln_exp_function(xrange, *popt_28bc)
print('fitted ln_exp function',popt_28bc)
# N28 overall cummulative amplitude
N28['ABC_cumm_amp'] = N28['A_cumm_amp'] + N28['B_cumm_amp'] + N28['C_cumm_amp']
N28['ABC_cumm_fit'] = N28['A_cumm_fit'] + N28['B_cumm_fit'] + N28['C_cumm_fit']


# Plot data
# =========
last = 10 if(show_last) else 9
fig=plt.figure(figsize=fig_size_4)
ax1=fig.add_subplot(421)
ax2=fig.add_subplot(422)
ax3=fig.add_subplot(423)
ax4=fig.add_subplot(424)
ax5=fig.add_subplot(425)
ax6=fig.add_subplot(426)
ax7=fig.add_subplot(427)
ax8=fig.add_subplot(428)
# zero axes
x2 = 300 if(show_last) else 270
ax1.plot([0,x2],[0,0],'k--',lw=0.75)
ax2.plot([0,x2],[0,0],'k--',lw=0.75)
ax3.plot([0,x2],[0,0],'k--',lw=0.75)
ax4.plot([0,x2],[0,0],'k--',lw=0.75)
ax5.plot([0,x2],[0,0],'k--',lw=0.75)
ax6.plot([0,x2],[0,0],'k--',lw=0.75)
ax7.plot([0,x2],[0,0],'k--',lw=0.75)
ax8.plot([0,x2],[0,0],'k--',lw=0.75)
# N7 step amplitudes
ax1.plot([N7['stress_n'][1],N7['stress_n'][1]], [N7['sequence_A'][1],N7['fitted_values'][1,5]], 'k:', lw=1)
ax1.plot(N7['stress_n'][:last], N7['dashpot_strain'][:last], color=N7['pen'], ls='none', marker='*')
ax1.plot(N7['stress_n'][:last], N7['fitted_values'][:last,3], color=N7['pen'], ls='none', marker='^', ms=4)
ax1.plot(N7['stress_n'][:last], N7['fitted_values'][:last,5], color=N7['pen'], ls='none', marker='v', ms=4)
ax1.plot(N7['stress_n'][:5], N7['sequence_A'][:5], color=N7['pen'], ls='-', lw=0.5, marker='.', ms=4)
ax1.plot(N7['stress_n'][:9], N7['sequence_B'][:9], color=N7['pen'], ls='-', lw=0.5, marker='.', ms=4)
ax1.plot(N7['stress_n'][4:9], N7['dashpot_strain'][4:9], color=N7['pen'], ls='-', lw=0.5)
# N28 step amplitudes
ax2.plot([N28_ex_stress[0],N28_ex_stress[0]], [N28['sequence_A'][0],N28_ex_A2[0]], 'k:', lw=1)
ax2.plot([N28_ex_stress[1],N28_ex_stress[1]], [N28['sequence_A'][1],N28_ex_A1[1]], 'k:', lw=1)
ax2.plot([N28_ex_stress[2],N28_ex_stress[2]], [N28['sequence_A'][2],N28_ex_A2[2]], 'k:', lw=1)
ax2.plot([N28_ex_stress[3],N28_ex_stress[3]], [N28['sequence_A'][3],N28_ex_A2[3]], 'k:', lw=1)
ax2.plot(N28_ex_stress[:last], N28_ex_Bt[:last], color=N28['pen'], ls='none', marker='*')
ax2.plot(N28_ex_stress[:last], N28_ex_A1[:last], color=N28['pen'], ls='none', marker='^', ms=4)
ax2.plot(N28_ex_stress[:last], N28_ex_A2[:last], color=N28['pen'], ls='none', marker='v', ms=4)
ax2.plot(N28_ex_stress[:8], N28['sequence_A'][:8], color=N28['pen'], ls='-', lw=0.5, marker='.', ms=4)
ax2.plot(N28_ex_stress[:9], N28['sequence_B'][:9], color=N28['pen'], ls='-', lw=0.5, marker='.', ms=4)
ax2.plot(N28_ex_stress[4:9], N28['sequence_C'][4:9], color=N28['pen'], ls='-', lw=0.5, marker='.', ms=4)
# N7 cummulative amplitude
# fitted functions
ax3.plot(xrange,N7['A_cumm_fit'],'k--',lw=0.75)
ax3.plot(xrange,N7['B_cumm_fit'],'k--',lw=0.75)
ax3.plot(xrange,N7['C_cumm_fit'],'k--',lw=0.75)
ax3.plot(xrange,N7['ABC_cumm_fit'],'k--',lw=0.75)
if(show_BC_sum):
    ax3.plot(xrange,N7['BC_cumm_fit'],'k--',lw=0.75)
    ax3.plot(N7['stress_n'][:9], N7['BC_cumm_amp'][:9], color=N7['pen'], ls='none', marker='+')
# data
ax3.plot(N7['stress_n'][:5], N7['A_cumm_amp'][:5], color=N7['pen'], ls='none', marker='o', ms=3)
ax3.plot(N7['stress_n'][:9], N7['B_cumm_amp'][:9], color=N7['pen'], ls='none', marker='o', ms=3)
ax3.plot(N7['stress_n'][4:9], N7['C_cumm_amp'][4:9], color=N7['pen'], ls='none', marker='o', ms=3)
ax3.plot(N7['stress_n'][:9], N7['ABC_cumm_amp'][:9], color=N7['pen'], ls='none', marker='x')
# N28 cummulative amplitude
# fitted functions
ax4.plot(xrange,N28['A_cumm_fit'],'k--',lw=0.75)
ax4.plot(xrange,N28['B_cumm_fit'],'k--',lw=0.75)
ax4.plot(xrange,N28['C_cumm_fit'],'k--',lw=0.75)
ax4.plot(xrange,N28['ABC_cumm_fit'],'k--',lw=0.75)
if(show_BC_sum):
    ax4.plot(xrange,N28['BC_cumm_fit'],'k--',lw=0.75)
    ax4.plot(N28_ex_stress[:9], N28['BC_cumm_amp'][:9], color=N28['pen'], ls='none', marker='+')
# data
ax4.plot(N28_ex_stress[:8], N28['A_cumm_amp'][:8], color=N28['pen'], ls='none', marker='o', ms=3)
ax4.plot(N28_ex_stress[:9], N28['B_cumm_amp'][:9], color=N28['pen'], ls='none', marker='o', ms=3)
ax4.plot(N28_ex_stress[4:9], N28['C_cumm_amp'][4:9], color=N28['pen'], ls='none', marker='o', ms=3)
ax4.plot(N28_ex_stress[:9], N28['ABC_cumm_amp'][:9], color=N28['pen'], ls='none', marker='x')
# strain rate / stress for series dashpot
ax5.plot(N7['stress_n'][:last], 1/N7['model_values'][:last,2], color=N7['pen'], ls='none', marker='*')
ax5.plot(N7['stress_n'][:4], 1/N7['model_values'][:4,2], color=N7['pen'], ls='-', lw=0.5)
ax5.plot(N7['stress_n'][4:9], 1/N7['model_values'][4:9,2], color=N7['pen'], ls='-', lw=0.5)
ax6.plot(N28_ex_stress[:last], 1/N28_ex_etaf[:last], color=N28['pen'], ls='none', marker='*')
ax6.plot(N28_ex_stress[:4], 1/N28_ex_etaf[:4], color=N28['pen'], ls='-', lw=0.5)
ax6.plot(N28_ex_stress[4:9], 1/N28_ex_etaf[4:9], color=N28['pen'], ls='-', lw=0.5)
# N7 time coefficients X_i
N7_plot_sequence_A = np.append(N7['fitted_values'][:4,4], N7['fitted_values'][4,6])
N7_plot_sequence_A[1] = np.sqrt(N7['fitted_values'][1,4] * N7['fitted_values'][1,6])
ax7.plot([N7['stress_n'][1],N7['stress_n'][1]], [N7['fitted_values'][1,4],N7['fitted_values'][1,6]], 'k:', lw=1)
ax7.plot(N7['stress_n'][:last], N7['fitted_values'][:last,4], color=N7['pen'], ls='none', marker='^', ms=4)
ax7.plot(N7['stress_n'][:last], N7['fitted_values'][:last,6], color=N7['pen'], ls='none', marker='v', ms=4)
ax7.plot(N7['stress_n'][:5], N7_plot_sequence_A, color=N7['pen'], ls='-', lw=0.5, marker='.', ms=4)
ax7.plot(N7['stress_n'][4:9], N7['fitted_values'][4:9,4], color=N7['pen'], ls='-', lw=0.5, marker='.', ms=4)
# N28 time coefficients X_i
N28_plot_sequence_A = np.copy(N28_ex_X2[:9])
for i in range(4):
    N28_plot_sequence_A[i] = np.sqrt(N28_ex_X1[i] * N28_ex_X2[i])
    ax8.plot([N28_ex_stress[i],N28_ex_stress[i]], [N28_ex_X1[i],N28_ex_X2[i]], 'k:', lw=1)
ax8.plot(N28_ex_stress[:last], N28_ex_X1[:last], color=N28['pen'], ls='none', marker='^', ms=4)
ax8.plot(N28_ex_stress[:last], N28_ex_X2[:last], color=N28['pen'], ls='none', marker='v', ms=4)
ax8.plot(N28_ex_stress[:9], N28_plot_sequence_A, color=N28['pen'], ls='-', lw=0.5, marker='.', ms=4)
ax8.plot(N28_ex_stress[4:9], N28_ex_X1[4:9], color=N28['pen'], ls='-', lw=0.5, marker='.', ms=4)
# axes
x2 = 310 if(show_last) else 280
ax1.axis([-10,x2,-0.002,0.022])
ax2.axis([-10,x2,-0.002,0.022])
#ax3.axis([-10,x2,-0.007,0.079])
#ax4.axis([-10,x2,-0.007,0.079])
ax3.axis([-10,x2,-0.01,0.14])
ax4.axis([-10,x2,-0.01,0.14])
ax5.axis([-10,x2,-0.0005,0.0055])
ax6.axis([-10,x2,-0.0005,0.0055])
ax7.axis([-10,x2,0.04,40])
ax8.axis([-10,x2,0.04,40])
ax1.set_xlabel('Applied stress (MPa)')
ax2.set_xlabel('Applied stress (MPa)')
ax3.set_xlabel('Applied stress (MPa)')
ax4.set_xlabel('Applied stress (MPa)')
ax5.set_xlabel('Applied stress (MPa)')
ax6.set_xlabel('Applied stress (MPa)')
ax7.set_xlabel('Applied stress (MPa)')
ax8.set_xlabel('Applied stress (MPa)')
ax1.set_ylabel('Step amplitude components')
ax2.set_ylabel('Step amplitude components')
ax3.set_ylabel('Cummulative amplitude')
ax4.set_ylabel('Cummulative amplitude')
ax5.set_ylabel('$1/\eta_f$ (1/GPa.day)')
ax6.set_ylabel('$1/\eta_f$ (1/GPa.day)')
ax7.set_ylabel('Time coeff. $X_i$ (1/day)')
ax8.set_ylabel('Time coeff. $X_i$ (1/day)')
ax7.set_yscale('log')
ax8.set_yscale('log')
y_axis = y_values = [0.1,1,10]
ax7.set_yticks(y_axis, y_values)
ax8.set_yticks(y_axis, y_values)
ax1.set_title("String N7")
ax2.set_title("String N28")

fig.tight_layout()

# connecting lines
# N7
x1 = N7['stress_n'][3]
x2 = N7['stress_n'][4]
y1 = 1/N7['model_values'][3,2]
y2 = N7['fitted_values'][4,4]
xy1 = (x1,y1)
xy2 = (x2,y2)
con = ConnectionPatch(xyA=xy1, xyB=xy2, coordsA="data", coordsB="data", axesA=ax5, axesB=ax7, 
                      color=N7['pen'], linestyle='dotted', linewidth=1)
ax7.add_artist(con)
ax5.plot(x1, y1, color=N7['pen'], marker='.', ms=4)
ax7.plot(x2, y2, color=N7['pen'], marker='.', ms=4)
# N28
x1 = N28_ex_stress[3]
x2 = N28_ex_stress[4]
y1 = 1/N28_ex_etaf[3]
y2 = N28_ex_X1[4]
xy1 = (x1,y1)
xy2 = (x2,y2)
con = ConnectionPatch(xyA=xy1, xyB=xy2, coordsA="data", coordsB="data", axesA=ax6, axesB=ax8, 
                      color=N28['pen'], linestyle='dotted', linewidth=1)
ax8.add_artist(con)
ax6.plot(x1, y1, color=N28['pen'], marker='.', ms=4)
ax8.plot(x2, y2, color=N28['pen'], marker='.', ms=4)

ax1.text(0.05, 0.85,'(a)', transform=ax1.transAxes)
ax2.text(0.05, 0.85,'(b)', transform=ax2.transAxes)
ax3.text(0.05, 0.85,'(c)', transform=ax3.transAxes)
ax4.text(0.05, 0.85,'(d)', transform=ax4.transAxes)
ax5.text(0.05, 0.85,'(e)', transform=ax5.transAxes)
ax6.text(0.05, 0.85,'(f)', transform=ax6.transAxes)
ax7.text(0.05, 0.85,'(g)', transform=ax7.transAxes)
ax8.text(0.05, 0.85,'(h)', transform=ax8.transAxes)
savefile = save_path + "plastic_creep_study." + save_format
print("\n%s" % (savefile))
#fig.savefig(savefile, format=save_format, dpi=1000)
plt.show()
```

```
Amplitude study
===============

String N7:
fitted 1-exp function (A D S): [3.65833773e-02 2.09136435e-02 2.12427590e+01]
fitted ln_exp function [8.07575711e-03 6.11319626e-02 1.22027782e+02]
fitted ln_exp function [2.32833940e-03 1.00050601e-01 2.07050296e+02]
fitted ln_exp function [1.63883748e-02 4.09121533e-02 1.41976700e+02]

String N28:
fitted 1-exp function (A D S): [7.22816997e-02 1.53634195e-02 1.68722479e+01]
fitted ln_exp function [1.66804736e-02 2.93116037e-02 1.35792279e+02]
fitted ln_exp function [1.35051315e+01 3.44950650e-02 4.83012883e+02]
fitted ln_exp function [2.07745642e-02 2.72689880e-02 1.45929425e+02]

./plastic_creep_study.eps
```

### Multi-step fitting for plastic creep - strings N7 and N28¶

In [9]:

```
def test_plastic_creep_function(time_array, A0, B, A1, D1, S1, P1, Q1, A2, D2, S2, X2):
    sdict = globals()[fit_string]
    el_strain_array = np.zeros_like(time_array)
    creep_array_1 = np.zeros_like(time_array)
    creep_array_2 = np.zeros_like(time_array)
    tail_array = np.zeros_like(time_array)
    KV1_amplitude = np.maximum(0.0,-A1*np.expm1(D1*(S1-sdict['stress_n'])))
    KV2_amplitude = A2*np.log(1-np.exp(-D2*S2)+np.exp(D2*(sdict['stress_n']-S2)))
    inv_tau1 = P1+sdict['stress_n']*Q1
    inv_tau2 = X2
    tail_rate = B * sdict['stress_n']
    for step in range(1,fit_stop_step+1,1):  # step numbers to fit
        start_index = sdict['step_starts'][step-1] + 1  # don't include first point of step (at previous stress level)
        stop_index = sdict['step_starts'][step]
        stop_index += 1 if(step < sdict['test_steps']) else 0
            # include first point of next step (at current stress level), except for very last step
        neg_time_sub_array = time_array[start_index-1] - time_array[start_index:stop_index] # negative time since step start
        # elastic strain component
        el_strain_array[start_index:stop_index] = sdict['el_strain_est'][step-1]
        # creep component 1
        base = creep_array_1[start_index-1] # last point calculated = first point of current step (0 at start of first step)
        target = KV1_amplitude[step-1]
        creep_array_1[start_index:stop_index] = base + (base - target) * np.expm1(inv_tau1[step-1] * neg_time_sub_array)
        # creep component 2
        base = creep_array_2[start_index-1] # last point calculated = first point of current step (0 at start of first step)
        target = KV2_amplitude[step-1]
        creep_array_2[start_index:stop_index] = base + (base - target) * np.expm1(inv_tau2 * neg_time_sub_array)
        # final dashpot component
        tail_array[start_index:stop_index] = tail_array[start_index-1] - tail_rate[step-1] * neg_time_sub_array
    return A0 + el_strain_array + creep_array_1 + creep_array_2 + tail_array

def test_plastic_creep_function_noB(time_array, A0, A1, D1, S1, P1, Q1, A2, D2, S2, X2):
    sdict = globals()[fit_string]
    el_strain_array = np.zeros_like(time_array)
    creep_array_1 = np.zeros_like(time_array)
    creep_array_2 = np.zeros_like(time_array)
    KV1_amplitude = np.maximum(0.0,-A1*np.expm1(D1*(S1-sdict['stress_n'])))
    KV2_amplitude = A2*np.log(1-np.exp(-D2*S2)+np.exp(D2*(sdict['stress_n']-S2)))
    inv_tau1 = P1+sdict['stress_n']*Q1
    inv_tau2 = X2
    for step in range(1,fit_stop_step+1,1):  # step numbers to fit
        start_index = sdict['step_starts'][step-1] + 1  # don't include first point of step (at previous stress level)
        stop_index = sdict['step_starts'][step]
        stop_index += 1 if(step < sdict['test_steps']) else 0
            # include first point of next step (at current stress level), except for very last step
        neg_time_sub_array = time_array[start_index-1] - time_array[start_index:stop_index] # negative time since step start
        # elastic strain component
        el_strain_array[start_index:stop_index] = sdict['el_strain_est'][step-1]
        # creep component 1
        base = creep_array_1[start_index-1] # last point calculated = first point of current step (0 at start of first step)
        target = KV1_amplitude[step-1]
        creep_array_1[start_index:stop_index] = base + (base - target) * np.expm1(inv_tau1[step-1] * neg_time_sub_array)
        # creep component 2
        base = creep_array_2[start_index-1] # last point calculated = first point of current step (0 at start of first step)
        target = KV2_amplitude[step-1]
        creep_array_2[start_index:stop_index] = base + (base - target) * np.expm1(inv_tau2 * neg_time_sub_array)
    return A0 + el_strain_array + creep_array_1 + creep_array_2

def multi_step_fit(fit_string_value, stop_step, fit_last, incl_dashpot):
    global fit_string
    global fit_stop_step
    global fitted_stop_step
    global time_sub_array
    global strain_sub_array
    global fitted_curve
    global el_strain_array
    global creep_array_1
    global creep_array_2
    global tail_array
    global check_array
    #
    fit_string = fit_string_value
    sdict = globals()[fit_string]
    fit_stop_step = stop_step - (1 if(stop_step == sdict['test_steps'] and not fit_last) else 0)  
        # don't fit to last step where string broke unless fit_last == True
    print('\nFitting string', fit_string, 'steps 1 to', fit_stop_step)
    start_index = sdict['step_starts'][0]
    stop_index = sdict['step_starts'][fit_stop_step]
    stop_index += 1 if(fit_stop_step < sdict['test_steps']) else 0
    time_sub_array = sdict['time_array'][start_index:stop_index]
    strain_sub_array = sdict['strain_array'][start_index:stop_index]
    time_for_fitting = time_sub_array - sdict['time_array'][start_index]
    #
    # curve fitting
    if(incl_dashpot):
        popt, pcov = curve_fit(test_plastic_creep_function, time_for_fitting, strain_sub_array, p0 = sdict['p0'],
                               bounds=([-0.5,0,0,0,0,0,0,0,0,0,-10],[0.5,0.001,1,1,50,1,1,1,1,300,50]))
    else:
        popt, pcov = curve_fit(test_plastic_creep_function_noB, time_for_fitting, strain_sub_array, p0 = sdict['p0_noB'],
                               bounds=([-0.5,0,0,0,0,0,0,0,0,-10],[0.5,1,1,50,1,1,1,1,300,50]))
    print(popt)
    fitted_stop_step = fit_stop_step
    #
    # reconstruct fitted components
    print('\nPlotting string', fit_string, 'steps 1 to', stop_step)
    stop_index = sdict['step_starts'][stop_step]
    stop_index += 1 if(stop_step < sdict['test_steps']) else 0
    time_sub_array = sdict['time_array'][start_index:stop_index]
    strain_sub_array = sdict['strain_array'][start_index:stop_index]
    time_for_fitting = time_sub_array - sdict['time_array'][start_index]
    # evaluate fitted curve over full plot range
    fit_stop_step = stop_step
    if(incl_dashpot):
        fitted_curve = test_plastic_creep_function(time_for_fitting, *popt)
        Bbit = 1
        tail_rate = popt[1] * sdict['stress_n']
    else:
        fitted_curve = test_plastic_creep_function_noB(time_for_fitting, *popt)
        Bbit = 0
    # components are: A0, B, A1, D1, S1, P1, Q1, A2, D2, S2, X2, but without B if no series dashpot
    el_strain_array = np.zeros_like(time_for_fitting)
    creep_array_1 = np.zeros_like(time_for_fitting)
    creep_array_2 = np.zeros_like(time_for_fitting)
    tail_array = np.zeros_like(time_for_fitting)
    KV1_amplitude = np.maximum(0.0,-popt[1+Bbit]*np.expm1(popt[2+Bbit]*(popt[3+Bbit]-sdict['stress_n'])))
    inv_tau1 = popt[4+Bbit]+sdict['stress_n']*popt[5+Bbit]
    KV2_amplitude = popt[6+Bbit]*np.log(1-np.exp(-popt[7+Bbit]*popt[8+Bbit])
                                        +np.exp(popt[7+Bbit]*(sdict['stress_n']-popt[8+Bbit])))
    inv_tau2 = popt[9+Bbit]
    for step in range(1,stop_step+1,1):  # step numbers to fit
        start_index = sdict['step_starts'][step-1] + 1  # don't include first point of step (at previous stress level)
        stop_index = sdict['step_starts'][step]
        stop_index += 1 if(step < sdict['test_steps']) else 0
            # include first point of next step (at current stress level), except for very last step
        neg_time_sub_array = time_for_fitting[start_index-1] - time_for_fitting[start_index:stop_index] # negative time since step start
        el_strain_array[start_index:stop_index] = sdict['el_strain_est'][step-1]
        # creep component 1
        base = creep_array_1[start_index-1] # last point calculated = first point of current step (0 at start of first step)
        target = KV1_amplitude[step-1]
        creep_array_1[start_index:stop_index] = base + (base - target) * np.expm1(inv_tau1[step-1] * neg_time_sub_array)
        # creep component 2
        base = creep_array_2[start_index-1] # last point calculated = first point of current step (0 at start of first step)
        target = KV2_amplitude[step-1]
        creep_array_2[start_index:stop_index] = base + (base - target) * np.expm1(inv_tau2 * neg_time_sub_array)
        if(incl_dashpot):
            # final dashpot component
            tail_array[start_index:stop_index] = tail_array[start_index-1] - tail_rate[step-1] * neg_time_sub_array
    check_array = popt[0] + el_strain_array + creep_array_1 + creep_array_2 + tail_array
    return popt

def multi_step_fit_plot_ax(ax, fit_string_value, stop_step, fit_last, incl_dashpot):
    # run curve fitting
    popt = multi_step_fit(fit_string_value, stop_step, fit_last, incl_dashpot)
    # initial data
    ax.plot(time_sub_array, strain_sub_array, 'k-', label='strain data')
    ax.plot(time_sub_array[1:], fitted_curve[1:], 'r--', label='fitted curve')
#    ax.plot(time_sub_array[1:], check_array[1:], 'b--', label='check curve')
    # plot component parts of fitted curve
    plot_offset = popt[0]+el_strain_array[1]  # el_strain_array[0] = 0 so need later value
#    ax.plot(time_sub_array, [popt[0] for i in range(len(time_sub_array))], 'k:', label='offset')
#    ax.plot(time_sub_array, popt[0]+el_strain_array, 'c:', label='spring')
    ax.plot(time_sub_array, plot_offset+creep_array_1, 'k:', label='1st K-V stage')
    ax.plot(time_sub_array, plot_offset+creep_array_2, 'r:', label='2nd K-V stage')
    if(incl_dashpot):
        ax.plot(time_sub_array, plot_offset+tail_array, 'g:', label='dashpot')
        ax.plot(time_sub_array, plot_offset+creep_array_2+tail_array, ':', color='orange', label='2nd K-V stage + dashpot')
    ax.set_title('String ' + str(fit_string_value) + ', fitted steps 1 to ' + str(fitted_stop_step))
    ax.set_xlabel('Time (days)')
    ax.set_ylabel('Strain')
    ax.legend(loc='best', prop={'size':10}, framealpha=1)

def multi_step_fit_plot(fit_string_value, stop_step, fit_last, incl_dashpot, save_plot):
    # plot data    
    fig=plt.figure(figsize=(8,5))
    ax1=fig.add_subplot(111)
    multi_step_fit_plot_ax(ax1, fit_string_value, stop_step, fit_last, incl_dashpot)
    fig.tight_layout()
    if(save_plot):
        savefile = save_path + "plastic_creep_multi_step_" + fit_string_value + '_1_to_' \
                    + str(stop_step) + '.' + save_format
        print("\n%s" % (savefile))
        fig.savefig(savefile, format=save_format, dpi=1000)
    plt.show()

    
N7['p0'] = [0,0,0.028,0.028,23,0,0.021,0.047,0.018,166,0.65]
N7['p0_noB'] = [0,0.028,0.028,23,0,0.021,0.047,0.018,166,0.65]


fig=plt.figure(figsize=fig_size_2)
ax1=fig.add_subplot(121)
ax2=fig.add_subplot(122)
multi_step_fit_plot_ax(ax1, 'N7', 10, False, False)
multi_step_fit_plot_ax(ax2, 'N7', 10, True, False)
fig.tight_layout()
ax1.text(0.9, 0.07,'(a)', transform=ax1.transAxes)
ax2.text(0.9, 0.07,'(b)', transform=ax2.transAxes)
savefile = save_path + "plastic_creep_multi_step_N7." + save_format
print("\n%s" % (savefile))
#fig.savefig(savefile, format=save_format, dpi=1000)
plt.show()


# individual plots
# ================
# N7
multi_step_fit_plot('N7', 8, False, True, False)
multi_step_fit_plot('N7', 9, False, True, False)
multi_step_fit_plot('N7', 10, False, True, False)
multi_step_fit_plot('N7', 10, False, False, False)
multi_step_fit_plot('N7', 10, True, False, False)
```

```
Fitting string N7 steps 1 to 9
[-6.71738980e-03  2.96128498e-02  2.72070313e-02  2.31504489e+01
  1.89442461e-34  1.85243212e-02  3.90465325e-02  2.12663034e-02
  1.58013783e+02  4.66040654e-01]

Plotting string N7 steps 1 to 10

Fitting string N7 steps 1 to 10
[-6.65124184e-03  2.11190069e-02  3.44332576e-02  2.59417046e+01
  4.42956045e-38  2.70938944e-02  2.11262375e-01  1.06992536e-02
  3.00000000e+02  4.35316412e-01]

Plotting string N7 steps 1 to 10

./plastic_creep_multi_step_N7.eps
```

```
Fitting string N7 steps 1 to 8
[-6.49336425e-03  3.05551413e-06  3.05470106e-02  2.51592352e-02
  2.26488758e+01  3.27400578e-27  1.95225234e-02  1.39845864e-02
  3.42237380e-02  1.47740552e+02  7.00882078e-01]

Plotting string N7 steps 1 to 8
```

```
Fitting string N7 steps 1 to 9
[-6.55762626e-03  3.03500699e-06  2.82664702e-02  2.76019338e-02
  2.30565081e+01  4.06839413e-28  2.01718407e-02  2.10815319e-02
  2.64324362e-02  1.57853814e+02  6.82486044e-01]

Plotting string N7 steps 1 to 9
```

```
Fitting string N7 steps 1 to 9
[-6.55762626e-03  3.03500699e-06  2.82664702e-02  2.76019338e-02
  2.30565081e+01  4.06839413e-28  2.01718407e-02  2.10815319e-02
  2.64324362e-02  1.57853814e+02  6.82486044e-01]

Plotting string N7 steps 1 to 10
```

```
Fitting string N7 steps 1 to 9
[-6.71738980e-03  2.96128498e-02  2.72070313e-02  2.31504489e+01
  1.89442461e-34  1.85243212e-02  3.90465325e-02  2.12663034e-02
  1.58013783e+02  4.66040654e-01]

Plotting string N7 steps 1 to 10
```

```
Fitting string N7 steps 1 to 10
[-6.65124184e-03  2.11190069e-02  3.44332576e-02  2.59417046e+01
  4.42956045e-38  2.70938944e-02  2.11262375e-01  1.06992536e-02
  3.00000000e+02  4.35316412e-01]

Plotting string N7 steps 1 to 10
```

### Multi-step fitting for N28¶

### String N28 with recoverable creep episodes removed¶

In [10]:

```
short_list = ['N28']

# read fitted coefficients 
get_fitted_components(save_path+filename_start, short_list)  
    # reads fitted coefficients into sdict['fitted_values']
    # data_array format: row=step(0..n); columns: stages A0 B A1 X1 A2 X2 A3 X3
    # for A0 + B*t + A1*(1-np.exp(-X1*t)) + A2*(1-np.exp(-X2*t)) + A3*(1-np.exp(-X3*t))
    # creates sdict['fitted_steps'] arrays containing the elastic strain step at the start of each step response
    # also creates sdict['model_values'], same shape as sdict['fitted_values'], 
    # but containing E_el, eta_f, E_i, eta_i with units of GPa and GPa.day

N28m = copy.deepcopy(N28)
fitted_values = np.copy(N28m['fitted_values'])

for stepm1 in range(2,15):  # no changes needed before step 4 or after step 15, start from step 3 to set ref_time etc
    if(N28m['exceeding_steps'][stepm1] == 1):
        # exceeding step so just update ref_time to start of this step
        ref_step = stepm1
        print(ref_step, fitted_values[ref_step,2])
        ref_time = N28m['time_array'][N28m['step_starts'][stepm1]]  # start of this step
        # calculate dashpot contribution up to start of next step
        ref_dashpot = fitted_values[ref_step,2] * (N28m['time_array'][N28m['step_starts'][stepm1+1]] - ref_time)
        # zero dashpot coefficient, so no contribution beyond this step
        fitted_values[ref_step,2] = 0
        ref_stress = N28m['stress_n'][stepm1]
        ref_el_strain_est = N28m['el_strain_est'][stepm1]
    else:
        # non-exceeding step => needs adjusting
        start_index = N28m['step_starts'][stepm1]
        stop_index = N28m['step_starts'][stepm1+1] + 1  # include first point of next step (always finishing before last step)
        time_sub_array = N28m['time_array'][start_index:stop_index] - ref_time
        tail_array = fit_func(time_sub_array, fitted_values[ref_step]) + ref_dashpot # no dashpot contribution beyond ref_step
        N28m['strain_array'][start_index:stop_index] = tail_array
        N28m['stress_n'][stepm1] = ref_stress
        N28m['stress_n_step'][stepm1] = 0
        N28m['el_strain_est'][stepm1] = ref_el_strain_est

fig=plt.figure(figsize=(8,5))
ax1=fig.add_subplot(111)
ax1.plot(N28['time_array'], N28['strain_array'], '-', color=N28['pen'])
ax1.plot(N28m['time_array'], N28m['strain_array'], '--', color='lime')
fig.tight_layout()
plt.show()
```

```
2 0.0002453996041203144
5 0.0002947417730064701
6 3.065488421742923e-15
9 4.356142190128786e-24
10 4.154270606459647e-21
```

### Multi-step fitting for string N28 with and without recoverable creep episodes removed¶

In [11]:

```
def test_combined_creep_function_28m(time_array, A0, A1, D1, S1, P1, Q1, A2, D2, S2, X2, AC, TC):
    sdict = globals()[fit_string]
    el_strain_array = np.zeros_like(time_array)
    creep_array_1 = np.zeros_like(time_array)
    creep_array_2 = np.zeros_like(time_array)
    KV1_amplitude = np.maximum(0.0,-A1*np.expm1(D1*(S1-sdict['stress_n'])))
    KV2_full_amplitude = A2*np.log(1-np.exp(-D2*S2)+np.exp(D2*(sdict['stress_n']-S2)))
    KV2_amplitude = np.copy(KV2_full_amplitude)
    constraint_start = 0
    for i in range(1,sdict['test_steps']):  # i = step-1
        # don't allow KV1 to fall back when stress lowered (plastic creep, straightening phase)
        KV1_amplitude[i] = max(KV1_amplitude[i], KV1_amplitude[i-1])
        # apply plastic creep constraint to KV2
        constraint_time = sdict['time_array'][sdict['step_starts'][i]] - constraint_start  # time to start of new step
        constraint_factor = max(0, min(1, (1-AC*(constraint_time-TC))))
        if(sdict['exceeding_steps'][i] == 1):
            KV2_amplitude[i] = KV2_amplitude[i-1] + constraint_factor * (KV2_full_amplitude[i]-KV2_full_amplitude[i-1])
            # constraint time restarts each time stress exceeds previous maximum
            constraint_start = sdict['time_array'][sdict['step_starts'][i]]  # start of new step
        else:
            KV2_amplitude[i] = KV2_amplitude[i-1]
    # time constants
    inv_tau1 = P1+sdict['stress_n']*Q1
    inv_tau2 = X2
    for step in range(1,fit_stop_step+1,1):  # step numbers to fit
        start_index = sdict['step_starts'][step-1] + 1  # don't include first point of step (at previous stress level)
        stop_index = sdict['step_starts'][step]
        stop_index += 1 if(step < sdict['test_steps']) else 0
            # include first point of next step (at current stress level), except for very last step
        neg_time_sub_array = time_array[start_index-1] - time_array[start_index:stop_index] # negative time since step start
        # elastic strain component
        el_strain_array[start_index:stop_index] = sdict['el_strain_est'][step-1]
        # creep component 1 - initial straightening
        base = creep_array_1[start_index-1] # last point calculated = first point of current step (0 at start of first step)
        target = KV1_amplitude[step-1]
        creep_array_1[start_index:stop_index] = base + (base - target) * np.expm1(inv_tau1[step-1] * neg_time_sub_array)
        # creep component 2 - plastic creep
        base = creep_array_2[start_index-1] # last point calculated = first point of current step (0 at start of first step)
        target = KV2_amplitude[step-1]
        creep_array_2[start_index:stop_index] = base + (base - target) * np.expm1(inv_tau2 * neg_time_sub_array)
    return A0 + el_strain_array + creep_array_1 + creep_array_2

def test_combined_creep_function(time_array, A0, A1, D1, S1, P1, Q1, A2, D2, S2, X2, FC, AR1, AR2, AR3, XR):
    sdict = globals()[fit_string]
    el_strain_array = np.zeros_like(time_array)
    creep_array_1 = np.zeros_like(time_array)
    creep_array_2 = np.zeros_like(time_array)
    creep_array_R = np.zeros_like(time_array)
    KV1_amplitude = np.maximum(0.0,-A1*np.expm1(D1*(S1-sdict['stress_n'])))
    KV2_full_amplitude = A2*np.log(1-np.exp(-D2*S2)+np.exp(D2*(sdict['stress_n']-S2)))
    KV2_amplitude = np.copy(KV2_full_amplitude)
    for i in range(1,sdict['test_steps']):  # i = step-1
        # don't allow KV1 to fall back when stress lowered (plastic creep, straightening phase)
        KV1_amplitude[i] = max(KV1_amplitude[i], KV1_amplitude[i-1])
        # apply plastic creep constraint to KV2
        constraint_factor = FC if(i == 15) else 1  # only apply to step 16
        if(sdict['exceeding_steps'][i] == 1):
            KV2_amplitude[i] = KV2_amplitude[i-1] + constraint_factor * (KV2_full_amplitude[i]-KV2_full_amplitude[i-1])
        else:
            KV2_amplitude[i] = KV2_amplitude[i-1]
    # time constants
    inv_tau1 = P1+sdict['stress_n']*Q1
    inv_tau2 = X2
    for step in range(1,fit_stop_step+1,1):  # step numbers to fit
        start_index = sdict['step_starts'][step-1] + 1  # don't include first point of step (at previous stress level)
        stop_index = sdict['step_starts'][step]
        stop_index += 1 if(step < sdict['test_steps']) else 0
            # include first point of next step (at current stress level), except for very last step
        neg_time_sub_array = time_array[start_index-1] - time_array[start_index:stop_index] # negative time since step start
        # elastic strain component
        el_strain_array[start_index:stop_index] = sdict['el_strain_est'][step-1]
        # creep component 1 - initial straightening
        base = creep_array_1[start_index-1] # last point calculated = first point of current step (0 at start of first step)
        target = KV1_amplitude[step-1]
        creep_array_1[start_index:stop_index] = base + (base - target) * np.expm1(inv_tau1[step-1] * neg_time_sub_array)
        # creep component 2 - plastic creep
        base = creep_array_2[start_index-1] # last point calculated = first point of current step (0 at start of first step)
        target = KV2_amplitude[step-1]
        creep_array_2[start_index:stop_index] = base + (base - target) * np.expm1(inv_tau2 * neg_time_sub_array)
        # creep component 3 - recoverable creep
        if(sdict['exceeding_steps'][step-1] == 0):  # no recoverable creep contribution during exceeding steps
            if(sdict['exceeding_steps'][step-2] == 1):
                # reset variables if this is the first non-exceeding step
                extra_stress = 0.0
                inv_ER = AR1 if(step < 6) else AR2 if(step < 10) else AR3
            extra_stress += sdict['stress_n_step'][step-1]
            target = extra_stress * inv_ER
            base = creep_array_R[start_index-1] 
            # last point calculated = first point of current step (0 at start of each non-exceeding step sequence)
            creep_array_R[start_index:stop_index] = base + (base - target) * np.expm1(XR * neg_time_sub_array)
    return A0 + el_strain_array + creep_array_1 + creep_array_2 + creep_array_R

def multi_step_fit(fit_string_value, stop_step, fit_last):
    global fit_string
    global fit_stop_step
    global fitted_stop_step
    global time_sub_array
    global strain_sub_array
    global fitted_curve
    global el_strain_array
    global creep_array_1
    global creep_array_2
    global tail_array
    global check_array
    global creep_array_R
    global check_array
    #
    fit_string = fit_string_value
    sdict = globals()[fit_string]
    fit_stop_step = stop_step - (1 if(stop_step == sdict['test_steps'] and not fit_last) else 0)  
        # don't fit to last step where string broke unless fit_last == True
    print('\nFitting string', fit_string, 'steps 1 to', fit_stop_step)
    start_index = sdict['step_starts'][0]
    stop_index = sdict['step_starts'][fit_stop_step]
    stop_index += 1 if(fit_stop_step < sdict['test_steps']) else 0
    time_sub_array = sdict['time_array'][start_index:stop_index]
    strain_sub_array = sdict['strain_array'][start_index:stop_index]
    time_for_fitting = time_sub_array - sdict['time_array'][start_index]
    #
    # curve fitting
    if(fit_string == 'N28m'):
        popt, pcov = curve_fit(test_combined_creep_function_28m, time_for_fitting, strain_sub_array, p0 = sdict['p0_28m'],
                               bounds=([-0.5,0,0,0,0,0,0,0,0,-10,0,10],[0.5,1,1,50,1,1,1,1,3000,50,1,50]))
    else:
        popt, pcov = curve_fit(test_combined_creep_function, time_for_fitting, strain_sub_array, p0 = sdict['p0'],
                               bounds=([-0.5,0,0,0,0,0,0,0,0,-10,0,0,0,0,0],
                                       [0.5,1,1,50,1,1,1,1,3000,50,1,0.1,0.1,0.1,50]))
    print(popt)
    fitted_stop_step = fit_stop_step
    #
    # reconstruct fitted components
    print('\nPlotting string', fit_string, 'steps 1 to', stop_step)
    stop_index = sdict['step_starts'][stop_step]
    stop_index += 1 if(stop_step < sdict['test_steps']) else 0
    time_sub_array = sdict['time_array'][start_index:stop_index]
    strain_sub_array = sdict['strain_array'][start_index:stop_index]
    time_for_fitting = time_sub_array - sdict['time_array'][start_index]
    # evaluate fitted curve over full plot range
    fit_stop_step = stop_step
    if(fit_string == 'N28m'):
        fitted_curve = test_combined_creep_function_28m(time_for_fitting, *popt)
        # components are: A0, A1, D1, S1, P1, Q1, A2, D2, S2, X2, AC, TC
    else:
        fitted_curve = test_combined_creep_function(time_for_fitting, *popt) 
        # components are: A0, A1, D1, S1, P1, Q1, A2, D2, S2, X2, FC, AR1, AR2, AR3, XR
    el_strain_array = np.zeros_like(time_for_fitting)
    creep_array_1 = np.zeros_like(time_for_fitting)
    creep_array_2 = np.zeros_like(time_for_fitting)
    creep_array_R = np.zeros_like(time_for_fitting)
    KV1_amplitude = np.maximum(0.0,-popt[1]*np.expm1(popt[2]*(popt[3]-sdict['stress_n'])))
    KV2_full_amplitude = popt[6]*np.log(1-np.exp(-popt[7]*popt[8])+np.exp(popt[7]*(sdict['stress_n']-popt[8])))
    KV2_amplitude = np.copy(KV2_full_amplitude)
    constraint_start = 0
    for i in range(1,sdict['test_steps']):  # i = step-1
        # don't allow KV1 to fall back when stress lowered (plastic creep, straightening phase)
        KV1_amplitude[i] = max(KV1_amplitude[i], KV1_amplitude[i-1])
        # apply plastic creep constraint to KV2
        if(fit_string == 'N28m'):
            constraint_time = sdict['time_array'][sdict['step_starts'][i]] - constraint_start  # time to start of new step
            constraint_factor = max(0, min(1, (1-popt[10]*(constraint_time-popt[11]))))
            print(i, constraint_time, constraint_factor)
        else:
            constraint_factor = popt[10] if(i == 15) else 1  # only apply to step 16
        if(sdict['exceeding_steps'][i] == 1):
            KV2_amplitude[i] = KV2_amplitude[i-1] + constraint_factor * (KV2_full_amplitude[i]-KV2_full_amplitude[i-1])
            # constraint time restarts each time stress exceeds previous maximum
            constraint_start = sdict['time_array'][sdict['step_starts'][i]]  # start of new step
        else:
            KV2_amplitude[i] = KV2_amplitude[i-1]
    # time constants
    inv_tau1 = popt[4]+sdict['stress_n']*popt[5]
    inv_tau2 = popt[9]
    for step in range(1,stop_step+1,1):  # step numbers to fit
        start_index = sdict['step_starts'][step-1] + 1  # don't include first point of step (at previous stress level)
        stop_index = sdict['step_starts'][step]
        stop_index += 1 if(step < sdict['test_steps']) else 0
            # include first point of next step (at current stress level), except for very last step
        neg_time_sub_array = time_for_fitting[start_index-1] - time_for_fitting[start_index:stop_index] # negative time since step start
        el_strain_array[start_index:stop_index] = sdict['el_strain_est'][step-1]
        # creep component 1 - initial straightening
        base = creep_array_1[start_index-1] # last point calculated = first point of current step (0 at start of first step)
        target = KV1_amplitude[step-1]
        creep_array_1[start_index:stop_index] = base + (base - target) * np.expm1(inv_tau1[step-1] * neg_time_sub_array)
        # creep component 2 - plastic creep
        base = creep_array_2[start_index-1] # last point calculated = first point of current step (0 at start of first step)
        target = KV2_amplitude[step-1]
        creep_array_2[start_index:stop_index] = base + (base - target) * np.expm1(inv_tau2 * neg_time_sub_array)
        # creep component 3 - recoverable creep
        if((fit_string != 'N28m') and (sdict['exceeding_steps'][step-1] == 0)):  
            # no recoverable creep contribution during exceeding steps
            if(sdict['exceeding_steps'][step-2] == 1):
                # reset variables if this is the first non-exceeding step
                extra_stress = 0.0
                inv_ER = popt[11] if(step < 6) else popt[12] if(step < 10) else popt[13]
            extra_stress += sdict['stress_n_step'][step-1]
            target = extra_stress * inv_ER
            base = creep_array_R[start_index-1] 
            # last point calculated = first point of current step (0 at start of each non-exceeding step sequence)
            creep_array_R[start_index:stop_index] = base + (base - target) * np.expm1(popt[14] * neg_time_sub_array)
    check_array = popt[0] + el_strain_array + creep_array_1 + creep_array_2 + creep_array_R
    return popt


def multi_step_fit_plot_ax(ax, fit_string_value, stop_step, fit_last):
    # run curve fitting
    popt = multi_step_fit(fit_string_value, stop_step, fit_last)
    # initial data
    ax.plot(time_sub_array, strain_sub_array, 'k-', label='strain data')
    ax.plot(time_sub_array[1:], fitted_curve[1:], 'r--', label='fitted curve')
#    ax.plot(time_sub_array[1:], check_array[1:], 'b--', label='check curve')
    # plot component parts of fitted curve
    plot_offset = popt[0]+el_strain_array[1]  # el_strain_array[0] = 0 so need later value
#    ax.plot(time_sub_array, [popt[0] for i in range(len(time_sub_array))], 'k:', label='offset')
#    ax.plot(time_sub_array, popt[0]+el_strain_array, 'c:', label='spring')
    ax.plot(time_sub_array, plot_offset+creep_array_1, 'k:', label='1st K-V stage')
    ax.plot(time_sub_array, plot_offset+creep_array_2, 'r:', label='2nd K-V stage')
    if(fit_string_value != 'N28m'):
        ax.plot(time_sub_array, plot_offset+creep_array_R, 'b:', label='Recoverable creep')
    ax.set_title('String ' + str(fit_string_value) + ', fitted steps 1 to ' + str(fitted_stop_step))
    ax.set_xlabel('Time (days)')
    ax.set_ylabel('Strain')
    ax.legend(loc='best', prop={'size':10}, framealpha=1)

def multi_step_fit_plot(fit_string_value, stop_step, fit_last, save_plot):
    # plot data    
    fig=plt.figure(figsize=(8,5))
    ax1=fig.add_subplot(111)
    multi_step_fit_plot_ax(ax1, fit_string_value, stop_step, fit_last)
    fig.tight_layout()
    if(save_plot):
        savefile = save_path + "plastic_creep_multi_step_" + fit_string_value + '_1_to_' \
                    + str(stop_step) + '.' + save_format
        print("\n%s" % (savefile))
        fig.savefig(savefile, format=save_format, dpi=1000)
    plt.show()


N28m['p0_28m'] = [0,0.052,0.025,24,0,0.056,1,0.0071,550,0.28,0.08,35]

N28['p0'] = [0,0.047,0.026,25,0,0.068,1,0.0056,620,0.25,0.79,0.00016,0.00014,0.00006,1.1]


fig=plt.figure(figsize=fig_size_2)
ax1=fig.add_subplot(121)
ax2=fig.add_subplot(122)
multi_step_fit_plot_ax(ax1, 'N28m', 18, False)
multi_step_fit_plot_ax(ax2, 'N28', 18, False)
ax1.axis([-8,158,-0.008,0.19])
ax2.axis([-8,158,-0.008,0.19])
fig.tight_layout()
ax1.text(0.9, 0.07,'(a)', transform=ax1.transAxes)
ax2.text(0.9, 0.15,'(b)', transform=ax2.transAxes)
savefile = save_path + "plastic_creep_multi_step_N28." + save_format
print("\n%s" % (savefile))
#fig.savefig(savefile, format=save_format, dpi=1000)
plt.show()


# individual plots
# ================
# N28m
multi_step_fit_plot('N28m', 17, False, False)
multi_step_fit_plot('N28m', 18, False, False)
multi_step_fit_plot('N28m', 18, True, False)
# N28
multi_step_fit_plot('N28', 17, False, False)
multi_step_fit_plot('N28', 18, False, False)
multi_step_fit_plot('N28', 18, True, False)
```

```
Fitting string N28m steps 1 to 17
[-8.73759409e-04  5.14508351e-02  2.51271490e-02  2.37002065e+01
  1.71641658e-39  5.58634764e-02  1.00000000e+00  7.08631757e-03
  5.52253113e+02  2.76661587e-01  8.12533939e-02  3.49671249e+01]

Plotting string N28m steps 1 to 18
1 13.652268518518518 1
2 10.11224537037037 1
3 7.941400462962964 1
4 14.914062500000004 1
5 21.108414351851852 1
6 6.825185185185184 1
7 7.072800925925925 1
8 14.057037037037034 1
9 28.944710648148146 1
10 7.04800925925926 1
11 11.944421296296298 1
12 17.95364583333334 1
13 25.98393518518519 1
14 30.00038194444444 1
15 38.99905092592593 0.6723923237383904
16 17.91071759259259 1
17 5.993483796296289 1

Fitting string N28 steps 1 to 17
[-7.53180267e-05  4.66889401e-02  2.63039216e-02  2.50577525e+01
  5.98073256e-13  6.94978610e-02  1.00000000e+00  5.59885147e-03
  6.15900962e+02  2.90796809e-01  7.94233635e-01  1.56225790e-04
  1.43576614e-04  6.26018740e-05  1.15206703e+00]

Plotting string N28 steps 1 to 18

./plastic_creep_multi_step_N28.eps
```

```
Fitting string N28m steps 1 to 17
[-8.73759409e-04  5.14508351e-02  2.51271490e-02  2.37002065e+01
  1.71641658e-39  5.58634764e-02  1.00000000e+00  7.08631757e-03
  5.52253113e+02  2.76661587e-01  8.12533939e-02  3.49671249e+01]

Plotting string N28m steps 1 to 17
1 13.652268518518518 1
2 10.11224537037037 1
3 7.941400462962964 1
4 14.914062500000004 1
5 21.108414351851852 1
6 6.825185185185184 1
7 7.072800925925925 1
8 14.057037037037034 1
9 28.944710648148146 1
10 7.04800925925926 1
11 11.944421296296298 1
12 17.95364583333334 1
13 25.98393518518519 1
14 30.00038194444444 1
15 38.99905092592593 0.6723923237383904
16 17.91071759259259 1
17 5.993483796296289 1
```

```
Fitting string N28m steps 1 to 17
[-8.73759409e-04  5.14508351e-02  2.51271490e-02  2.37002065e+01
  1.71641658e-39  5.58634764e-02  1.00000000e+00  7.08631757e-03
  5.52253113e+02  2.76661587e-01  8.12533939e-02  3.49671249e+01]

Plotting string N28m steps 1 to 18
1 13.652268518518518 1
2 10.11224537037037 1
3 7.941400462962964 1
4 14.914062500000004 1
5 21.108414351851852 1
6 6.825185185185184 1
7 7.072800925925925 1
8 14.057037037037034 1
9 28.944710648148146 1
10 7.04800925925926 1
11 11.944421296296298 1
12 17.95364583333334 1
13 25.98393518518519 1
14 30.00038194444444 1
15 38.99905092592593 0.6723923237383904
16 17.91071759259259 1
17 5.993483796296289 1
```

```
Fitting string N28m steps 1 to 18
[-9.79691800e-04  5.26342847e-02  2.46916311e-02  2.34666952e+01
  2.83348598e-14  5.36938668e-02  9.99997535e-01  7.36207435e-03
  5.43646491e+02  2.75088645e-01  8.16414182e-02  3.48897334e+01]

Plotting string N28m steps 1 to 18
1 13.652268518518518 1
2 10.11224537037037 1
3 7.941400462962964 1
4 14.914062500000004 1
5 21.108414351851852 1
6 6.825185185185184 1
7 7.072800925925925 1
8 14.057037037037034 1
9 28.944710648148146 1
10 7.04800925925926 1
11 11.944421296296298 1
12 17.95364583333334 1
13 25.98393518518519 1
14 30.00038194444444 1
15 38.99905092592593 0.664509488333577
16 17.91071759259259 1
17 5.993483796296289 1
```

```
Fitting string N28 steps 1 to 17
[-7.53180267e-05  4.66889401e-02  2.63039216e-02  2.50577525e+01
  5.98073256e-13  6.94978610e-02  1.00000000e+00  5.59885147e-03
  6.15900962e+02  2.90796809e-01  7.94233635e-01  1.56225790e-04
  1.43576614e-04  6.26018740e-05  1.15206703e+00]

Plotting string N28 steps 1 to 17
```

```
Fitting string N28 steps 1 to 17
[-7.53180267e-05  4.66889401e-02  2.63039216e-02  2.50577525e+01
  5.98073256e-13  6.94978610e-02  1.00000000e+00  5.59885147e-03
  6.15900962e+02  2.90796809e-01  7.94233635e-01  1.56225790e-04
  1.43576614e-04  6.26018740e-05  1.15206703e+00]

Plotting string N28 steps 1 to 18
```

```
Fitting string N28 steps 1 to 18
[-1.68884726e-04  4.81253539e-02  2.58016611e-02  2.47876873e+01
  2.96692226e-12  6.55001997e-02  9.99984623e-01  5.95755049e-03
  5.99755018e+02  2.91166029e-01  7.79739857e-01  1.54452925e-04
  1.41909573e-04  6.32537326e-05  1.18832048e+00]

Plotting string N28 steps 1 to 18
```

## Stress-Strain comparison¶

In [12]:

```
show_strain = True  # if set False, plots length adjustment instead

fig1=plt.figure(figsize=fig_size_1)
ax1=fig1.add_subplot(111)

width = 1
y_param = 'strain_array' if(show_strain) else 'length_adj_array'
for string in (string_list+['N8']):
    sdict = globals()[string]
    stress0 = np.append(0, sdict['stress_n'][:])
    if(string == 'N8'):
        y_data = np.append(0, (N8['strain_array'] if(show_strain) else N8['length_adj']))
        ax1.plot(stress0, y_data, '-.o', color=sdict['pen'], lw=width, ms=4, label=sdict['leg'])
        ax1.plot(stress0[-1], y_data[-1], 'x', color=sdict['pen'], ms=6, mew=3)
    else:
        y_data = np.append(0, np.array([sdict[y_param][sdict['step_starts'][i]-1] for i in sdict['step_range']]))
        # plot markers
        ax1.plot(stress0, y_data, 'o', color=sdict['pen'], ms=4)
        # plot lines
        for step in sdict['step_range']:
            style = '-' if(sdict['exceeding_steps'][step-1]==1) else '--' if(sdict['matching_steps'][step-1]==1) else ':'
            ax1.plot([stress0[step-1],stress0[step]], [y_data[step-1],y_data[step]], ls=style, color=sdict['pen'], lw=width)
        # add legend label
        ax1.plot([0,10],[-100,-100],'-o', color=sdict['pen'], lw=width, label=sdict['leg'])
# add breaking point markers
# N8 already done above
for string in ['N7','N26','N28','N34','N33']:
    sdict = globals()[string]
    ax1.plot(sdict['stress_n'][-1], sdict[y_param][-1], 'x', color=sdict['pen'], ms=6, mew=3)
#        
ax1.axis([-15,300,-0.01,0.19])
ax1.set_xlabel('Stress (MPa)')
ax1.set_ylabel('Strain' if(show_strain) else 'Length adjustment (m)')
ax1.legend(loc='lower right', ncol=3, prop={'size':10}, framealpha = 1)
plt.tight_layout()
save_title = "strain_stress." if(show_strain) else "length_stress."
savefile = save_path + save_title + save_format
print("%s" % (savefile))
#plt.savefig(savefile, format=save_format, dpi=1000)
plt.show()
```

```
./strain_stress.eps
```

## Thermal compensation for stress steps¶

In [13]:

```
fig=plt.figure(figsize=fig_size_2)
ax1=fig.add_subplot(121)
ax2=fig.add_subplot(122)

# creep change versus temperature change
creep_step_array = N7['length_adj_array_raw'][1:] - N7['length_adj_array_raw'][:-1]
temperature_step_array = N7['temperature_array'][1:] - N7['temperature_array'][:-1]
start_index = 133  # steady state creep range for N7 step 1
stop_index = 299   # last data point
x_range = [-1.2,1.2]
y_range = [-0.013,0.02]
ax1.plot(x_range, [0,0], 'k--', lw=1)
ax1.plot([0,0], y_range, 'k--', lw=1)
ax1.plot(temperature_step_array[start_index:stop_index], 1000 * creep_step_array[start_index:stop_index], 
         color=N7['pen'], ls='none', marker='x', ms=3, mfc='none')
fit = poly.polyfit(temperature_step_array[start_index:stop_index], 1000 * creep_step_array[start_index:stop_index], 1)
ax1.plot(x_range, poly.polyval(x_range, fit), 'k--', lw=1)
gradient = fit[1]
print("N7 step 1 thermal adj. rate = %.15f mm/°C" % (gradient))
ax1.axis([-1.3,1.3,-0.015,0.022])
ax1.set_xlabel('Temperature change (°C)')
ax1.set_ylabel('Length adjustment change (mm)')

# thermal adjustment gradients versus stress
ax2.plot(N7['stress_n'], N7['adj_rate'], color=N7['pen'], ls='none', marker='o', mec=N7['pen'], 
         mfc='none', mew=1, label=N7['leg'])
ax2.plot(N8['stress_n'], N8['adj_rate'], color=N8['pen'], ls='none', marker='o', mec=N8['pen'], 
         mfc='none', mew=1, label=N8['leg'])
avge = np.average(N8['adj_rate'][:-1])  # exclude last point for which no data
print('N8 average adjustment rate = %.15f mm/°C' % (avge))
ax2.plot([0,300],[avge,avge], color=N8['pen'], ls='--', lw=1)
ax2.legend(loc='lower right', ncol = 1, framealpha = 1)
ax2.set_xlabel('Stress (MPa)')
ax2.set_ylabel("Thermal adjustment rate (mm/°C)")

fig.tight_layout()
ax1.text(0.9, 0.9,'(a)', transform=ax1.transAxes)
ax2.text(0.9, 0.9,'(b)', transform=ax2.transAxes)
savefile = save_path + "thermal_comp_gradients." + save_format
print("\n%s" % (savefile))
#fig.savefig(savefile, format=save_format, dpi=1000)
plt.show()
```

```
N7 step 1 thermal adj. rate = -0.012744931220530 mm/°C
N8 average adjustment rate = -0.022021225791555 mm/°C

./thermal_comp_gradients.eps
```

In [ ]:

```

```
